# Supplementary material for: Ultrafast small-scale soft electromagnetic robots
Source: Nat Commun. 2022 Aug 9;13:4456. doi: 10.1038/s41467-022-32123-4 (PMC9363453; doi:10.1038/s41467-022-32123-4)
Supplement: Supplementary file 1 — Supplementary Information [file 41467_2022_32123_MOESM1_ESM.pdf]

Supplementary Materials for

Ultrafast small-scale soft electromagnetic robots

Guoyong Mao[1,†*], David Schiller[1,2†], Doris Danninger[1,2], Bekele Hailegnaw[1,2], Florian Hartmann[1,2], Thomas Stockinger[1,2], Michael Drack[1,2], Nikita Arnold[1,2], Martin Kaltenbrunner[1,2*]

*Corresponding author. E-mail: guoyong.mao@jku.at or martin.kaltenbrunner@jku.at
†These authors contributed equally to this work.

This PDF file includes:

Supplementary Text
Supplementary Figs. 1 to 45
Supplementary Tables 1 to 7
Supplementary References

Other Supplementary Materials for this manuscript include the following:

Videos S1 to S10

Table of Contents

1. Supplementary Text 4
  1.1. Numerical simulation of the bilayer films 4
  1.2. Theory of curved bilayer beam 4
  1.3. Calculation of the Lorentz force 5
  1.4. Comparison between the bilayer and monolayer films 5
  1.5. Thickness of the SEMR TST in vibration test 6
  1.6. Effect of gravity 6
  1.7. Design and fabrication of the 3D printed substrate 7
  1.8. Design and fabrication of SEMRs 7
    1.8.1. Performance of the 3D LM printer 7
    1.8.2. Feet design 7
    1.8.3. SEMR TST/TL 8
    1.8.4. SEMR TSTS 8
    1.8.5. SEMR TRC 9
    1.8.6. SEMR UL 9
    1.8.7. SEMR UR1 9
    1.8.8. SEMR UR2 10
  1.9. Planar vibrations of a curved beam 11
  1.10. Oscillator approximation and velocity 13
  1.11. Square-wave (SW) excitation 14
  1.12. Speed estimations for a hovering robot 17
  1.13. Vertical acceleration and the start of the movement 18
  1.14. Estimations of the damping coefficient 19
  1.15. Parameter used in analytic calculations 20
  1.16. Takeaways from the theoretical analysis 20
  1.17. Locomotion principle of the SEMR with the L-shaped feet 21
  1.18. Characterization of the magnetic field 22
  1.19. Joule heating and further performance optimization 22
  1.20. Appendix: auxiliary theoretical expressions 23
2. Supplementary Figures 25
  Supplementary Fig. 1. Comparison of the maximum relative swimming speeds of various animals and robots 25
  Supplementary Fig. 2. LM printing system. 26
  Supplementary Fig. 3. Characterization of the LM printing system 27
  Supplementary Fig. 4. Connection of electrodes and LM channels. 28
  Supplementary Fig. 5. Geometries of SEMA 1 and SEMA 2. 29
  Supplementary Fig. 6. PWM control system for the robots. 30
  Supplementary Fig. 7. Bending test for millimeter-scale SEMAs 31
  Supplementary Fig. 8. Temperature measurement of SEMA 1. 32
  Supplementary Fig. 9. Free body diagram of a coil carrying a current in a homogenous magnetic field 33
  Supplementary Fig. 10. Deformation of a prestretched bilayer beam. 34
  Supplementary Fig. 11. Fabrication of a curved bilayer film. 35
  Supplementary Fig. 12. Shapes of bilayer films with different prestretches. 36
  Supplementary Fig. 13. Fabrication of the curved SEMR TST 37
  Supplementary Fig. 14. Geometry of the SEMR TST 38
  Supplementary Fig. 15. Static loading test of the SEMR TST. 39
  Supplementary Fig. 16 Vibration test of the SEMR TST with sinusoidal currents 40
  Supplementary Fig. 17. Vibration test of the SEMR TST with square-wave currents 41
  Supplementary Fig. 18. Performance of different 3D-printed substrates. 42
  Supplementary Fig. 19 Free body diagram analysis of the robot on the sawtooth-shaped substrate. 43
  Supplementary Fig. 20. Maximum speed of the SEMR TST. 44
  Supplementary Fig. 21. Representative curves of displacements and velocities vs. time of SEMR TST 45

Supplementary Fig. 22. Strategies for stable locomotion at high currents. 46
Supplementary Fig. 23. Motion mechanism of the SEMR with L-shaped feet. 47
Supplementary Fig. 24. Design and performance of L-shaped feet. 48
Supplementary Fig. 25. Performance of SEMR TL on various substrates. 49
Supplementary Fig. 26. Compression test of the body of SEMR TL. 50
Supplementary Fig. 27. Jumps of SEMR TST and TL. 51
Supplementary Fig. 28. Photo and control schematic of the two-module SEMR TSTS. 52
Supplementary Fig. 29. Design and function of transport SEMR TRC. 53
Supplementary Fig. 30. Small PCB used for driving the SEMR UL. 54
Supplementary Fig. 31. Medium PCB used for driving the SEMR UR1. 55
Supplementary Fig. 32. Large PCB used for driving the SEMR UR2. 56
Supplementary Fig. 33. Design and characterization of SEMR UL. 57
Supplementary Fig. 34. Design and characterization of SEMR UR1 58
Supplementary Fig. 35 Design and characterization of SEMR UR2 59
Supplementary Fig. 36. Properties of the elastomer. 60
Supplementary Fig. 37. Deformation of curved bilayer films of different designs. 61
Supplementary Fig. 38. Comparison of deformation of a bilayer and a monolayer film with the same thickness. 62
Supplementary Fig. 39. Effective modulus of the equivalent curved monolayer film. 63
Supplementary Fig. 40. Free oscillation decay test for the SEMR. 64
Supplementary Fig. 41. Resonance curves for the curved-beam robot with free-free and clamped-free boundary conditions. 65
Supplementary Fig. 42. Square-wave response, higher vibration modes, and running speed of the robot. 66
Supplementary Fig. 43. Square-wave and sinusoidal responses of a Lorentzian harmonic oscillator. 67
Supplementary Fig. 44. Comparison of temporal responses to a sinusoidal and SW excitation for $\omega = \omega_1 / 5$, where one of the low-frequency resonances lies. 68
Supplementary Fig. 45. Magnetic fields near the surfaces of two magnets. 69
3. Supplementary Tables 70
Supplementary Table 1. Comparison of the maximum running speeds of insects, mammals, rigid/flexible robots and soft robots. 70
Supplementary Table 2. Comparison of the maximum swimming speeds of animals and robots. 71
Supplementary Table 3. Parameters of 3D printed feet 72
Supplementary Table 4. Parameters of the non-magnetic battery 73
Supplementary Table 5. Summary of the SEMRs 74
Supplementary Table 6. Parameters of spin - coating process. 75
Supplementary Table 7. Lorentzian oscillator parameters for representative curved beam geometries. 76
4. Supplementary References 77

1. Supplementary Text

1.1. Numerical simulation of the bilayer films

We utilize the user subroutine UMAT of the finite element software ABAQUS to introduce the prestretches to the hyperelastic bilayer beam. The theoretical framework of this method is described in most continuum mechanics textbooks[2]. The general motion of a continuum is described as

$$\mathbf{x} = \mathbf{x}(\mathbf{X}, t) \tag{1}$$

in which $\mathbf{x}$ is the spatial position at time t with materials coordinate $\mathbf{X}$ of a material particle. Small changes in the position of the reference and current configuration are linearly related:

$$d\mathbf{x} = \mathbf{F} d\mathbf{X} \tag{2}$$

where $\mathbf{F}$ is the deformation gradient tensor which can be calculated by

$$\mathbf{F} = \nabla \mathbf{x} \tag{3}$$

Here, the derivatives with respect to $\mathbf{X}$ are implied. We build the model as shown in Supplementary Fig. 10b, where the prestretch is applied to the top layer. When the initial thickness of the top layer is 1 mm and is applied with a prestretch of 2 in the y-direction, we build the top layer with a thickness of 0.5 mm. Then we set the deformation gradient directly via UMAT and update $\mathbf{x}$. This finite element method technique is often used for dielectric elastomers[3]. In the UMAT, an eight-node brick element (C3D8) is used.

In the simulation, the thickness of the bottom layer and the initial thickness of the top layer are both 1 mm. The length of the bilayer is 20 mm. The boundary conditions are illustrated in Supplementary Fig. 10b - the left end of the bilayer is bonded and the right one is free.

1.2. Theory of curved bilayer beam

In this section, we analyze the roles of parameters of the bilayer beam in tuning the curvature of bending using a simple linear elastic model. We consider a plane-strain case of a bilayer beam made of two linear elastic materials with Young's moduli E_1 and E_2, and different pre-strains in the y-direction, ε_{10} and ε_{20}, for the top and bottom layer respectively (Supplementary Fig. 10c). The out-of-plane width of the bilayer beam is set to b. For simplicity, we set the pre-strain of the bottom layer to $\varepsilon_{20} = 1$. Then the initial lengths without deformation of both top and bottom layers are L_{10} and L_{20} and they have the simple relationship $L_{20} = (1+\varepsilon_{10})L_{10}$ as shown in (Supplementary Fig. 10c). The pre-strained top layer is bonded to the bottom layer and the bilayer beam is curved (Supplementary Fig. 10d).

According to the force and moment balance between the inner force and moment of the bilayer beam, we can obtain the following equations,

$$N_{11} + N_{22} = 0 \tag{4}$$

$$M_{11} + M_{22} = -N_{11} t_{11}/2 + N_{22} t_{22}/2 \tag{5}$$

Here, N_{11}, M_{11} and t_{11} are the resultant force, moment, and thickness of the top layer, respectively, while N_{22}, M_{22} and t_{22} correspond to those of the bottom layer. Assuming that the radius of curvature is much larger than the thickness of the bilayer beam, both the top and bottom layers have the same curvature of $1/r$. Using Euler-Bernoulli beam theory we obtain $M_{11} = E_1 I_1 / r$ and $M_{22} = E_2 I_2 / r$, where I_1 and I_2 are the area moments of inertia of the two layers $I_1 = b t_{11}^3/12$ and $I_2 = b t_{22}^3/12$. Strain compatibility at the interface (calculated from the neutral plane in each layer) requires that

$$\frac{N_{11}}{bE_1 t_{11}} + \varepsilon_{10} - \frac{t_{11}}{2r} = \frac{N_{22}}{bE_2 t_{22}} + \frac{t_{22}}{2r} \tag{6}$$

Combining Eqs. (4)-(6)and substituting the expressions for the moments and area moments of inertia gives the following expression for the radius of the bending curvature,

$$r = \frac{1}{6\varepsilon_{10}}\left(3\left(t_{11}+t_{22}\right) + \frac{E_1 t_{11}^3 + E_2 t_{22}^3}{t_{11}+t_{22}}\left(\frac{1}{E_1 t_{11}} + \frac{1}{E_2 t_{22}}\right)\right) \tag{7}$$

If we further assume that the thicknesses and deformations of both layers are small and approximate the deformed thicknesses by their initial values $t_{11} = t_{10}$ and $t_{22} = t_{20}$, we obtain

$$r = \frac{1}{6\varepsilon_{10}}\left(3\left(t_{10} + t_{20}\right) + \frac{E_1 t_{10}^{\;3} + E_2 t_{20}^{\;3}}{t_{10} + t_{20}}\left(\frac{1}{E_1 t_{10}} + \frac{1}{E_2 t_{20}}\right)\right) \tag{8}$$

If the material of both the top and the bottom layers is the same, the expression for the radius simplifies to:

$$r = \frac{1}{6\varepsilon_{10}}\left(\frac{\left(t_{10} + t_{20}\right)^3}{t_{10} t_{20}}\right) \tag{9}$$

When the initial thicknesses of both layers are equal, $t_{10} = t_{20}$, we obtain the following simple expression:

$$r = \frac{4t_{10}}{3\varepsilon_{10}} \tag{10}$$

1.3. Calculation of the Lorentz force

The Lorentz force exerted by the magnetic field $\mathbf{B}$ onto the current I in direction $d\mathbf{L}$ is given by the vector product $Id\mathbf{L} \times \mathbf{B}$. The geometry of coil currents with respect to the magnetic field is shown in the upper panel of the Fig. 3a. The forces F_A and F_B onto each (horizontal) lateral element of the wires on the opposite sides point in the opposite directions. For a robot, curved as shown in the Fig. 3b, this leads only to the lateral stretching/compression, which requires much higher forces than bending of a thin elastic plate (see Supplementary Fig. 9 and the related discussion in the main text). Likewise, the forces F_{1L} and F_{2R} onto the opposite currents near the central (top) parts of the robot point in the opposite directions and do not influence bending.

Only the (almost) diametrically opposite forces F_{1R} and F_{2L} are responsible for bending and relevant for the subsequent consideration (see Fig. 3b). The one-leg force is parallel to the direction of robot motion and is calculated as $F = BIL$, where L is the total length of all parallel wire segments at one of the diametrically opposite edges of the robot body. The *z*-component of the magnetic field dominates, and is almost constant near the surface. Its measured values can be approximated by a linear fit $B_z = -5.5385Z + 343.4$ mT for magnet 1 (see details in ref.[4]), where Z [mm] is the distance from the surface of the magnet and B_z is given in [mT]. The field variation over the robot height (~5 mm between the feet and the top) is in the range of 308-336 mT; this takes into account the substrate thickness (1.4 mm for the substrate with a = 0.8 mm). In estimations we use the value $B = 0.3\,\mathrm{T}$. The robot feet are indicated by the short slanted segments in Fig. 3c and black polylines in Fig. 3f. The lengths of the three LM segments near these feet are 5, 6, and 7 mm. As an example, an applied current of 0.3 A results in a Lorentz force of $F = 0.3\,\mathrm{A} \times 0.3\,\mathrm{T} \times (5{+}6{+}7)\ \mathrm{mm}{=}1.62\ \mathrm{mN}$.

1.4. Comparison between the bilayer and monolayer films

To understand the differences and find the effective parameters for use in analytic considerations, we performed comparative numerical simulations with both bilayer and five pre-cast monolayer films, which are curved in the relaxed state. All the films have the same cross-section 9 mm ×1 mm (for the bilayer film, it is in a bent state). The simulation domain includes only half of the film for symmetry reasons. Different simulated films have the following characteristics:

- Bilayer film: the original thickness of the prestretched layer (prestretch is 1.3) is 350 μm and the total thickness is 1 mm. The half-arc length of the bilayer film is 8 mm, with a width of 9 mm, which corresponds to a half-arc angle of 99.74°. Neo-Hookean hyperelastic model is used.
- Monolayer 1: The geometry is identical to that of the bilayer film in a bent state. Neo-Hookean hyperelastic model is used.
- Monolayer 2: The radius of the film is 4.8 mm with a half-arc length of 7.54 mm (inset in Supplementary Fig. 38b), which corresponds to a half-arc angle of 90°. Neo-Hookean hyperelastic model is used.

- Monolayer 3: The radius of the film is 4.8 mm with a half-arc length of 8.36 mm (inset in Supplementary Fig. 38b), which corresponds to a half arc angle of 99.74º. Neo-Hookean hyperelastic model is used.
- Monolayer 3L: Identical geometry to that of monolayer 3 but with a linear elastic material model in simulation. The shear modulus is the same as that in the neo-Hookean hyperelastic model and the Poisson's ratio is set to be 0.49.

In Supplementary Fig. 38a, we show the load capacity of the robot with bilayer design and monolayer design. We apply a vertical force at the top of the robot. Results show that the bilayer film withstands a larger load at the same deformation, which means it is stiffer. We also apply a horizontal force to different kinds of curved films in Supplementary Fig. 38b. The results also reveal that the material model (hyperelastic or linear) is of minor importance for the simulation of the SEMR as long as its deformation remains small.

In the ensuing theoretical analysis, we treat the curved robot beam as homogeneous. This poses a question about the effective stiffness of a bilayer, when it is described as a monolayer. To answer this question, we simulated the horizontal deflection of a realistic 3D quarter-circle bilayer (clamped at the top) and a (planar) pre-curved monolayer of exactly the same thickness. The results, presented in Supplementary Fig. 39, imply that for our geometry, layer thicknesses and pre-stretch, the effective shear modulus of a bilayer for small deflections is 1.23 times larger than the modulus of an unstretched elastomer. This led to a choice of $\mu = 80\,\mathrm{kPa}$, which was used for the estimations of resonant frequencies and damping coefficient in the subsequent sections.

1.5. Thickness of the SEMR TST in vibration test

The elastomer used for fabrication is a mixture of PDMS (density $0.965\,\mathrm{gcm^{-3}}$) and Ecoflex 00-30 solution (density $1.07\,\mathrm{gcm^{-3}}$) with a mass ratio of 1:10. The density of the elastomer is estimated as $0.965\times0.1+1.07\times0.9\ \mathrm{g\ cm^{-3}} = 1.06\ \mathrm{g\ cm^{-3}}$. The density of the LM is $6.44\ \mathrm{g\ cm^{-3}}$. The cross-section area of the LM channel is 0.0203 $\mathrm{mm^2}$. The total length of the LM channel inside the robot is 131 mm. The volume and the mass of the LM are 2.66 $\mathrm{mm^3}$ and 17.1 mg, respectively. The volume of the robot is $16\times9\times0.8 = 115.2\ \mathrm{mm^3}$ and the volume and the mass of the elastomer are 112.5 $\mathrm{mm^3}$ and 120.4 mg. The mass of the two feet is 3.2 mg (1.6 mg each). The mass of the two electrodes in the robot is 1.73 mg. The total mass of the robot as designed is about 142.4 mg (including the feet and electrodes inserted into the body).

Particularly, the mass of the real, SEMR TST tested in the experiments for vibrations is 179.7 mg. The difference is due to the fact that we cover the robot with an additional layer of the elastomer solution to insulate the electrodes. The increased mass of the elastomer is 37.3 mg and the corresponding thickness is about 0.24 mm if the additional elastomer is spread evenly on the surface of the SEMR (considering one big side of the body, an area of $16\times9\ \mathrm{mm^2}$). We measure the thickness of the robot at a $1/4$ body length and $1/2$ body length with a stylus profilometer (DektakXT, Bruker). The distribution of the thickness is not homogenous. The robot is thicker in the center and thinner towards the edges (Supplementary Fig. 14c).

1.6. Effect of gravity

We conducted a simple simulation examining the effect of gravity. We assume the homogeneous thickness and rectangular cross-section of the SEMR with dimensions of 9 mm × 1 mm before applying the strain mismatch. The length of the bilayer film before bending is 16 mm. The initial thickness of the prestretched membrane is 350 μm (from the experimental measurement) with a prestretch $\lambda_{pre} = 1.3$. The simulated static deformation of the bilayer film has the same curvature as that in the experiment (Fig. 2c). In the subsequent theoretical model, we do not account for the effect of gravity. To justify this, here we built a static numerical model in which the robot hangs from its midpoint with its legs moving freely. The results show that with gravity added, the tip of the leg has only 2.8% larger horizontal displacement in the x-direction than without gravity (Supplementary Fig. 14d). Thus, the gravity only (slightly) shifts the equilibrium, while the robot movement is governed by the deviations from this equilibrium.

1.7. Design and fabrication of the 3D printed substrate

We designed a sawtooth-shaped substrate, with a tooth cross-section of an isosceles right triangle (Supplementary Fig. 18), in order to force the SEMR to walk/run in one direction only. The free-body diagram of the SEMR walking on the sawtooth-shape substrate is shown in Supplementary Fig. 19. When the SEMR expands, its right leg moves along the diagonal side of the sawtooth but its left leg is blocked by the vertical face of the sawtooth. The situation is reversed when the SEMR contracts. When the current direction alternates periodically, the SEMR expands and contracts accordingly and walks/runs forward.

As the length of the sawtooth used for the foot is about 1.38 mm (Supplementary Fig. 13g), the height of the sawteeth in the substrate should be less than 1.38 mm. We tested five different heights for the sawteeth, a = 0.6 mm to a = 1 mm with a step size of 0.1 mm. The substrate for the running robot is fabricated by a 3D FDM printer (3 Extended, Ultimaker) with a 0.4 mm diameter nozzle. The printed substrate is not exactly the same as designed, because the minimal width of the printed line is 0.4 mm, and the minimal printed layer thickness is 0.06 mm. For example, for the substrate with a = 0.8 mm, the sawtooth is not triangular but trapezoid (Supplementary Fig. 18). We found that when the substrate sawtooth is too high (a = 0.9 mm and a = 1 mm), the robot gets stuck. When the sawtooth gets smaller (a = 0.6 mm and a = 0.7 mm), the robot slips. We further tested the running speed of the robot on two different substrates (a = 0.7 mm and a = 0.8 mm, Supplementary Fig. 20a), and the maximum running speed (129 mm/s) is reached for the substrate with a = 0.8 mm, this is about 31 mm/s faster than for the substrate with a = 0.7 mm (98 mm/s). Thus, the substrate with a = 0.8 mm sawtooth is the best and it was used for all walking and running tests henceforth.

1.8. Design and fabrication of SEMRs

1.8.1. Performance of the 3D LM printer

The FDM printer has a resolution of 0.01 mm, 0.01 mm, and 0.1 mm in x-, y- and z-direction, respectively. Our 3D printing system allows the printing of all kinds of patterns on elastomer films (Supplementary Fig. 3a). The printing speed of the printer is kept constant at 1 mm/s. The cross-section of the LM channels is close to a semicircular segment (Supplementary Fig. 3b) with width 217±20 μm, height 109±10 μm, and area 0.0203±0.003 mm^2 (Supplementary Figs. 3c&d). The resistance of the LM trace per length is 0.0142 Ω/mm. A view of the tip of the printhead is shown in (Supplementary Fig. 3e). It is ring-shaped with an inner diameter of 410 μm and an outer diameter of about 800 μm. This means that the tip may contact the previous liquid trace if the spacing between the adjacent traces (measured from the center of each trace) is less than 400 μm. We tested different distances between the centerlines of two LM traces, ranging from 400 μm to 250 μm with a step size of 50 μm, and found that the minimum distance between two printed traces can be about 300 μm (Supplementary Fig. 3f). In the following design, we set this distance to 500 μm with this tip, to decrease the fabrication failure rate.

A view of the small tip of the printhead is shown in Supplementary Fig. 3g. It is ring-shaped with an inner diameter of 200 μm and an outer diameter of about 400 μm. Based on a few tests, we set the distance between two printed traces to be 300 μm, which yields good results (Supplementary Fig. 3h) with an LM trace width of about 117±20 μm.

1.8.2. Feet design

We designed three types of feet for our robots: sawtooth-shaped, rectangular and L-shaped feet. The Sawtooth-shaped and rectangular ones are made of Kapton film and the L-shaped one is made of 3D printed plastic.

The maximum speed of the robot is feet-dependent. For the sawtooth-shaped feet, the angle of the feet to the 3D printed substrate is important. If the tilt angle of the feet (to the surface) is close to 90°, then the feet may get stuck in the groove of the substrate. If the tilt angle is close to 0°, then the feet may not insert into the grooves. As the angle of the feet is manually controlled and is difficult to adjust precisely, we suggest a tilt angle in the range of 30°-45° (see, for example, Supplementary Fig. 13f). The rectangular feet are similar to the sawtooth-shaped ones, but provide better support for large and heavy SEMRs. Instead of modifying the

geometries of the sawtooth-shaped and rectangular feet, we optimize the 3D printed substrate. This is shown in Supplementary Fig. 18 and Supplementary Fig. 20a.

The L-shaped feet are shown in Supplementary Fig. 24. Because the theory of locomotion for the SEMR with the L-shaped feet includes a complex dynamic mechanism, we optimized the geometry of these feet experimentally. The main parameters are La and Lb (Supplementary Fig. 24a). An increase in length La or Lb increases the mass of the feet and decreases the maximum speed. We first conduct a rough experiment to determine a range of values for La and Lb, which allow SEMR to run. The results indicate that La > 2 mm decreases the maximum speed of the robot and Lb (> 1 mm causes it to flip during actuation. In the end, we choose six different feet, type A to type F (Supplementary Fig. 24a, Supplementary Table 3). Type E (La = 2 mm and Lb = 0.5) exhibits the best performance. Extensive optimization of the L-shaped feet may further improve performance.

1.8.3. SEMR TST/TL

We design the body of the SEMR as a semicircle with a diameter of 9 mm. This keeps all robot dimensions in the mm range. A prestretch of 1.3 was chosen for three reasons. First, it is within the applicability region of the simple theory (Fig. 2b). Second, such stretch induces a large enough tensile stress in the film, which guarantees a flat, evenly stretched film (prevents wrinkling) for subsequent LM printing. Third, too large stretches cause rupture of the elastomer, when the membrane is mounted onto the frame (Fig. 1a). The typical range of required pristine membrane thicknesses follows from the previous calculation (assuming identical layers before prestretch) as $t_{10} = 3r\varepsilon_{10} / 4 = 1.0125\ \mathrm{mm}$, for $r = 4.5\ \mathrm{mm}$, $\varepsilon_{10} = 0.3$. The final thickness of the bilayer film is about 1.89 mm.

To further lower the bending stiffness of the bilayer, we can use a thinner pre-stretched layer and a thicker unstretched one. From the experiment, we find that the thickness of the elastomer film covering the LM should be larger than 300 μm to avoid the collapse of the LM channels during deformation. If we take $t_{10} = 350\ \mu\mathrm{m}$ commonly used in this work, the desired curvature according to Eq. (9) requires $t_{20} = 1.12\ \mathrm{mm}$, resulting in the total thickness of the bilayer film, 1.43 mm.

To choose proper prestretches for the bilayer film, we conducted separate simulations. With small prestretch (for example, 1.1), wrinkles on the thin film (about 350 μm) become a problem in the LM printing step. When the prestretch is too large (above ~1.7), the radius of the curved bilayer becomes too small for the robot. Thus, the prestretches are varied from 1.1 to 1.7 with a step size of 0.05. The original thickness of the prestretched layer is about 350 μm and the total thickness of the bilayer beam is about 1 mm. The arc length is 6.8 mm, which corresponds to a quarter of a circle with a radius of 4.3 mm (realized for a prestretch of 1.3). The shapes of the bilayer film with prestretches 1.1, 1.3, 1.5, and 1.7 are shown in Supplementary Fig. 37a. Then we apply a horizontal force at one end of the bilayer film, while the other end is fixed and calculate the resulting horizontal displacement of the forced end. The radius of the bilayer film always decreases with prestretch. However, the deflection of the bilayer initially decreases, only to increase again for prestretches larger than about 1.3. There are two reasons for such a non-monotonous deflection behavior. First, with a larger radius of the bilayer film comes a larger bending moment, which is equal to the product of the force and the distance to the fixed end of the beam. Second, higher prestretch increases the inherent bending stiffness of the bilayer film. We also analyzed the bending of the films with different arc lengths. The results show that it is easier for longer bilayer films to bend.

A moving robot should keep balance on the surface during the actuation. With this in mind, we make the robot close to a semicircle. If the shape is closer to a full circle, the two feet of the robot easily come into contact during actuation, causing the robot to stumble. If the robot is too flat, the lateral deformation becomes small (Supplementary Figs. 37a&b). The final choice of prestretch is 1.3, resulting in a robot radius of about 4.5 mm (body length, 9 mm).

1.8.4. SEMR TSTS

The two-module SEMR (Fig. 4f, Supplementary Figs. 28a&b) uses the same fabrication process as SEMR TST and a different design of the LM circuit (Supplementary Fig. 28c). To allow the SEMR to walk

on rubber (Silver, THERABAND), we modified the robot feet (Supplementary Figs. 28a&b): only two teeth are left on the front foot, and all teeth on the rear foot are removed.

1.8.5. SEMR TRC

The body of the transport SEMR is the same as that of the untethered SEMR, which can carry a battery and a controller. The coils are specifically designed to match the electric requirements of the battery and controller. Details of the body and coil design are provided in the section 1.8.7. The geometries of the coils, body, and release actuator are shown in Supplementary Fig. 29a and the fully assembled transport SEMR is depicted in Fig. 4g and Supplementary Fig. 29b. The design and fabrication process of the curved release actuator is similar to that SEMR, except that the feet foil is not added. The release actuator has a total thickness of about 0.8 mm and the thickness of the prestretched film (prestretch, 1.3) is about 0.35 mm. The weights of the body and the release actuator are 1 g and 0.42 g, respectively.

The body and the release actuator are both driven with a square-wave current (0.5 A, 8 Hz) by the PWM control system. In the experiment, the body was driven for 2 s, first carrying the cargo (a small medical capsule, 0.28 g) with a speed of 10 mm/s and then stopping. 1 s later, the release actuator is triggered and remains active for 0.5 s, causing it to eject the load (Supplementary Movie 9). All these actuations are performed by a custom script controlling the PWM system. To stabilize the capsule on the SEMR and prevent it from falling off, low driving speed is used. Faster transportation can be enabled by using a stronger soft grip as the release actuator.

1.8.6. SEMR UL

The body design and feet of the SEMR UL are the same as those of SEMR TL. The difference is that the LM coil of SEMR UL is redesigned (Supplementary Fig. 33a) and printed with a small tip with an inner diameter of 200 um. The resistance of SEMR UL is about 7.6 Ω which matches the high resistance of the small battery (~ 5 Ω). From the voltage output of the controller (Supplementary Fig. 33d), it is known that the current is a positive square wave in the range from 0.24 A to 0.29 A (voltage is from 1.8 V to 2.2 V) with an effective value of 0.16 A. Because the capacity of the small battery is 12 mAh, the SEMR UL can run for 270 s according to calculations. Considering the internal resistances of the SEMR and the battery, it is known that about 46% of the power is dissipated in the battery right now. Thus, this duration can be increased by increasing the number of the coil turns of the SEMR UL (increasing its resistance) to decrease the effective current keeping the Lorentz force intact. However, a better LM printer, for example, a multiple-layer LM printer, is required for this purpose.

1.8.7. SEMR UR1

The controller used for the untethered operation is a custom, flexible PCB with a timer chip as an astable multivibrator outputting a square wave. Due to the strong magnetic field, we choose a non-magnetic lithium battery, which has an internal resistance of about $R_{\text{in}} = 2\,\Omega$ (Supplementary Fig. 34a), a maximum open-circuit voltage $V_{\text{open}} = 4.1$ and a capacity of 40 mAh. The minimum current through the SEMR has to exceed $I_{\min} = 0.5$ A to provide the Lorentz force sufficient to run, which means that the total resistance should be smaller than $R_r = V_{\text{open}} / I_{\min} - R_{\text{in}} = 6.2\,\Omega$. As the open-circuit voltage decreases with decreasing state of charge (SoC) of the battery, the resistance of the SEMR should be around 5 Ω. The resistance per length of the LM channel is 0.0142 Ω/mm, and therefore the length of the LM channel should be around 352 mm.

To enable such long channels, and because the weight of the battery with controller is (1 + 0.18) g is much heavier than the tethered SEMR (~0.18 g), we designed the untethered SEMR to be significantly larger, with a thicker body, to support the battery and controller. In the flat state (Supplementary Fig. 29a), the SEMR body has a dimension of 22 mm×28 mm and the length of the LM channel is about 365 mm. The structure of the untethered SEMR is the same as that of the tethered one. The body of the untethered SEMR consists of two layers, sandwiching the printed LM channels. The numerical simulations predict, that the SEMR bends to a half-circle for prestretch 1.3, if the initial thickness of each of the two films is equal to 0.6 mm. In this

case, upon contraction under 0.5 A current, the arc angle of one leg would exceed 90°, which may lead to a loss of balance of the running robot with the battery on top. To avoid that, in fabrication, we used a prestretched film that was thicker than the design guidelines above (~ 0.8 mm) with a prestretch of 1.2, resulting in a total thickness of 1.4 mm and a smaller arc angle. The weight of the SEMR body is about 1 g. The total weight of the untethered SEMR is 2.18 g. To avoid the collapse of the sawtooth-shaped feet by this excess weight, we replaced the feet with a uniform, unstructured, rectangular Kapton film. The fabricated SEMR body is shown in Supplementary Figs. 34b-d with an overall dimension of the curved structure 19 mm × 22 mm × 10 mm (length × width × height), or 20 mm × 22 mm × 9 mm without and with the battery, respectively.

To obtain the optimal driving signal frequency for the untethered SEMR, we conducted experiments in which the untethered SEMR (with the inactive battery) was driven by the external PWM control system with the wires attached. The frequency sweep was from 1 Hz to 100 Hz with a square-wave current of 0.5 A amplitude and a duty cycle of 50% (no bias, positive half-period only, resulting in robot body contraction). The optimal frequencies (resulting in the highest speeds) lie in the range of 1 Hz to 10 Hz. Therefore, we set the driving frequency to 5 Hz for the untethered SEMR design.

The resistance of the fabricated untethered SEMR is about 4.6 Ω measured in 4-wire mode with a multimeter (2110, Keithley). The voltage output of the controller during actuation is characterized by an oscilloscope (GDS-1104B, GW Instek). Supplementary Fig. 34g shows the square-wave signals with an amplitude of about 2.76 V and short spikes above the plateau after the front edge of each pulse. The frequency and duty cycle of the signal are about 8.5 Hz and 56%, respectively, the deviations from the intended design parameters are due to the unstable output voltage of the lithium battery. If the controller is powered with a regulated power supply, the output frequency, and duty cycle are 5 Hz and 50%. The maximum battery-supplied current for the SEMR in the untethered regime is around 0.69 A, resulting in a maximum running speed of 42 mm/s (2.1 BL/s) (Supplementary Fig. 34h and Supplementary Movie 10).

We estimated the resonant frequency for a larger untethered robot using the framework from the section "planar vibrations of a curved beam". The frequency is similar to that given by the relations $(21)_{2\text{-}4}$, but the true maximum of the resonant curve $\omega_{\max} = 2\pi f_{\max}$ was used, because for larger robots the eigenfrequencies are much smaller, while the damping γ stays about constant. As a result, the damping frequency shift (similar to Eq. $(31)_2$) is much more pronounced, and the system operates closer to a non-oscillatory overdamped regime. We used the geometrical parameters from above with a one-leg arc angle $\psi = 3\pi/8 = 67.5^{\circ}$ and unchanged effective shear modulus of $\mu = 80\,\text{kPa}$. The result strongly depends on the assumed mass of the robot. Even when the total mass with the battery $m = 2.18\,\text{g}$ is used, the frequencies are in the range $f_{\max} = 10$ to $15\,\text{Hz}$ for the clamped and free-free regimes, which is somewhat higher than the experimental values yielding maximum running velocity. The discrepancy can be due to a complex geometry with the large concentrated battery and controller mass and the influence of gravity, as well as possible changes in shear modulus, both real and effective.

Because the densities of the controller and battery are much higher than that of water, the untethered SEMR cannot float on the water surface without further modification. To enable flotation, we add a small piece of polystyrene (PS) foam to the untethered SEMR (Supplementary Figs. 34e-f and Supplementary Movie 10). Deionized water is used to avoid damaging the controller and the battery.

According to the voltage output (Supplementary Fig. 34g) and the resistance of SEMR UR1 (Supplementary Table 5), the effective current is 0.36 A. The capacity of the medium battery is 40 mAh, which can drive SEMR UR1 for 400 s.

1.8.8. SEMR UR2

The SEMR UR2 is based on the SEMR UR1. As the large controller can output both positive and negative voltages (Supplementary Fig. 35b), we change the prestretch of the elastomer film from 1.2 (SEMR UR1) to 1.3 to enable a semicircular body of SEMR UR2. The effective current of SEMR UR2 is about 0.66 A. The large battery has a capacity of 150 mAh, which can power running SEMR UR2 for 817 s.

As the weight of the SEMR UR2 is 4.73 g, which is 2 times more than that of SEMR UR1 (Supplementary Table 5), we did not quantify the running speed of SEMR UR2. Instead, we measured the swimming speed of SEMR UR2, 1.8 BL/s, which is higher than the 1.25 BL/s of the SEMR UR1. This high

speed comes from two factors: the positive and negative output of the controller and better magnet 2 with SEMR UR2 rather than magnet 1 used with SEMR UR1. Clearly, further optimization of SEMR, or stronger magnets will increase the speed even more.

1.9. Planar vibrations of a curved beam

We describe our robot within the framework of small planar vibrations of a (thin) homogeneous Euler-Bernoulli (EB) beam (rod), with constant initial curvature (simplified Kirchhoff-Clebsch theory). Rotary inertia, shear deformation, and axial extensibility are neglected. The dynamic equations are adapted from §293(a) in ref.[5], Eqs. (7.69-70) in ref.[6], Eq. (39) in ref.[7], Eq. (10) in ref.[8] and the references there, and are related to the problem 8 in §20 of ref.[9], which is static and cannot be applied directly. As our equations are linear, we use complex amplitudes in the usual way - the real parts or modulus are implied in the final results.

The notations are as follows: w - radial displacement *towards the beam center*, u - tangential displacement towards increasing arc angle φ, R - initial beam radius, $0<\varphi<\psi$ - polar arc angle, ψ is the overall arc angle, $0<s=\varphi R<L$ is the arc length, L is the overall arc length (unless specified, ψ and L refer to the *half* of the robot-beam), $Y=3\mu$ - Young's modulus (for elastomers, the shear modulus μ is often reported), $\rho_L=\rho bh$ - linear mass density, b,h - width and thickness of the beam (with rectangular cross-section), $I_z=bh^3/12$ is the second moment of beam area in "radial" direction (the product YI_z is called flexural rigidity), $\eta_{w,u}$ - effective normal and tangential viscosities, $q_{w,u}$ - radial and tangential linear force densities (usually absent), the subscripts t and φ designate the corresponding partial derivatives.

$$w\overset{1}{=}u_\varphi, \qquad [\rho_L w_{tt}+\eta_w w_t+\frac{YI_z}{R^4}(\partial_{\varphi\varphi}+1)^2 w-q_w]_\varphi\overset{2}{=}\rho_L u_{tt}+\eta_u u_t-q_u \qquad (11)$$

To emphasize the similarities with the straight EB beam and static formulas, the expression in the square brackets is written in terms of radial displacement w. This also clarifies our definition of (effective, linear) viscous damping for both displacement components. Henceforth we will assume, that:

$$\eta_w=\eta_u=\rho_L\gamma \qquad (12)$$

Here, $\gamma[\mathrm{s}^{-1}]$ is some phenomenological damping coefficient, deduced from the experimental resonance curves. The full analysis uses the equation for the tangential displacement u, substituted into Eq. $(11)_2$ from Eq. $(11)_1$, and requires the expressions for the (out of plane) torque M, shear force N, and tension T, which are used in the boundary conditions:

$$\begin{aligned} &M\overset{1}{=}\frac{YI_z}{R^2}(w_{\varphi\varphi}+w), \qquad N\overset{2}{=}-\frac{YI_z}{R^3}(w_{\varphi\varphi}+w)_\varphi \\ &T\overset{3}{=}R(\rho_L w_{tt}+\eta_w w_t)-N_\varphi\overset{4}{=}R(\rho_L w_{tt}+\eta_w w_t)+\frac{YI_z}{R^3}(w_{\varphi\varphi}+w)_{\varphi\varphi} \end{aligned} \qquad (13)$$

In the harmonic case, we look for the solutions in the form $w,u\sim e^{i\omega t}e^{k\varphi}$. Without distributed forces, $q_{w,u}=0$, Eq. (11) yields a characteristic equation for the dimensionless angular wavenumber k:

$$k^2(k^2+1)^2\overset{1}{=}Z^2(k^2-1), \qquad Z^2\overset{2}{=}(\omega^2-i\omega\gamma)\frac{\rho_L R^4}{YI_z} \qquad (14)$$

This 6th order equation has 6 (complex) solutions k_j, which depend on a single parameter Z^2. It is complex in the presence of damping and real positive if $\gamma=0$; in the static case $Z^2=0$. Because the Eq. (14) is bicubic, its roots are grouped into 3 pairs $\pm k_j$, and the explicit expressions are given in Eqs. (55)-(56). The spatial (angular) profile of both displacements is the combination of 6 terms:

$$\begin{aligned} &u\overset{1}{=}\sum_{j=1}^{6}u_j\overset{2}{=}\sum_{j=1}^{6}A_j e^{k_j\varphi}, \quad w\overset{3}{=}u_\varphi\overset{4}{=}\sum_{j=1}^{6}A_j k_j e^{k_j\varphi} \quad \Rightarrow \\ &u\overset{5}{=}\sum_{j=1}^{3}(S_j\sinh k_j\varphi+C_j\cosh k_j\varphi), \qquad w\overset{6}{=}\sum_{j=1}^{3}k_j(S_j\cosh k_j\varphi+C_j\sinh k_j\varphi) \end{aligned} \qquad (15)$$

In the final expressions (15)5,6 we regrouped the pairs $\pm k_j$ into hyperbolic functions, which are convenient for symmetric profiles. One can also use trigonometric functions, but for complex notations hyperbolic functions are easier, as their signs do not alternate upon differentiation.

Six constants S_j, C_j should be found from six boundary conditions at both ends. We assume that our system is (approximately) symmetric. In this case, it is more convenient to use $u = 0$ and even w-function condition in the middle, at $\varphi = 0$, together with the boundary conditions only at the right end $\varphi = \psi$. This works only for the symmetric driving force and implies that "odd" modes are never excited (which is not fully true for a moving robot). If radial and tangential forces $F_{w,u}$ are applied with appropriate symmetry at both ends $\varphi = \pm\psi, s = \pm L$ (without torque), one can use the following modified "boundary" conditions:

$$\begin{aligned} &\varphi = 0: \quad u \underset{\text{sym.}}{\overset{1}{=}} 0, \quad w_\varphi \underset{\text{sym.}}{\overset{2}{=}} 0, \quad w_{\varphi\varphi\varphi} \underset{\text{sym.}}{\overset{3}{=}} 0, \quad w \underset{\text{clamped-free}}{\overset{3a}{=}} 0 \\ &\varphi = \psi: \quad w_{\varphi\varphi} + w \underset{M}{\overset{4}{=}} 0, \quad w_{\varphi\varphi\varphi} + w_\varphi \underset{N}{\overset{5}{=}} -\frac{F_w R^3}{YI_z}, \quad w_{\varphi\varphi\varphi\varphi} + w_{\varphi\varphi} - Z^2 w \underset{T}{\overset{6}{=}} \frac{F_u R^3}{YI_z} \end{aligned} \tag{16}$$

In the case of a clamped-free beam, the conditions (16)3a, (17)3a should be used instead of Eqs. (16)3, (17)3. Substituting here the expressions (15)5,6, we obtain the linear system for six coefficients S_j, C_j.

$$\begin{aligned} &\sum_{j=1}^{3} C_j \underset{\text{sym.}}{\overset{1}{=}} 0, \quad \sum_{j=1}^{3} k_j^2 C_j \underset{\text{sym.}}{\overset{2}{=}} 0, \quad \sum_{j=1}^{3} k_j^4 C_j \underset{\text{sym.}}{\overset{3}{=}} 0, \quad \sum_{j=1}^{3} k_j S_j \underset{\text{clamped-free}}{\overset{3a}{=}} 0 \\ &\sum_{j=1}^{3} k_j (k_j^2 + 1)(S_j \cosh k_j\psi + C_j \sinh k_j\psi) \underset{M}{\overset{4}{=}} 0 \\ &\sum_{j=1}^{3} k_j^2 (k_j^2 + 1)(S_j \sinh k_j\psi + C_j \cosh k_j\psi) \underset{N}{\overset{5}{=}} -\frac{F_w R^3}{YI_z} \overset{5a}{=} f \sin\psi \\ &\sum_{j=1}^{3} k_j [k_j^2 (k_j^2 + 1) - Z^2](S_j \cosh k_j\psi + C_j \sinh k_j\psi) \underset{T}{\overset{6}{=}} \frac{F_u R^3}{YI_z} \overset{6a}{=} f \cos\psi \end{aligned} \tag{17}$$

In Eqs. (17)5a,6a, we introduced a parameter combination f, which can be expressed in several equivalent forms, using relations (21) for the eigenfrequency:

$$f \overset{1}{=} \frac{FR^3}{YI_z} \overset{2}{=} \frac{FL^3}{\psi^3 YI_z} \overset{3}{=} \frac{FZ_0^2}{\rho_L R\omega_0^2} \overset{4}{=} \frac{F\psi Z_0^2}{\rho_L L\omega_0^2} \tag{18}$$

The last form emphasizes the similarity with the Lorentzian oscillator (23)-(24). We further assumed in Eqs. (17)5a,6a, that the magnetic driving force F is horizontal and applied at the beam end(s). Its radial (towards the center) and tangential components are:

$$F_w \overset{1}{=} -F \sin\psi, \quad F_u \overset{2}{=} F \cos\psi \tag{19}$$

Linear systems (17) can be solved with computer algebra software like Mathematica, or numerically. The determinant of the underlying matrix enters the denominator of all expressions:

$$\det m_{ij} = D(k_j(Z(\omega))) = D(\omega) \tag{20}$$

In the absence of damping (when $Z^2 = \mathrm{Re} > 0$, as defined by Eq. (14)2) $D(\omega_n) = 0$ determines the eigenfrequencies, including the lowest one, ω_0. The dimensionless roots $Z_n(\psi, \text{b.c.})$ depend on the arc length ψ and the *type* of the boundary conditions, which modify the matrix m_{ij} and its determinant (20) (see Eqs. (17)). The resonant frequency for the root $Z_0(\psi)$ follows from definition (14)2:

$$\begin{aligned} &\omega_0 \overset{1}{=} \frac{Z_0}{R^2}\sqrt{\frac{YI_z}{\rho_L}} \overset{2}{=} \frac{\Omega_\psi}{L^2}\sqrt{\frac{YI_z}{\rho_L}} \overset{3}{=} \frac{\Omega_\psi h}{2L^2}\sqrt{\frac{\mu}{\rho}}, \qquad \Omega_\psi \overset{4}{=} \psi^2 Z_0(\psi) \\ &YI_z \overset{5}{=} \frac{\rho_L R^4 \omega_0^2}{Z_0^2} \overset{6}{=} \frac{\rho_L L^4 \omega_0^2}{\Omega_\psi^2} \end{aligned} \tag{21}$$

The expression $(21)_2$ is written in terms of beam length L, for comparison with the straight case and between the different curvatures, while Eq. $(21)_3$ uses geometrical and material parameters. The expressions $(21)_{5,6}$ express flexural rigidity via the resonant frequency. The dimensionless factor $\Omega_\psi = \psi^2 Z_0(\psi)$ for representative geometries is listed in Supplementary Table 7 (referring to half-beam arc angle ψ).

With damping $\gamma \neq 0$, both Z in Eq. $(14)_2$, and the denominator D become complex and $D(Z(\omega)) \neq 0$ everywhere. Near the resonance D is small, and the response is big. Taylor expansion of Eq. $(14)_2$ near the resonance yields:

$$Z^2 \approx Z_0^2(1 - i\gamma / \omega_0) \tag{22}$$

From here it is clear that the resonant amplitudes are proportional to ω_0 / γ.

1.10. Oscillator approximation and velocity

Once the radial and tangential displacements w, u are found, the horizontal and vertical displacements x, y follow from simple geometrical considerations, see Eq. (60). We are interested in their end values, $x(\psi, \omega), y(\psi, \omega)$, given by the Eqs. (61). The spectral response of all quantities is very similar to that of a simple forced harmonic oscillator.

$$\ddot{x} + \gamma \dot{x} + \omega_0^2 x = \frac{F}{m} e^{i\omega t} \tag{23}$$

The (stationary, complex) amplitude of such an oscillator is given by the Lorentz curve:

$$X(\omega) \overset{1}{=} \frac{F/m}{\omega_0^2 - \omega^2 + i\omega\gamma}, \qquad X(0) \overset{2}{=} \frac{F}{m\omega_0^2}, \quad X(\omega_0) \overset{3}{=} \frac{-iF}{m\gamma\omega_0}, \qquad \left| \frac{X(\omega_0)}{X(0)} \right| \overset{4}{=} \frac{\omega_0}{\gamma} \tag{24}$$

Near the resonance, for small damping, the denominator in Eq. $(24)_1$ can be approximated as $2\omega_0(\omega_0 - \omega + i\gamma/2)$, therefore γ is the full width at half-maximum of the Lorentzian power spectrum (square of the amplitude $(24)_1$). The transient processes occur on the timescale $t \sim \gamma^{-1}$ and can be easily analyzed. For small damping, $\gamma / \omega_0 \ll 1$ the amplitude always almost doubles at first, before it stabilizes. For brevity, we do not discuss here (well-known) transient and near-resonant subtleties.

To utilize the oscillator analogy (23)-(24), we characterize the spectral response of the beam $x(\psi, \omega)$ by its eigenfrequency ω_0, damping γ, and the (effective) mass $m_{\text{eff},x} = m_x \rho_L L$ (similarly for y). This mass defines the *static* deflection magnitude $x(\psi, 0)$ in analogy to Eq. $(24)_2$:

$$x(\psi, 0) \overset{1}{=} \frac{F}{m_{\text{eff},x}\omega_0^2} \overset{2}{=} \frac{F}{m_x \rho_L L \omega_0^2} \overset{3}{=} \frac{FL^3}{m_x \Omega_\psi^2 Y I_z} \overset{4}{=} \frac{4}{m_x \Omega_\psi^2} \frac{FL^3}{\mu b h^3} \tag{25}$$

The dimensionless mass factors $m_{x,y}$ are listed in Supplementary Table 7. In analogy to Eq. $(24)_3$, the maximal amplitude can be written as follows (the phase is also similar to the oscillator case):

$$x(\psi, \omega_0) \overset{1}{=} \frac{C_x F}{m_x \rho_L L \gamma \omega_0} \overset{2}{=} \frac{C_x F L}{m_x \Omega_\psi \gamma \sqrt{\rho_L Y I_z}} \overset{3}{=} \frac{2C_x}{\Omega_\psi m_x} \frac{FL}{\gamma b h^2 \sqrt{\mu \rho}} \tag{26}$$

Representative factors C_x are listed in Supplementary Table 7, they are always close to 1. For the sake of generality, we omit them in the Lorentzian approximations. Once the parameters of the resonant curve are found, theoretically, numerically, or experimentally, further analysis can use them, together with the simple oscillator results (23)-(24).

The velocity can be estimated as the quadrupled one-leg amplitude, multiplied by the (temporal) frequency $f = \omega / 2\pi$. The coefficient 4 comes from the difference between the fully contracted and fully extended two-leg robot. The maximum velocity value can be estimated as:

$$v(\omega) \overset{1}{=} \underset{\omega/\pi}{4f}\, x(\psi, \omega), \qquad v_{\max} \overset{2}{=} 4 f_0 x(\psi, \omega_0) \overset{3}{=} \frac{2C_x F}{\pi m_x \rho_L L \gamma} \overset{4}{=} \frac{2C_x}{\pi m_x} \frac{F}{m\gamma} \tag{27}$$

This velocity is inversely proportional to the overall mass of the robot, and the (effective) damping γ. Relation $(27)_1$ is an estimation from above, the real speed may be a fraction of this value. Its exact value depends on

the assumptions about the damping mechanisms and surface friction during the slippage of the feet along the surface.

General measure of damping rate is the ratio of dissipated power to the overall energy of the system. For the harmonic oscillator, this leads to the Eqs. $(24)_4$ and therefore to the Eq. $(27)_{3,4}$, but this rate is similar also for more complex robot movement. As an illustration, let us consider two equal masses m on a harmonic spring (modeling the legs of our robot), with the center of mass (COM) moving with the constant velocity v_{COM}, and oscillating with the relative velocities v_{rel}.

Let us first estimate the overall energy. The translational kinetic energy of the COM is $2mv_{\text{COM}}^2/2 = mv_{\text{COM}}^2$. In the moving frame, the *average* kinetic energy of each mass is $mv_{\text{rel}}^2/4$. The average potential (elastic) energy for a harmonic oscillator equals kinetic one, so that the overall oscillatory energy is $2\times 2\times mv_{\text{rel}}^2/4 = mv_{\text{rel}}^2$. The total energy is the sum of this value and the COM kinetic contribution (König's theorem), $E_{\text{tot}} = mv_{\text{COM}}^2 + mv_{\text{rel}}^2$.

Let us now estimate the dissipated power within such a moving oscillator analogy for two idealized cases. If the damping is purely internal (elastic) and due to vibrational motion only, it can be found in the inertial COM frame, where the time averaged dissipation for each mass is $<\gamma mv_{\text{rel}}^2>=\gamma mv_{\text{rel}}^2/2$, so that the total dissipated power is twice this value, $P_{\text{osc}} = \gamma mv_{\text{rel}}^2$.

In the contrasting model case the damping force $-m\gamma v$ is proportional to the velocity of each mass in the laboratory frame, $v = v_{\text{COM}} \pm v_{\text{rel}}\cos\omega t$, for example due to (viscous) friction with the floor, or air. The dissipated power is the product of force and velocity, and its average value for each mass is $<\gamma m(v_{\text{COM}} \pm v_{\text{rel}}\cos\omega t)^2>=\gamma m(v_{\text{COM}}^2 + v_{\text{rel}}^2/2)$. The total dissipated power $P_{\text{abs}} = \gamma m(2v_{\text{COM}}^2 + v_{\text{rel}}^2)$ becomes higher here, because the absolute velocities v are larger than the relative ones.

If we introduce the ratio of COM and vibrational energies, $C = v_{\text{COM}}^2/v_{\text{rel}}^2$, the ratios of the dissipated power and the total energy for these 2 cases become:

$$\frac{P_{\text{osc}}}{E_{\text{tot}}} \overset{1}{=} \frac{\gamma}{1+C}, \quad \frac{P_{\text{abs}}}{E_{\text{tot}}} \overset{2}{=} \gamma\left(1+\frac{C}{1+C}\right) \tag{28}$$

The velocities $v_{\text{COM}} \sim v_{\text{rel}}$ are usually comparable; for example, if $v_{\text{COM}} \ge v_{\text{rel}} \ge 0$, the velocities of both feet remain positive, and there is no "backwards slippage", however this is not necessarily the case in general. Although the damping force may not be linear in velocity, and $v_{\text{COM}} \ne \text{const.}$, the estimations (28) illustrate, that the energy dissipation rate remains comparable with the (phenomenological) damping coefficient γ, irrespectively of its underlying physical mechanism, see also the section "Estimations of the damping coefficient" below.

1.11. Square-wave (SW) excitation

The Fourier series of an odd unit square-wave (SW) (adapted from ref.[10]) is given by:

$$\text{sw}(t) \overset{1}{=} \text{sgn}(\sin\omega t) \overset{2}{=} \frac{4}{\pi}\sum_{n=0}^{\infty}\frac{\sin((2n+1)\omega t)}{2n+1} \overset{3}{=} \text{Im}\frac{4}{\pi}\sum_{n=0}^{\infty}\frac{e^{i(2n+1)\omega t}}{2n+1} \tag{29}$$

It contains only odd harmonics and the amplitude contribution from the base frequency is $4/\pi \approx 1.27$. Near a narrow resonance (neglecting higher harmonics contribution) the amplitude response will be larger than for a sinusoidal wave of the same amplitude. If SW has the same *AC power* (effective current), as the sinusoidal, the coefficient in Eq. (29) becomes $2\sqrt{2}/\pi \approx 0.9$ and the resonant response is smaller. The exact spectral amplitude of the response is not trivial, due to the phases of the harmonics. If the complex spectral response to a (unit) sinusoidal is $x(\omega)$, the response to a (unit) SW can be written emphasizing the base frequency:

$$x_{\text{sw}}(t) \overset{1}{=} \text{Im}\, e^{i\omega t}\frac{4}{\pi}\sum_{n=0}^{\infty}\frac{x((2n+1)\omega)}{2n+1}e^{2in\omega t} \overset{2}{\le} \frac{4}{\pi}\sum_{n=0}^{\infty}\frac{|x((2n+1)\omega)|}{2n+1} \tag{30}$$

The slow amplitude in $(30)_1$ is periodic, but the non-constant function of t, its shape and maximum depend on the phase relations between $x(\omega)$ and the phase ωt within the period. For example, when $\omega t = 0$, $e^{2in\omega t} = 1$, and all complex amplitudes added in phase, while at quarter period $\omega t = \pi / 2$, $e^{2in\omega t} = (-1)^n$, and the amplitudes have alternating signs. For a Lorentzian oscillator $(24)_1$ the phase $x(\omega_0)$ is $-\pi / 2$ at resonance, but close to $-\pi$ for higher harmonics $n\omega_0$, and the amplitudes are added accordingly. The sum of the absolute values $(30)_2$ provides an obvious upper limit to the SW response amplitude (Supplementary Fig. 42c, or Supplementary Fig. 43a, purple curves $\sum |x_i|$), but for the oscillating systems the Lorentzian estimations (37), (43) are more accurate and instructive.

For the Lorentzian oscillator, the situation can be analyzed in the time domain. It is convenient to use mixed real-complex representation. The system switches between free oscillations around two equilibria $x_{\text{eq}} = \pm\omega_0^{-2}$ (henceforth, using linearity, we set $F / m = 1$ for brevity and reintroduce this factor in the final formulas (37) and the text after it). These equilibria are approached in free damped oscillations with the exponent λ, in which we will use the "+" sign and disregard the overdamped case $\gamma > 2\omega_0$:

$$\begin{aligned} &\lambda \overset{1}{=} -\gamma / 2 + i\omega_1, \qquad \omega_1 \overset{2}{=} \sqrt{\omega_0^2 - \gamma^2 / 4} \\ &|\lambda| \overset{3}{=} \omega_0, \qquad \arg\lambda \overset{4}{=} \arctan\frac{\omega_1}{-\gamma / 2} \overset{5}{\approx} -\frac{\pi}{2} + \delta \end{aligned} \tag{31}$$

Let the period of the driving force be

$$2\tau = 2\pi / \omega \tag{32}$$

We write the solution for the positive half of the period in the complex form. As SW is conveniently represented in odd form (29), we use the imaginary, rather than the real part.

$$x(0 < t < \tau) \overset{1}{=} \omega_0^{-2} + \operatorname{Im} Ae^{\lambda t} \overset{2}{=} \omega_0^{-2} + |A| e^{-\gamma t/2} \sin(\omega_1 t + \varphi), \qquad \varphi \overset{3}{=} \arg A \tag{33}$$

Due to symmetry and periodic translation (in stationary periodic regime), for the negative part of the period the solution is:

$$x(-\tau < t < 0) \overset{1}{=} -x(t + t_0) \overset{2}{=} -\omega_0^{-2} - \operatorname{Im} Ae^{\lambda(t+\tau)} \tag{34}$$

The continuation for other t is periodic. The (real) functions (33)-(34) and their derivatives should match at $t = 0$. After a simple transformation, one obtains:

$$\operatorname{Im} A(1 + e^{\lambda\tau}) \overset{1}{=} -2\omega_0^{-2}, \qquad \operatorname{Im} \lambda A(1 + e^{\lambda\tau}) \overset{2}{=} 0 \tag{35}$$

We denote the combination $A(1 + e^{\lambda\tau})$ by a complex number z, substitute it into Eqs. (35), separate real and imaginary parts and make some transformations with the help of Eqs. (31):

$$\begin{aligned} &A(1 + e^{\lambda\tau}) \overset{1}{=} z \overset{2}{=} z' + iz'', \qquad \operatorname{Im} z \overset{3}{=} -2\omega_0^{-2}, \;\; \operatorname{Im} \lambda z \overset{4}{=} 0 \qquad \Rightarrow \\ &z \overset{5}{=} -\omega_0^{-2}\left(\frac{\gamma}{\omega_1} + 2i\right) \overset{6}{=} \frac{2}{\lambda \operatorname{Im}\lambda}, \qquad A \overset{7}{=} \frac{z}{1 + e^{\lambda\tau}} \overset{8}{=} \frac{2}{(1 + e^{\lambda\tau})\lambda \operatorname{Im}\lambda} \end{aligned} \tag{36}$$

Finally, substituting λ and τ from Eqs. (31)-(32), and restoring the proportionality coefficient F / m, we obtain the complex spectral response amplitude A of the Lorentzian oscillator Eqs. (23)-(24) to the SW:

$$A = \frac{2F / m}{\lambda\omega_1(1 + e^{\lambda\tau})}, \qquad \lambda\tau = (-\gamma / 2 + i\omega_1)\frac{\pi}{\omega} \tag{37}$$

Complex amplitude A contains a resonant denominator, which becomes small when $e^{i\pi\omega_1/\omega} = -1$. For small damping, at resonance one obtains:

$$\omega_l \overset{1}{=} \frac{\omega_1}{2l + 1}, \qquad 1 + e^{\lambda\tau} \overset{2}{\approx} \frac{\gamma\tau}{2} \overset{3}{=} \frac{\pi(2l + 1)\gamma}{2\omega_0}, \qquad A_l \overset{4}{\approx} \frac{-4iF}{\pi(2l + 1)m\omega_0\gamma} \tag{38}$$

These expressions can be compared with the resonant amplitude $(24)_3$ for the sinusoidal wave. The resonances occur for the odd fractions of the natural frequency. The phases are the same, and the amplitudes decrease proportionally to the odd divisor. The factor $4 / \pi$ comes from the Fourier coefficients of the unit SW (29).

Because the oscillations occur around the equilibrium positions $\pm F/m\omega_0^2$, the maximum *deflection* $x_{\max}$ somewhat differs from the amplitude $|A|$ in Eq. (37). It can be found using complex representation (33). Zero derivative condition yields, with the help of Eq. (36)8 (omitting F/m factor for brevity):

$$\dot{x} \overset{1}{=} \operatorname{Im}\lambda A e^{\lambda t} \overset{2}{=} \operatorname{Im}\frac{2e^{\lambda t}}{(1+e^{\lambda\tau})\operatorname{Im}\lambda} \overset{3}{=} 0 \quad \Rightarrow \quad \arg e^{\lambda t} \overset{4}{=} \omega_1 t \overset{5}{=} \pi n + \arg(1+e^{\lambda\tau})$$
$$\arg A \overset{6}{=} -\arg\lambda - \arg(1+e^{\lambda\tau}) \quad \Rightarrow \quad \arg A e^{\lambda t} \overset{7}{=} \pi n - \arg\lambda \tag{39}$$

We use the combination (39)7 for the value at extrema:

$$x_{\text{ext}} \overset{1}{=} \omega_0^{-2} + \operatorname{Im} A e^{\lambda t} \overset{2}{=} \omega_0^{-2} + \left|Ae^{\lambda t}\right| \sin(\arg A e^{\lambda t}) \overset{3}{=} \omega_0^{-2} - |A| e^{-\gamma t/2}(-1)^n \sin(\arg\lambda) \overset{4}{=}$$
$$\omega_0^{-2} - (-1)^n |A| e^{-\gamma t/2}\frac{\omega_1}{\omega_0} \Rightarrow \quad |x_{\text{ext}}|_{\max} \overset{5}{=} \frac{\operatorname{sign}(\omega_1-\omega)}{\omega_0^2} + |A|\frac{\omega_1}{\omega_0}e^{-\gamma t/2} \tag{40}$$

In Eqs. (40)3,4 we used that λ lies in the 2nd quadrant, see the definitions (31)4,5. This implies, that the maxima n must be odd. For the first (largest) maximum, the smallest possible n should be chosen, which provides $t > 0$. Thich results in $n = 1$, and we find the time from the Eq. (39)5 (1st equality below):

$$t_{\max} \overset{1}{=} \frac{\pi + \arg(1+e^{\lambda\tau})}{\omega_1} \overset{2}{\mapsto} \frac{\arg(\omega-\omega_1) + \arg(1+e^{\lambda\tau})}{\omega_1} \tag{41}$$

However, for short excitation periods (large $\omega > \omega_1$) the maximum does not occur within the considered half period $0 < t < \tau$. This happens if

$$t_{\max} \overset{1}{>} \tau \overset{2}{=} \frac{\pi}{\omega} \quad \Rightarrow \quad \frac{\pi + \arg(1+e^{\lambda\tau})}{\omega_1} \overset{3}{>} \frac{\pi}{\omega} \quad \Rightarrow \quad \overset{4}{\omega > \omega_1} \tag{42}$$

One can show, that the transition occurs exactly at $\omega > \omega_1$, where $\arg(1+e^{\lambda\tau}) = 0$. In this case, the maximum deflection is reached at the 1st minimum, with $n = 0$ and "-" sign in Eq. (40)4, and the sign of the result should be inverted. The combined result can be written via single expressions (42)2 and (40)5. Restoring the common pre-factor F/m one obtains the final compact result.

$$x_{\max} \overset{1}{=} \frac{F}{m}\left(\frac{\operatorname{sign}(\omega_1-\omega)}{\omega_0^2} + |A|\frac{\omega_1}{\omega_0}e^{-\gamma t_{\max}/2}\right) \overset{2}{=} \frac{F}{m\omega_0^2}\left(\operatorname{sign}(\omega_1-\omega) + \frac{2e^{-\gamma t_{\max}/2}}{\left|1+e^{\lambda\tau}\right|}\right) \tag{43}$$

This expression appropriately tends to 0 for $\omega \gg \omega_1$, where the response to an SW is almost sinusoidal, while the amplitude $|A|$ remains finite and does not yield the correct result. It has somewhat different behavior than $|A|$ (compare the yellow x_{Lorsw} and olive A_{Lorsw} curves in Supplementary Fig. 42c, or yellow x_{maxsw} and red A_{sw} curves in Supplementary Fig. 43a), although for small damping they are close at moderate frequencies, especially at resonances, where the Eq. (38)3 can be used and the leading term coincides with the Eq. (38)4.

In natural units of $F/m\omega_0^2$, the static deflection for $\omega \to 0$ and small damping, is 2 for $|A|$ and 3 for $x_{\max}$ rather than 1 for sinusoidal excitation, for a clear physical reason - the overshot in the first oscillation swings upon change to the new equilibrium (Supplementary Fig. 44).

The derivation of the robot velocity (27) requires generalization for the SW excitation. When one leg moves in one direction, another moves in the opposite. We assume ideal asymmetric friction, which implies that the total robot displacement is the sum of the absolute differences between all adjacent extrema of the one-leg oscillations. Due to symmetry (34), $x_{\max}(t) = -x_{\min}(t+\tau)$ and vice versa, and we can double the summation over half a period:

$$\Delta x \overset{1}{=} \sum_{2\tau}|x_n - x_{n+1}| \overset{2}{=} 2\sum_{\tau}|x_n - x_{n+1}| \overset{3}{=} 4\left(\sum_{\tau}x_{\max} - \sum_{\tau}x_{\min}\right) \tag{44}$$

Even when there is only one extremum per half-period τ, for symmetry reasons, the Eq. (44)3 yields the correct result. The velocity is then calculated by dividing it by the full period 2τ:

$$v_{\mathrm{sw}} \overset{1}{=} \frac{\Delta x}{2\tau} \overset{2}{=} 4f\left(\sum_{\tau} x_{\max} - \sum_{\tau} x_{\min}\right) \qquad (45)$$

Similar to the discussions below Eq. (27), the coefficient 4 in Eq. $(45)_2$ represents an upper bound. The extrema times are given by the condition $(39)_5$ and should fulfill the requirement $0 < t_n < \tau$. This implies the following statements about the numbers n in terms of various rounding, modulo, and congruence functions (in Mathematica notations).

$$\overset{1}{\alpha} = \arg(1+e^{\lambda\tau}), \qquad t_n \overset{2}{=} \frac{\pi n + \alpha}{\omega_1}$$

$$n_{\min} \overset{3}{=} \left\lceil -\frac{\alpha}{\pi} \right\rceil \overset{4}{=} \left\lfloor \mathrm{mod}\left(\frac{\omega_1}{\omega}, 2\right) \right\rfloor, \quad n_{\max} \overset{5}{=} \left\lceil \frac{\omega_1}{\omega} - \frac{\alpha}{\pi} \right\rceil - 1 \overset{6}{=} \left\lfloor \frac{\omega_1}{\omega} \right\rfloor \qquad (46)$$

$$n_{\max} - n_{\min} + 1 \overset{7}{=} \left\lfloor \frac{\omega_1}{\omega}, 2 \right\rfloor + 1, \qquad -(-1)^{n_{\min}} \overset{8}{=} -(-1)^{n_{\max}} \overset{9}{=} (-1)^{\left\lceil \frac{\omega_1}{\omega} \right\rceil}$$

Extrema values are given by $(40)_4$, and for topological and symmetry reasons the number of extrema per half-period is always odd and given by Eq. $(46)_7$. If $n_{\min} = 0$, it starts and ends with a minimum, and the constant term survives with the sign "-". For $n_{\min} = 1$, the constant term survives with the sign "+". This can be combined into a single expression $(46)_9$. The sign-alternating exponential terms from $(40)_4$ always add positively in $(45)_2$ and can be summed as a geometrical progression, which is equivalent to the averaging of the amplitudes.

$$\sum_{n=n_{\min}}^{n_{\max}} e^{-\gamma t_n/2} = \frac{e^{-\gamma t_{\min}/2} - e^{-\gamma t_{\max}/2} e^{-\pi\gamma/2\omega_1}}{1 - e^{-\pi\gamma/2\omega_1}} \qquad (47)$$

Adding the necessary pre-factors, all this can be combined into a single formula for the velocity.

$$v_{\mathrm{sw}} = \frac{2\omega}{\pi} \frac{F}{m\omega_0^2} \left((-1)^{n_{\max}+1} + \frac{2}{\left|1+e^{\lambda\tau}\right|} \frac{e^{-\gamma t_{\min}/2} - e^{-\gamma t_{\max}/2} e^{-\pi\gamma/2\omega_1}}{1 - e^{-\pi\gamma/2\omega_1}} \right) \qquad (48)$$

This expression can be written in various equivalent forms using relations (46). Similarly to $(27)_1$, the dependence (48) has the common pre-factor ω. This curve is shown in Supplementary Fig. 42a, d by the yellow v_{Lorsw} curves, as well as by the red v_{sw} curve in Supplementary Fig. 43b. The response differs appreciably from the sinusoidal velocity (27) at low frequencies, where the SW response has secondary resonances.

1.12. Speed estimations for a hovering robot

For high currents at near-resonant frequencies, where the speed is high, the robot hovers in the air most of the time (see the lower panel in Fig. 3e and Supplementary Movie 3). In this case, our explanation of the movement mechanism needs updating. Let us consider the "flying" robot, which only "from time to time", possibly even not in each vibration, pushes off the substrate with one of the legs. The push will be in the forward direction, only if the absolute velocity of the robot foot points backwards. This implies, that the robot center of mass velocity v cannot exceed the relative edge velocity of the vibrating legs: $v < v_{\mathrm{vib}}$. For the sinusoidal vibrations with the amplitude $x(\omega)$ this immediately produces the estimation:

$$v < \omega x(\omega) = 2\pi f x(\omega) \qquad (49)$$

This is similar to the expression $(27)_1$, only with the coefficient 2π instead of 4, which is not surprising for an "upper end" estimation. The numbers somewhat change for the SW excitation, but the main argument persists.

When the robot is in the air, the magnetic forces in both legs cancel in the overall force balance. During the "push", one of them is (partially) canceled by the horizontal reaction force from the substrate (groove). Thus, the overall (horizontal) force acting on the robot is not larger than the forward magnetic force in one of the legs, $F(0.2\mathrm{A}) \approx 1.08\,\mathrm{mN}$, and $F(0.5\mathrm{A}) \approx 2.7\,\mathrm{mN}$ (these are full force values, see also the experimental Supplementary Fig. 15d). Acting continuously, such a force (which is constant for an SW excitation), would

produce a forward acceleration $a_x = F/m$, where m is the full robot mass. $a_x(0.2\text{A}) = 6010\,\text{mm/s}^2$, $a_x(0.5\text{A}) = 15025\,\text{mm/s}^2$. The parabolic dependence for $I = 0.5\,\text{A}$ in Supplementary Fig. 21d yields about 4 times lower number $a_x(0.5\text{A}) = 3990\,\text{mm/s}^2$. This can be understood upon visual inspection of the Supplementary Movie 3. Due to inherent irregularities, the robot practically always touches the ground with one leg only, and about half of the times such a contact happens in "anti-phase", leading to the slippage due to asymmetric friction, instead of the "helpful push off". These two general reasons already reduce the expected acceleration by a factor of 4. Another factor of 2 is mainly due to a combination of the following reasons: First, a touch occurs not in every vibration; one can clearly observe full swings in the "flying state". Second, although the excitation and the magnetic force are SW, the vibration itself is always quasi-sinusoidal, which results in complex averaging of the reaction and friction forces. Third, the orientation of the robot and the movement direction noticeably fluctuate, reducing the average force, and the acceleration in the chosen direction (normal to the grooves). Of course, the acceleration stage cannot last forever, for larger substrates, the velocity should stabilize at values not exceeding the estimation (49) above. For smaller currents (and/or frequencies) the robot spends more time on the ground, and the estimation $(27)_1$ and the physical picture behind it are more relevant.

1.13. Vertical acceleration and the start of the movement

With the sinusoidal driving force, the robot usually needs an initial "kick" to start the movement. This difference between the harmonic and SW excitations is crucial at low frequencies, and decreases near the resonance. It is related to the finite depth of the groove, out of which the robot feet should be pulled out, as discussed below.

Let us estimate the amplitudes of vertical displacement $Y(\omega)$ and acceleration a_y. For $\psi = \pi/2$ the vertical amplitude factor in Supplementary Table 7 is $C_y \approx 1$. Using the forces for small current of $I = 0.2\,\text{A}$ used in the theoretical Supplementary Figs. 41-44, one obtains near the resonance $Y(\omega_0) \approx \dfrac{F}{m_y m \omega_0 \gamma} = 0.64\,\text{mm}$ $a_y \approx \dfrac{F\omega_0}{m_y m \gamma} = 53\,\text{m}^2/\text{s}$. Such an *acceleration* $a_y > g$ is high enough to detach the robot from the ground. However, it may not suffice to pull the feet out of the grooves. Indeed, the estimated amplitude $Y(\omega_0)$ is comparable with the depth of the groove $b_{\text{gr}} = 0.47\,\text{mm}$, obtained for the best case with $a = 0.8$ (see Supplementary Fig. 18). The gravity lowers the robot COM over the half-period $T/2 = \pi/\omega$ when the robot feet ascend from the relative position $-Y(\omega)$ to $Y(\omega)$. The liftoff condition $a_y > g$ is usually fulfilled for most of the half-period. Feet elevation above the ground y_{elev} can then be estimated as follows.

$$y_{\text{elev}} \overset{1}{\approx} \int_{a_y > g} (v_y - gt)\,dt \overset{2}{\approx} 2Y(\omega) - g\frac{(T/2)^2}{2} \tag{50}$$

Even near the resonance, the gravitational term in Eq. $(50)_2$, $gT_0^2/8 \approx 0.59\,\text{mm}$ is comparable with both the resonant amplitude $Y(\omega_0)$ estimated above, and the groove depth b_{gr}.

Moreover, the estimation (50) yields significantly different thresholds for sinusoidal and SW excitations at low frequencies. In harmonic case the amplitude there is $2Y_{\sin}(\omega \ll \omega_0) \sim \dfrac{2F}{m_y m \omega_0^2}$, while for the SW case it is trice larger, $2Y_{\text{sw}}(\omega \ll \omega_0) \sim \dfrac{6F}{m_y m \omega_0^2}$, and even higher near the secondary resonances, see Supplementary Fig. 43a. Furthermore, for the sinusoidal excitation, the frequency of oscillations roughly coincides with ω, and $T_{\sin} \approx 2\pi/\omega$. For the SW, on the other hand, the oscillations *always* have strong eigen-frequency component ω_0, resulting in $T_{\text{sw}} \approx 2\pi/\omega_0 \ll T_{\sin}$ (see Supplementary Fig. 44). As a result, for $\omega \ll \omega_0$, the condition (50) to get out of the groove and start the movement becomes in these two cases:

$$\begin{aligned} &\text{sin}: \quad \frac{2F}{m_y m \omega_0^2} - \frac{\pi^2 g}{2\omega^2} > b_{\text{gr}} \quad \Rightarrow \quad F_{\sin} > \frac{m_y m}{2}\left(b_{\text{gr}}\omega_0^2 + \frac{\pi^2\omega_0^2}{2\omega^2} g \right) \\ &\text{SW}: \quad \frac{6F}{m_y m \omega_0^2} - \frac{\pi^2 g}{2\omega_0^2} > b_{\text{gr}} \quad \Rightarrow \quad F_{\text{sw}} > \frac{m_y m}{6}\left(b_{\text{gr}}\omega_0^2 + \frac{\pi^2}{2} g \right) \end{aligned} \tag{51}$$

For our parameters, at the realistic frequency of $f = 20\,\text{Hz}$, this results in one-leg "thresholds" $F_{\text{sw}} \geq 0.98\,\text{mN}$ and $F_{\sin} \geq 9.87\,\text{mN}$. The threshold $F > F_{\text{sw}}$ is reached for the currents around $I \approx 0.225\,\text{A}$, while the harmonic threshold $F_{\sin}$ exceeds the Lorentz forces even for the unrealistically high current of $I \approx 2.25\,\text{A}$, which is unusable for reasons of heating and stability.

This difference between the SW and sinusoidal excitation may decrease for much shallower grooves, or with the microstructured feet, which can provide directionally asymmetric friction even on planar surfaces.

1.14. Estimations of the damping coefficient

The main novelty in our governing equations (11) is the addition of the viscous terms $\eta_w w_t$, $\eta_u u_t$ with the damping coefficient γ, defined in Eq. (12) .These terms accompany the inertial "acceleration" terms; as a result, damping effects can be incorporated into a single parameter Z in Eq. $(14)_2$. The phenomenological coefficient γ should be inferred from the experimental data. This can be done in several ways, best understood from the (quite accurate) Lorentzian oscillator analogy (23).

As explained there, γ is full width at half-maximum of the harmonic frequency response curve for the energy, which corresponds to the $1/\sqrt{2} \approx 0.7$ level for the amplitude. Let us consider the experimental response curves in Supplementary Figs. 17a, b, for sufficiently small current $I = 0.2\,\text{A}$, which belongs to the linear regime. Their maxima are around $f = 37\,\text{Hz}$, and $1/\sqrt{2}$ level spans $34 - 40\,\text{Hz}$. This yields an estimate $\gamma \approx 2\pi \times 6 \approx 38\,\text{s}^{-1}$.

At the same time, γ defines the amplitude at resonance and the ratio of resonant and static amplitude responses according to $(24)_4$. Using the same $I = 0.2\,\text{A}$ curves in Supplementary Figs. 17a, b, we estimate the one-leg resonant displacement as $X(\omega_0) \approx 1.5\,\text{mm}$. The *low-frequency* data in these measurements are not very accurate (due to video frame sampling and exposure used). For *static* deflection, it is better to use the data from Supplementary Fig. 15d (and b), which predict one-leg deflection $X(0) \sim 0.42\,\text{mm}$ for $I = 0.2\,\text{A}$. Combining these values, we estimate $\gamma \approx \omega_0 X(0)/X(\omega_0) \approx 2\pi \times 37(0.36/1.5) \approx 56\text{s}^{-1}$.

Finally, in free vibrations, the amplitude decreases as $e^{-\gamma t/2}$ (and the energy as $e^{-\gamma t}$), as can be seen in Supplementary Fig. 40a. To visualize and analyze the amplitude decay, the $\log|x(t)|$ is shown in Supplementary Fig. 40b for several decay runs. The envelope of such a logarithm is a straight line; its slope gives the decay constant: $\log(x_0 e^{-\gamma(t-t_0)/2}) = \text{const} - \gamma t/2$. Such a linear fit is shown in Supplementary Fig. 40b for $\gamma \approx 50\,\text{s}^{-1}$, which approximately matches the decay of all 4 curves. Temporal variations in the decay rate in Supplementary Fig. 40 are due to beats from the slight 3D effects, present in real vibrations, which can be seen in the corresponding videos (see Supplementary Movie 3).

All these are estimates from below, for the following reasons. For larger amplitudes, the curves in Supplementary Fig. 16 are broader and the (effective) damping is higher. These data are for the clamped robot, while for the free-free *moving* robot additional damping mechanisms exist, which may be comparable in value. Because for the robot speed the resonant amplitude is of primary importance, we relied mainly on the resonant to static amplitude ratio estimation, using the value of $\gamma \approx 70\,\text{s}^{-1}$ in calculations, but all estimations produce values in a similar range. Importantly, the width of the resonant curves for the amplitude of clamped vibrations in Supplementary Fig. 17 and speed curves in Supplementary Fig. 20 are comparable (having in mind its functional shape (49)), which justifies our argumentation.

Several energy loss mechanisms exist in the system: internal elastomer damping, both viscous and "viscoelastic"; constant (Coulomb) friction between the robot feet and the substrate; induced (adjoint) mass

of the surrounding air, emission of sound by the vibrating robot; work spent on the center of mass displacement (both vertical and horizontal), which partially dissipates via the reaction forces of the substrate. All this may produce functionally different damping behavior – from the constant (Coulomb) friction to quadratic drag, or viscoelastic effects, resulting in delayed response and creep. However, within the practical operating parameter range (frequency, current, etc.), all these mechanisms can be approximated using a convenient linear damping framework.

For reference purposes, let us provide guideline estimations of the damping due to emission of sound. Radiated sound power is often characterized by the dimensionless acoustic radiation efficiency σ, such that $P_{\text{sound}} = \sigma Sc\rho_{\text{air}} < v^2 >$. Here S is the surface area, c is the speed of sound, and $< v^2 >$ is the surface-averaged velocity (amplitude, or RMS, different definitions exist), with clear physical interpretation. For the structure sizes comparable with the sound wavelength $\sim c/f$, the value $\sigma \sim 1$, but for smaller structures typically $\sigma \ll 1$ (which is the case for our system).

Because the total energy stored in both legs of the robot is of the order of $E \approx m < v^2 >$, the energy decays according to:

$$\overset{\bullet}{E} \overset{1}{=} m < \overset{\bullet}{v^2} > \overset{2}{=} -\sigma Sc\rho_{\text{air}} < v^2 > \qquad \Rightarrow \qquad \gamma_{\text{sound}} \overset{3}{\approx} \frac{\sigma Sc\rho_{\text{air}}}{m} \underset{\sigma=1}{\overset{4}{\approx}} 46\,\text{s}^{-1} \qquad (52)$$

Here, γ_{sound} is the rate of energy decay due to sound emission, which coincides with the definition of γ in our notations. Equation (52) produces an exponential decay of the velocity and amplitude, because the sound is a "small amplitude, linear" effect, it does not include a (sometimes stronger) quadratic inertial drag. The numerical value (52)4 is estimated for $\sigma = 1$, which shows, that though in some situations sound emission can contribute to damping appreciably, but in our case with $\sigma << 1$ the sound emission is of minor influence.

1.15. Parameter used in analytic calculations

Parameters used in theoretical calculations presented in Supplementary Figs. 40-43 are (one leg half-robot mass and length):, $m = 89.85\,\text{mg}$, $L = 7.3\,\text{mm}$, $b = 9\,\text{mm}$, $b_I = 6\,\text{mm}$ (average length of three wire currents), $h = 0.92\,\text{mm}$, $\mu = 80.0\,\text{kPa}$ (about 1.2 times larger, than the listed Ecoflex value $\mu = 66.5\,\text{kPa}$, see Supplementary Fig. 39 and the related discussion), $B = 0.3\,\text{T}$, $I = 0.2\,\text{A}$, $F = 3Bb_I I(1 - \frac{1.4}{L[\text{mm}]}) = 0.873\,\text{mN}$ (force calculated for three wires and diminished by the edge-distance factor, which provides about the same torque), $\gamma = 70\,\text{s}^{-1}$. For these parameters, the predicted free-free frequencies are $f_0 = 45.7\,\text{Hz}$, $f_{\max} = 45.0\,\text{Hz}$, and for the clamped-free regime $f_0 = 37.3\,\text{Hz}$, $f_{\max} = 36.3\,\text{Hz}$.

In Supplementary Fig. 44 the frequency ω is normalized as ω / ω_0, $\gamma = 0.2\omega_0$, dimensionless time is $\omega_0 t$, the frequency for the time plots is chosen as $\omega = \omega_1 / 5$, where one of the low-frequency resonances lies. Note, that ω_1 is a bit smaller than ω_0, as follows from the definition $\omega_1 = \sqrt{\omega_0^2 - \gamma^2/4} \overset{\gamma=0.2\omega_0}{=} \sqrt{0.99}\omega_0$. Deflection is in dimensionless units, i.e., the ordinate shows dimensionless ratio $\frac{x}{F / m\omega_0^2}$, and the dimensionless velocity is $\frac{v}{F / m\omega_0}$.

1.16. Takeaways from the theoretical analysis

The developed theoretical framework provides a clear physical picture of the process. Its key feature is the resonant nature of the movement, characterized by the resonant frequency and damping. The detailed expressions for the resonant frequency in terms of material and geometrical parameters are provided, which paves the way for improvements and optimization. A single semi-empirical damping coefficient is inferred from the experiment, while physical damping mechanisms and the framework for more detailed modeling are discussed. The implications of square-wave excitation are elucidated, explaining complex low-frequency response, and SW importance for the onset of movement on grooved substrates with asymmetric friction.

1.17. Locomotion principle of the SEMR with the L-shaped feet

For the unidirectional movement, certain asymmetry is required. For the SEMRs with the sawtooth-shaped feet, it is provided by the directionally unequal friction between the feet and the custom-printed asymmetric substrate, which restricts the applicability of such a system. To overcome these limitations, we developed a design, where the asymmetry is provided solely by the geometry of the robot itself. In short, the feet are designed such that the expansion and contraction of the robot body create unequal frictions between the feet, resulting in the forward motion. Such SEMRs with asymmetric L-shaped feet can walk and run on planar unstructured substrates made of various materials with different textures.

The principle of operation is shown in the Supplementary Fig. 23. Panel Supplementary Fig. 23a shows the free body diagram of a robot. Its general characteristics and force designations are similar to the diagram presented in Supplementary Fig. 19, with several key modifications: The substrate (gray line) is planar and unstructured. The feet are shown by the black polylines with three segments, the upper segments are used to glue the feet to the robot body. The central segments are relatively long (typically in the range from 0.3 to 0.6 of the robot body radius R), and are attached in the essentially asymmetric way: The rear foot is affixed to the inside of the curved robot body, while the front foot is attached on the outside; the resulting asymmetry defines the forward direction of the robot movement. The last segment points towards the ground and provides well-localized "pivotal" contact. The feet's weight is not negligible in relation to the robot body so that the center of mass (COM) is shifted slightly to the right from its "symmetric" position in all figures.

The principal walking/running sequence for the SEMR with the asymmetric L-shaped feet is illustrated in the Supplementary Fig. 23b. Here, the feet are shown schematically, by the straight segments from the edges of the body to the lowest feet points, and the overall COM is shown by the circles with the same color coding as the corresponding robot body.

Let us start with the expansion stage shown in the left panel. At the beginning of the expansion, the robot is in the contracted state and both feet touch the ground (solid magenta curve). The robot expands under the action of Lorentz forces F_{2L} and F_{1R} with the directions shown in the Supplementary Fig. 23a, (some phase delays between the vibration and the forces may exist for resonant oscillations, but this is immaterial for the argumentation). The Lorentz forces F_{2L} and F_{1R}, as well as their torques with respect to the COM (almost) balance each other.

The shape of the feet is such that the contracted magenta robot is tilted counterclockwise. If the robot were to expand without the gravity force G and substrate reaction, its COM would remain at the same position, and the body would have the same angle with respect to it, as defined by the conservation of angular momentum (calculated with respect to COM). Such a hypothetical state is shown by the dashed cyan curve; the cyan COM coincides with its initial (magenta) position. However, because the robot feet cannot immerse into the substrate, the rear foot is pressed towards the ground, while the front one is lifted into the air. As a result, the normal reaction force for the rear foot becomes $F_{s2} \approx G$, and the associated friction force $F_{f2} \approx fG$ (see panel Supplementary Fig. 23a, here $0.1 < f < 0.5$ is the coefficient of dry friction). Both values become much larger than for the front foot, where $F_{s1} \approx 0, F_{f1} \approx 0$. As a result of this induced frictional asymmetry, the front foot slides, and the robot is propelled forward (to the right). The rear foot contact with the ground with strong friction $F_{f2} \approx fG$ serves as a pivot point. Simultaneously, the gravity torque, which is of the order of RG, brings the front foot to the ground within some typical gravitational time t_G. All other torques are much less relevant, as the Lorentz torques roughly cancel each other, the rear foot reaction and friction are applied at the pivot point, and for the front foot $F_{s1} \approx 0, F_{f1} \approx 0$. The robot position at the end of the expansion actuation phase is shown by the deep blue solid curve.

The gravitational time t_G should roughly match the half-period of the elastic oscillations. If t_G is too short, the front foot is brought to the ground too early, and both feet remain on the ground for the most of the expansion-contraction stages, diminishing the effect of geometrical asymmetry in the friction. If this time is too long, the contraction starts with the front foot still in the air, and the robot becomes airborne, resulting in a "jumping", or even a "flying" regime. While the robot velocity is higher in such cases, the movement is much less controllable. All this imposes additional requirements onto the robot design parameters, such as

feet length and height. Due to the complexity of the processes and the multitude of the factors involved, these parameters were optimized experimentally, resulting in the feet geometry described in Supplementary Fig. 24.

For the contraction stage (shown in the right panel of Supplementary Fig. 23b), the sequence is similar, but the roles of the rear and front feet are interchanged. The contracting motion starts from the fully stretched magenta state, which corresponds to the final deep blue state in the left panel. The geometry of the feet is such that the robot is tilted clockwise. The hypothetical contracted position without substrate and gravity has the same COM and orientation and is shown by the dashed cyan curve. Here, the front foot is pressed into the ground, and its outermost part serves as a pivot point. The normal reaction and friction forces there are $F_{s1} \approx G, F_{f1} \approx fG$, which is much larger than for the rear foot, where $F_{s2} \approx 0, F_{f2} \approx 0$. The rear foot is again lifted above the surface, although this effect is less pronounced than in the expansion stage. The dominant gravity torque about the frontal pivot point is again of the order of $\sim RG$ and rotates the robot counterclockwise bringing the rear foot down, resulting in the final contracted state with both feet on the ground (deep blue solid curve). This final state corresponds to the initial magenta state in the left panel, translated rightwards by the sum of COM displacements, indicated in both panels by an arrow.

Thus, over one cycle, the robot advances the front foot forward during the expansion, and pulls up the rear foot during contraction. The forward motion consists of the cycling alternation of such expansion-contraction phases. The associated periodic weight shifting and friction rebalancing from one leg to another resembles human walking and running cycles.

The suggested universal design allows ultrafast, controlled locomotion on various pristine unstructured substrates with dissimilar tribological properties – wood, sand and office paper, PDMS, metal, plastic and glass (see Fig. 3k in the main text). As the slip between the feet and the substrate is higher than for the structured surfaces with full mechanical interlock, the highest speed achieved for the L-shaped design is 35 BL/s, which is somewhat lower than for the sawtooth-shaped SEMR on 3D printed substrate.

1.18. Characterization of the magnetic field

We characterized the magnet with a Gauss meter (Gaussmeter HGM09s, Goudsmit Magnetic Systems). The magnetic flux densities of magnet 1 and magnet 2 are 290 mT and 462 mT, respectively, in the Z direction measured by the Gauss meter at a distance of 6 mm to the surface of the magnet. Multiple positions on top of the magnet are measured. The measured magnetic field strength is used to fit the remanent magnetization. We use the commercial finite-element package COMSOL to obtain the distribution of the magnetic field[4]. In the simulation, we assume that the magnet material is homogenous and axisymmetric. Combining experiment and simulation, we obtain the remanent magnetization of the magnet 1, 1.31 T. The compound magnet 2 is treated as a single magnet with an effective remanent magnetization of 1.2 T. In Supplementary Fig. 45, we plotted the magnetic field from simulations at the height of 1 mm and 5 mm over the surface of the magnet, where most of the robots operate.

1.19. Joule heating and further performance optimization

Let us estimate the effect of Joule heating on the robot. Heating increases the temperature of the robot (Supplementary Fig. 8), which negatively affects the robot performance in several ways. It decreases the robot efficiency, in extreme cases, it may modify, or even destroy its elastomeric body. Temperature also accelerates battery degradation, which shortens the maximum operation time.

Let us assume that the length of the coil segment on which the Lorentz force F acts is L, and the number of turns in the coil is n. The resistivity of the LM is ρ, the channels cross section is S, and the total resistance of the coil(s) is R. Let us write some obvious relations omitting dimensionless "coil-shape-related" constants.

$$U = IR, \qquad F = nLBI = \frac{nLBU}{R}, \quad P_{\text{heat}} = I^2 R = \frac{U^2}{R}, \qquad R = \frac{\rho n L}{S} \qquad (53)$$

The current I, voltage U and Joule power P_{heat} can be expressed in terms of the (constant) required force F and the most basic geometric parameters of the system n, L, S as follows.

$$I=\frac{F}{nLB},\qquad U=\frac{\rho F}{SB},\qquad P_{\text{heat}}=\frac{\rho F^2}{nLSB^2} \tag{54}$$

The last expression shows, that the Joule power can be decreased by increasing the magnetic field strength B, or the product nLS, which is the metal volume. Assuming fixed robot size, which defines L, this can be done either via increase in n, or via increase in S. An increase in n decreases the operational current, but does not affect the voltage, while increase in S decreases the voltage, but does not affect the current. For the tethered case the current I is usually constant, while for an untethered case, the voltage U, rather than current is an almost constant limiting factor. Thus, for the tethered robot one can use either n or S to reduce P_{heat}, but for the untethered case, if larger forces are required from the same battery, an increase in S is more beneficial.

In the present design, the connecting rigid electrodes are inserted into the LM channels near the start and end of the metal coils. For robust and reliable contact such an insertion requires an appreciable finite portion of the channel (in the mm range). The robot body is thin and curved, and its bending properties should not be hindered by the contacts and the inserted solid wires. This necessitates the electrode insertion in the "lateral" direction, perpendicular to the bending of the robot. This is realized with the two-square-coils profile of the channels occupied by the LM (Fig. 3a). In the future, the SEMR can be simplified to have only one rectangular coil with a better connection between the power supply and the LM channels. This eliminates the central parts of the wires, which reduces the inner resistance and Joule power.

As an example, here we consider the case of SEMR TST. The resistance of the robot is about R=1.1 Ω, resulting in a power draw $P_{\text{heat}}=I^2R=0.1\ W$ at a current of 0.3 A. This power consumption can be decreased by increasing the magnitude of the magnetic field. A tenfold increase in the magnetic field (3 T, which is typical for an MRI machine) reduces the power required to produce the same Lorentz force to 0.001 W (1% of the current power).

1.20. Appendix: auxiliary theoretical expressions

The roots of the bicubic equation (14) can be explicitly written as follows (in Mathematica complex branches convention):

$$k_j^2=\frac{1}{3}\left(-2+\zeta+\frac{1+3Z^2}{\zeta}\right),\qquad \zeta=e^{i\frac{2\pi}{3}j}\left(1-\frac{45}{2}Z^2+3\sqrt{3}\sqrt{-Z^2(2-\frac{71}{4}Z^2+Z^4)}\right)^{1/3} \tag{55}$$

Here $j=1,2,3$, or $j=0,1,2$, or $j=0,\pm1$. We also list the (cubic) Vieta equalities for the roots:

$$\begin{aligned}
&\sum_{j=1}^{3}k_j^2\overset{1}{=}-2, && \sum_{1\to2\to3}^{3}k_1^2k_2^2\overset{2}{=}1-Z^2, && \prod_{j=1}^{3}k_j^2\overset{3}{=}-Z^2\\
&\prod_{j=1}^{3}(k_j^2+1)\overset{4}{=}-2Z^2, && \sum_{1\to2\to3}^{3}(k_1^2+1)(k_2^2+1)\overset{6}{=}-Z^2, && \prod_{j=1}^{3}(k_j^2-1)\overset{5}{=}-4
\end{aligned} \tag{56}$$

The last three expressions follow from the first three, or the Eq. (14), rewritten for the variables $k^2\pm1$.

The expressions for the displacement derivatives are:

$$\begin{aligned}
&u\overset{1}{=}\sum_{j=1}^{3}(S_j\sinh k_j\varphi+C_j\cosh k_j\varphi), && w\overset{2}{=}\sum_{j=1}^{3}k_j(S_j\cosh k_j\varphi+C_j\sinh k_j\varphi)\\
&w_\varphi\overset{3}{=}\sum_{j=1}^{3}k_j^2(S_j\sinh k_j\varphi+C_j\cosh k_j\varphi), && w_{\varphi\varphi}\overset{4}{=}\sum_{j=1}^{3}k_j^3(S_j\cosh k_j\varphi+C_j\sinh k_j\varphi)\\
&w_{\varphi\varphi\varphi}\overset{5}{=}\sum_{j=1}^{3}k_j^4(S_j\sinh k_j\varphi+C_j\cosh k_j\varphi), && w_{\varphi\varphi\varphi\varphi}\overset{6}{=}\sum_{j=1}^{3}k_j^5(S_j\cosh k_j\varphi+C_j\sinh k_j\varphi)
\end{aligned} \tag{57}$$

Determinant for the free-free case: The expression in square brackets in Eq. $(17)_6$ can *always* be simplified using Eq. $(14)_1$: $k_j^2(k_j^2+1)-Z^2\mapsto-2Z^2/(k_j^2+1)$, but it does not noticeably help. For free-free case all $C_i=0$, and the system can be written in a simpler matrix form:

$$m_{ij}S_j = f\begin{pmatrix} 0 \\ \sin\psi \\ \cos\psi \end{pmatrix}, \qquad m_{ij} = \begin{pmatrix} k_j(k_j^2+1)\cosh k_j\psi \\ k_j^2(k_j^2+1)\sinh k_j\psi \\ k_j[k_j^2(k_j^2+1)-Z^2]\cosh k_j\psi \end{pmatrix} \tag{58}$$

Using determinant properties and relations (56), one can reduce the underlying determinant to:

$$\det m_{ij} = iZ^3\left(\sum_{1\to2\to3}^{3} k_1(k_1^2-1)(k_2^2-k_3^2)\tanh k_1\psi\right)\prod_{j=1}^{3}\cosh k_j\psi \tag{59}$$

The expression in brackets implies cyclic permutations. Some other equivalent forms can be constructed.

In a systematic analysis, one introduces radial and tangential responses to radial and tangential driving forces $f_{w,u}$. Due to the linearity of the system, in our geometry with the horizontal force, the Cartesian displacements are:

$$\begin{pmatrix} f_w \\ f_u \end{pmatrix} \overset{1}{=} f\begin{pmatrix} -\sin\psi \\ \cos\psi \end{pmatrix}, \quad \begin{pmatrix} w \\ u \end{pmatrix} \overset{2}{=} \begin{pmatrix} w_w & w_u \\ u_w & u_u \end{pmatrix}\begin{pmatrix} f_w \\ f_u \end{pmatrix}, \quad \begin{pmatrix} x \\ y \end{pmatrix} \overset{3}{=} \begin{pmatrix} -\sin\varphi & \cos\varphi \\ -\cos\varphi & -\sin\varphi \end{pmatrix}\begin{pmatrix} w \\ u \end{pmatrix} \tag{60}$$

For the end displacements at $\varphi = \psi$ this yields:

$$\begin{pmatrix} x \\ y \end{pmatrix} \overset{1}{=} \begin{pmatrix} -\sin\psi & \cos\psi \\ -\cos\psi & -\sin\psi \end{pmatrix}\begin{pmatrix} w_w & w_u \\ u_w & u_u \end{pmatrix}\begin{pmatrix} -\sin\psi \\ \cos\psi \end{pmatrix} f \overset{2}{=}$$
$$\begin{pmatrix} w_w\sin^2\psi - (w_u+u_w)\sin\psi\cos\psi + u_u\cos^2\psi \\ (w_w-u_u)\sin\psi\cos\psi - w_u\cos^2\psi + u_w\sin^2\psi \end{pmatrix} f \tag{61}$$

The effective mass factors $m_{x,y}$ for x, y-directions with respect to a horizontal driving force f (see Eqs. (18) -(19)) can be constructed from the similar effective mass factors: radial in response to a radial force m_{ww}, to a tangential force m_{wu}, and so on, as follows:

$$\begin{pmatrix} m_x^{-1} \\ m_x^{-1} \end{pmatrix} = \begin{pmatrix} -\sin\psi & \cos\psi \\ -\cos\psi & -\sin\psi \end{pmatrix}\begin{pmatrix} m_{ww}^{-1} & m_{wu}^{-1} \\ m_{uw}^{-1} & m_{uu}^{-1} \end{pmatrix}\begin{pmatrix} -\sin\psi \\ \cos\psi \end{pmatrix} \tag{62}$$

We do not do perform this decomposition, and report the dimensionless mass factors $m_{x,y}$ in Supplementary Table 7 directly.

2. Supplementary Figures

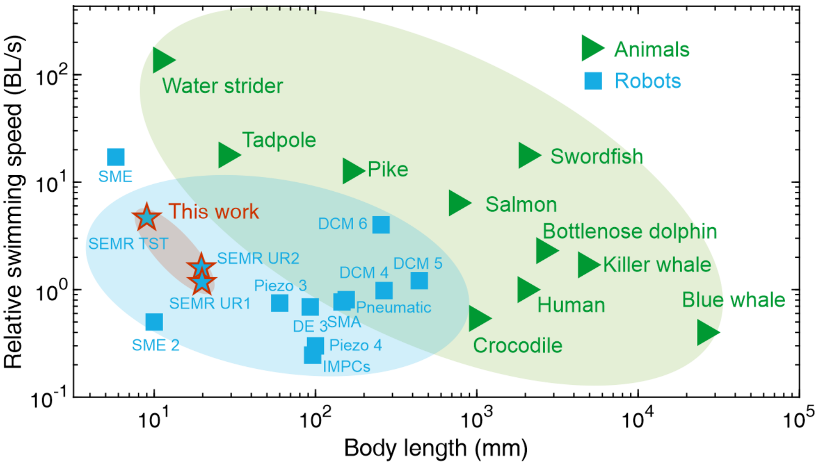

Supplementary Fig. 1. Comparison of the maximum relative swimming speeds of various animals and robots.

Related data is provided in Supplementary Table 2. Shaded areas encompass the ranges for different categories, as indicated by the symbols in the legend, as well as for our SEMRs ("This work"), which are labeled with the stars.

a

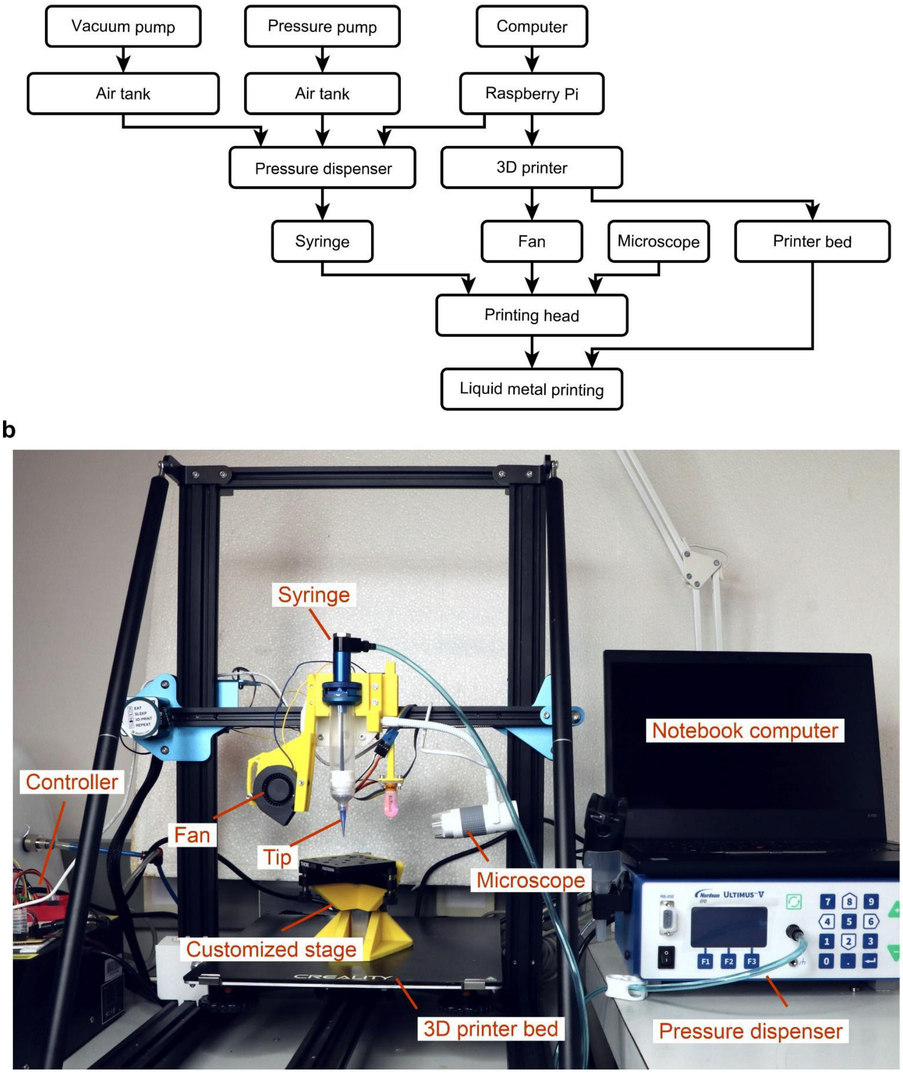

Supplementary Fig. 2. LM printing system.

a, Components. **b**, Experimental setup.

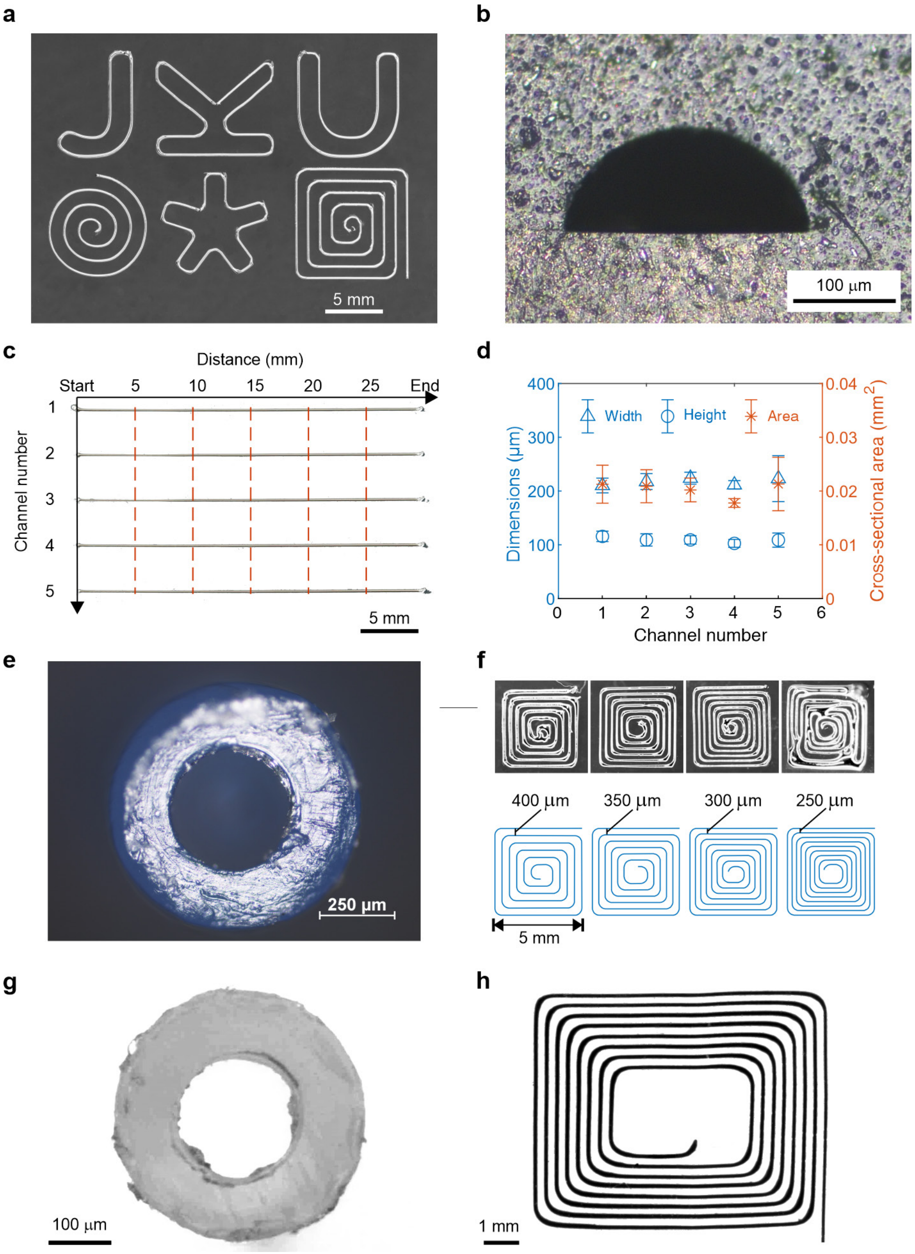

Supplementary Fig. 3. Characterization of the LM printing system.

a, Photo of the printed word 'JKU' and three patterns including Archimedean spiral, star, and 4-angle square spirangle. **b**, Cross-section of a printed LM channel within an elastomer substrate in a microscopic view. **c**, Printed straight LM channels. A cut for measurement is made every 5 mm (dotted lines). Error bars represent the standard deviation of five measurements. **d**, Dimensions (width and height) of the printed LM channels at every cutting point in panel c. **e**, Microscopic picture of the large printhead tip. **f**, Test of the minimum permissible distance between the adjacent printed LM channels. The top row shows the real printed patterns and the bottom row the designed ones. **g**, Microscopic picture of the small printhead tip with an inner diameter of 200 µm. **h**, A LM traces printed by the 3D LM printer with a small printhead. The distance between two traces is 300 µm.

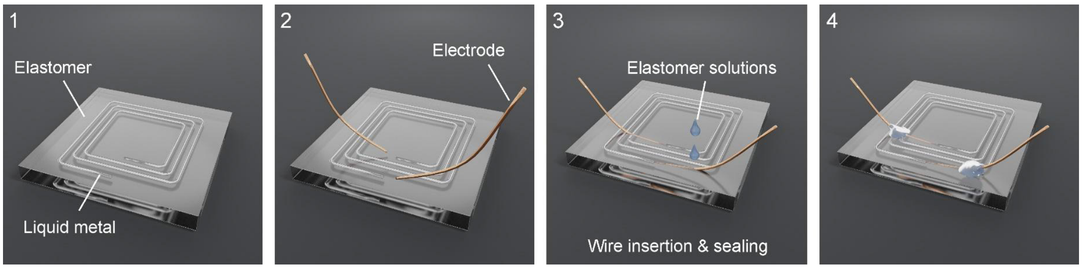

Supplementary Fig. 4. Connection of electrodes and LM channels.

Here, we use a flat SEMA as an example to illustrate how to connect the LM channel to the electrodes. First, two electrodes are inserted into the SEMA and then a few drops of elastomer solutions are put around the roots of the electrodes. At last, the solution is cured at room temperature, which enables the good sealing of LM. In this revision, the body of SEMR was compressed by a 2000 N force without leakage of liquid metal, which demonstrates the robustness of the fabrication.

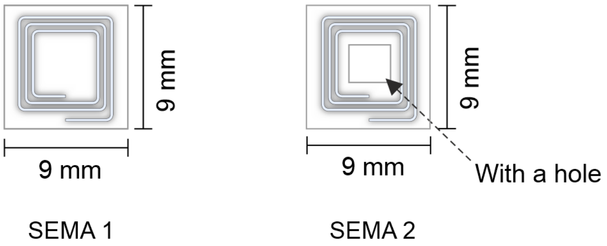

Supplementary Fig. 5. Geometries of SEMA 1 and SEMA 2.

The left and right schematic diagrams show the geometries of SEMA 1 and SEMA 2, respectively. The dimensions of the two SEMAs are the same, 9 mm × 9 mm × 0.8 mm. There is a hole in the center of SEMA 2 with a size of 1.4 mm × 1.4 mm × 0.8 mm.

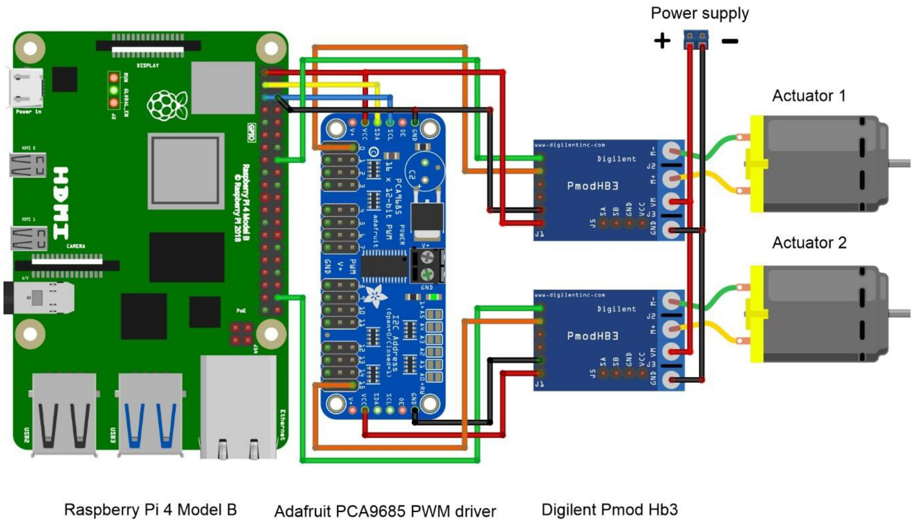

Supplementary Fig. 6. PWM control system for the robots.

Details about this setup are in the section: PWM control system for actuation in Materials and Methods. The individual components depicted in the image are distributed as part of the Fritzing parts library (https://github.com/fritzing/fritzing-parts), which is licensed under the "Creative Commons Attribution-ShareAlike 3.0 Unported (CC BY-SA 3.0)" license.

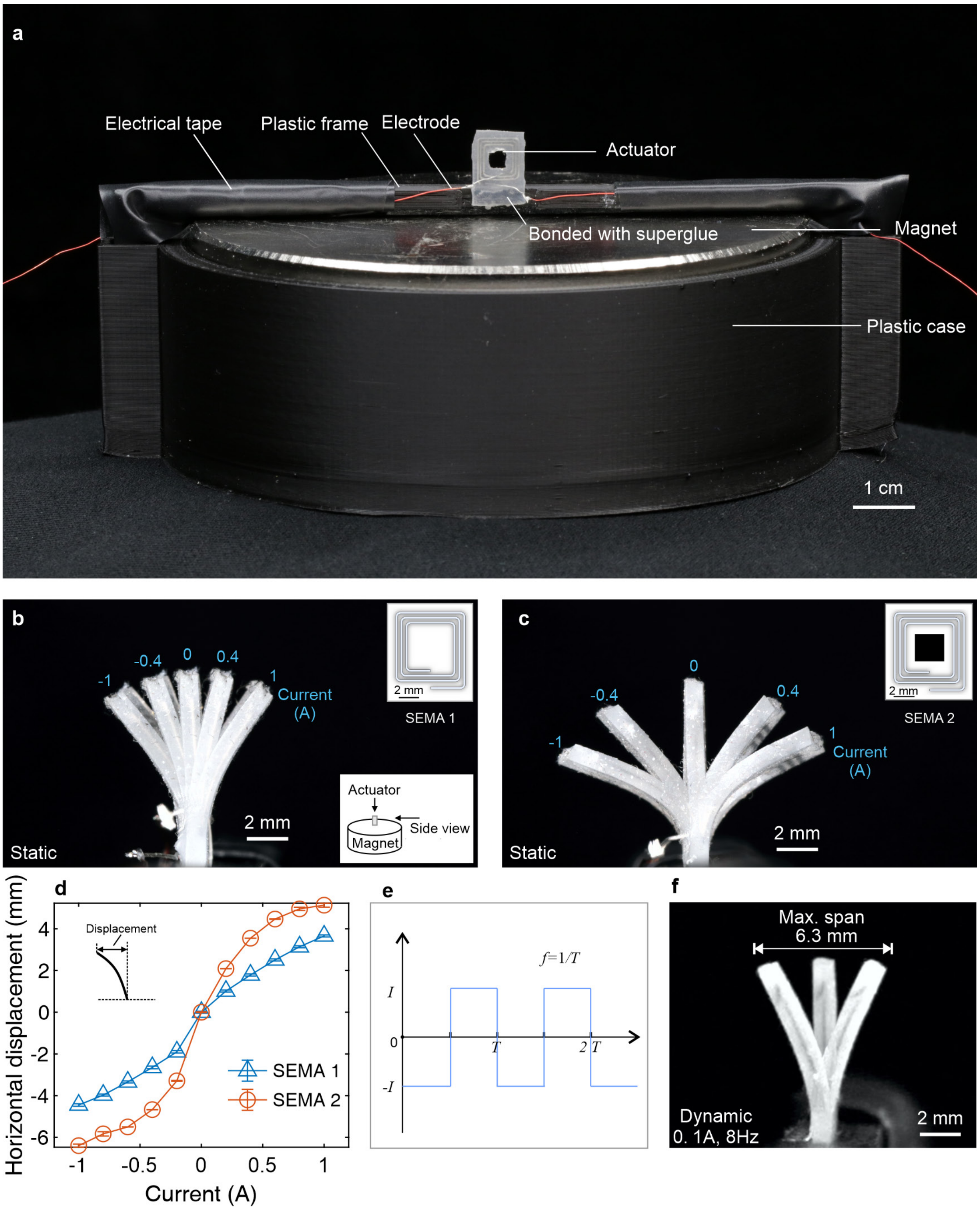

Supplementary Fig. 7. Bending test for millimeter-scale SEMAs.

a, Experimental setup of the bending test. The actuator is bonded to the plastic frame with super glue. **b**, Bending of the SEMA 1 for the indicated applied static currents. **c**, Bending of the SEMA 2 for the indicated applied static currents. **d**, The curves of horizontal displacement vs. the current of two SEMAs. Error bars represent the standard deviation of four measurements. **e**, Schematic diagram of the driving square-wave current in the bending test (used for most of the experiments in this work). **f**, Maximum span of SEMA 2 responding to a square-wave current with low amplitude (0.1 A, 8 Hz).

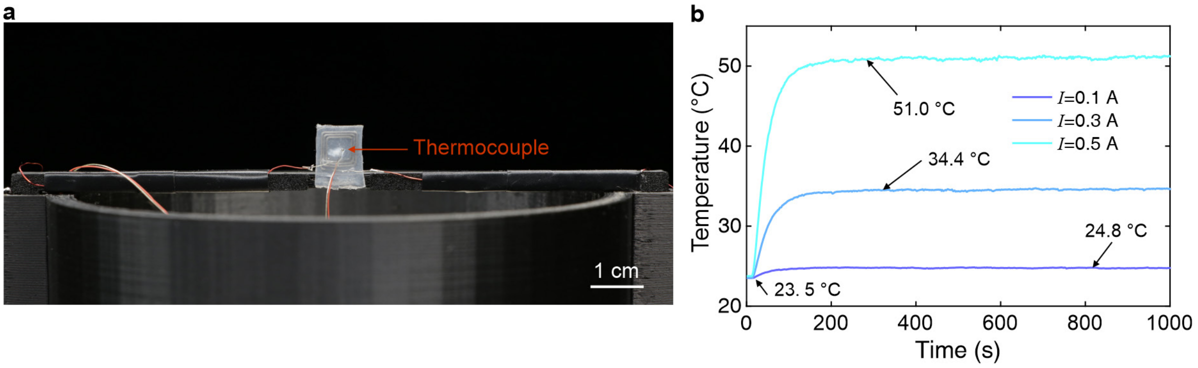

Supplementary Fig. 8. Temperature measurement of SEMA 1.

a, The setup of the temperature measurement where the actuator is fixed on a frame in the air. A thermocouple is affixed to the center of the SEMA 1. **b**, The temperature vs. time curve for the SEMA 1 subjected to different static currents, $I = 0.1, 0.3, 0.5$ A . The final equilibrium temperatures are indicated individually for different currents. The initial (room) temperature 23.5 °C is the same for all cases.

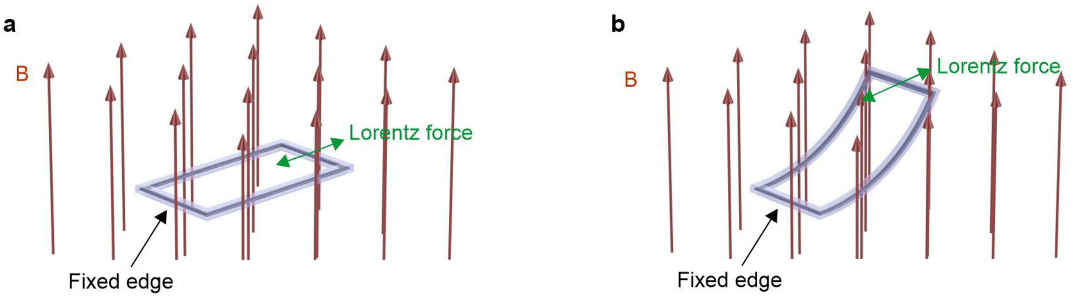

Supplementary Fig. 9. Free body diagram of a coil carrying a current in a homogenous magnetic field. **a**, A flat coil is perpendicular to the direction of the magnetic field. The direction of the Lorentz force lies in the coil plane and leads to an in-plane deformation only. **b**, A curved coil is subjected to a similar Lorentz force, which induces a bending deformation.

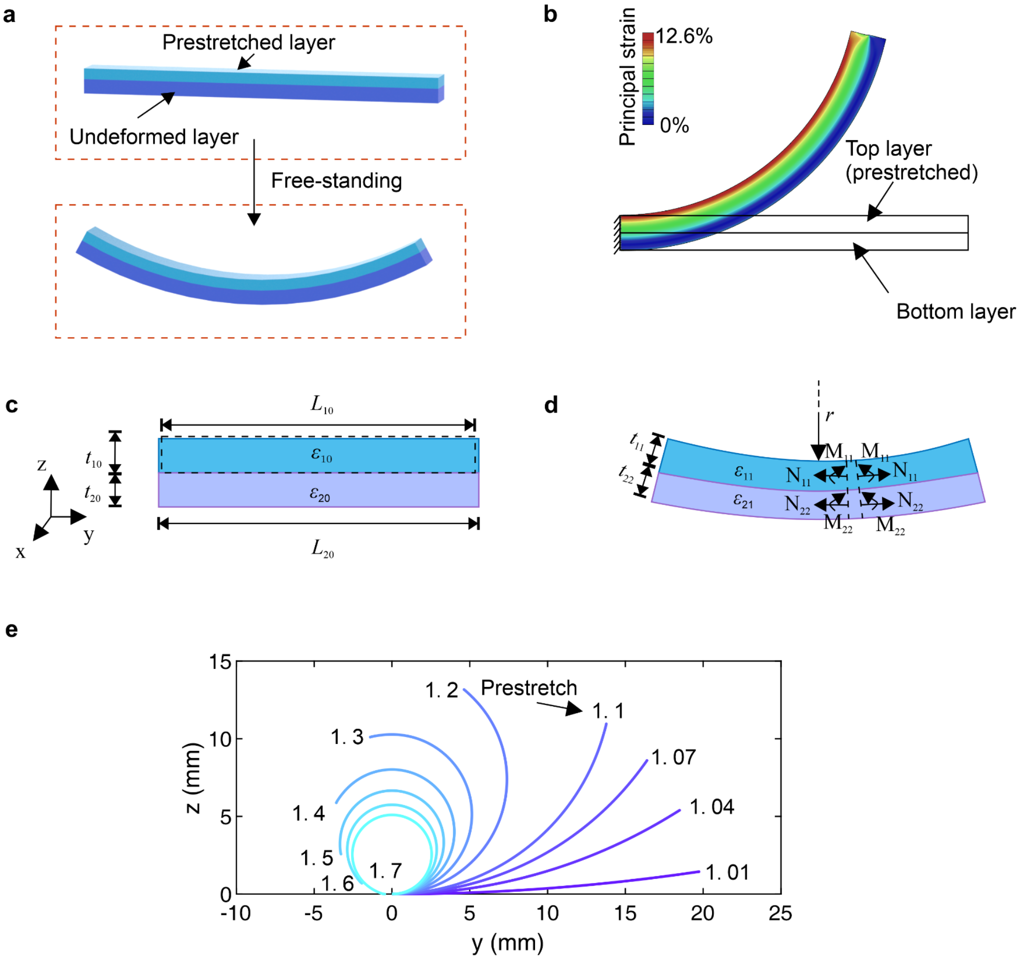

Supplementary Fig. 10. Deformation of a prestretched bilayer beam.

a, Schematic diagram of the fabrication of a curved bilayer beam. The top picture shows the bonding of the prestretched beam and an undeformed one. The bottom picture shows the free-standing state of the curved bilayer beam. **b**, Simulated bilayer beam bending as the prestretch increases. Schematic diagram of the bilayer beam in stretched state **c**, and free-standing state **d**, with the annotations used in the theoretical analysis. **e**, Profiles of the curved bilayer beam with various prestretches from 1.01 to 1.7. The curves are drawn along the interface between the two layers.

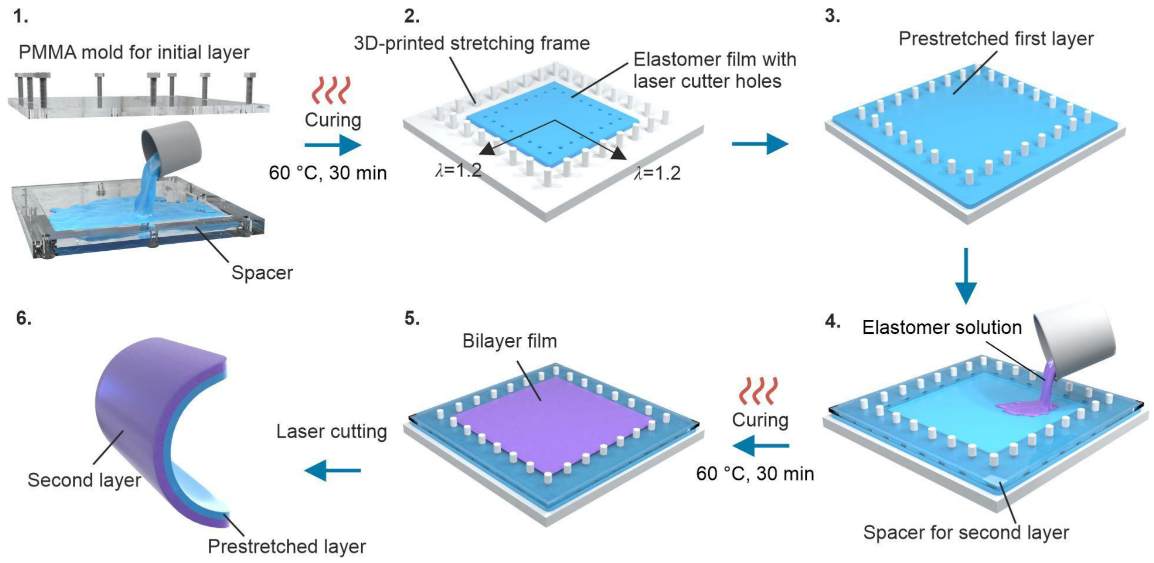

Supplementary Fig. 11. Fabrication of a curved bilayer film.

Step 1**,** The initial layer is first cured in a PMMA mold. Steps 2-3, the cured film is stretched and affixed to a different frame. Steps 4-5, The elastomer solution is poured on top of the prestretched layer and cured. Step 6, the desired curved bilayer film is obtained by laser cutting.

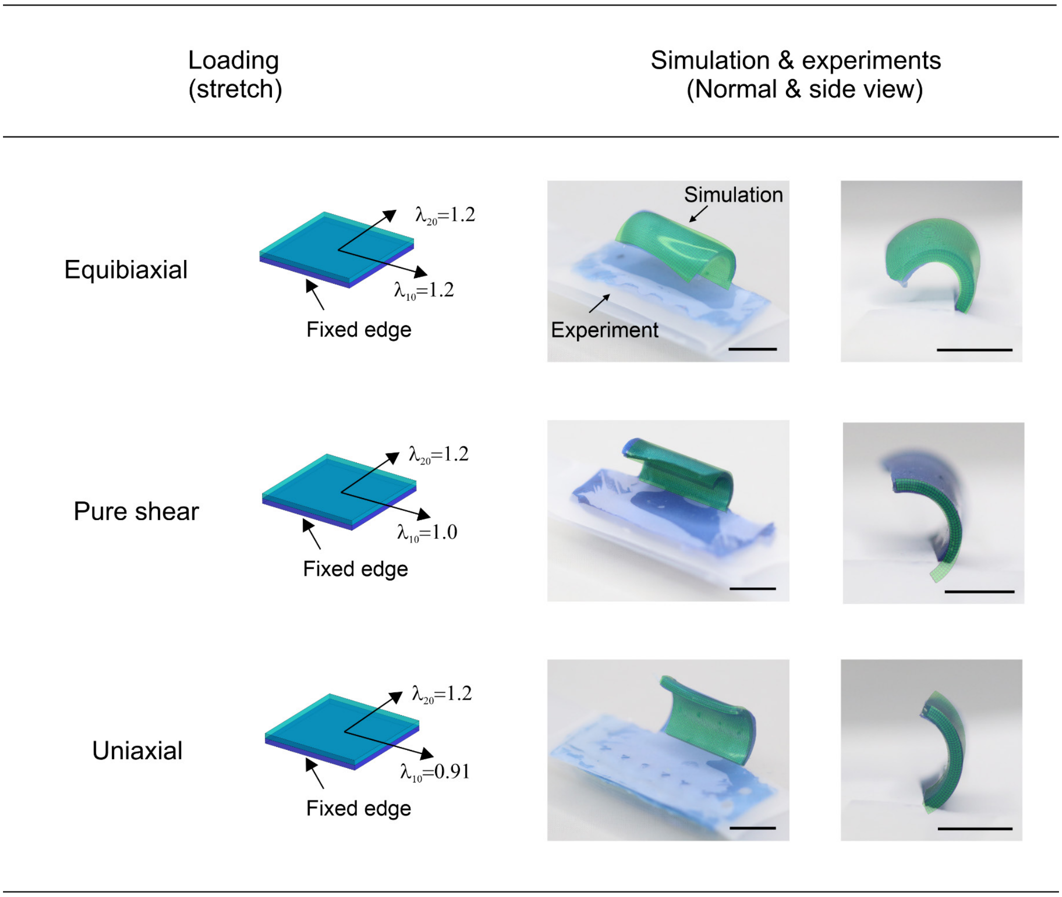

Supplementary Fig. 12. Shapes of bilayer films with different prestretches.

The top layer of the bilayer film is subjected to different prestretches including equibiaxial, pure shear, and uniaxial, all with the 1.2 value. The simulated results (green color) are overlaid on top of the experimental photographs. The scale bar is 10 mm.

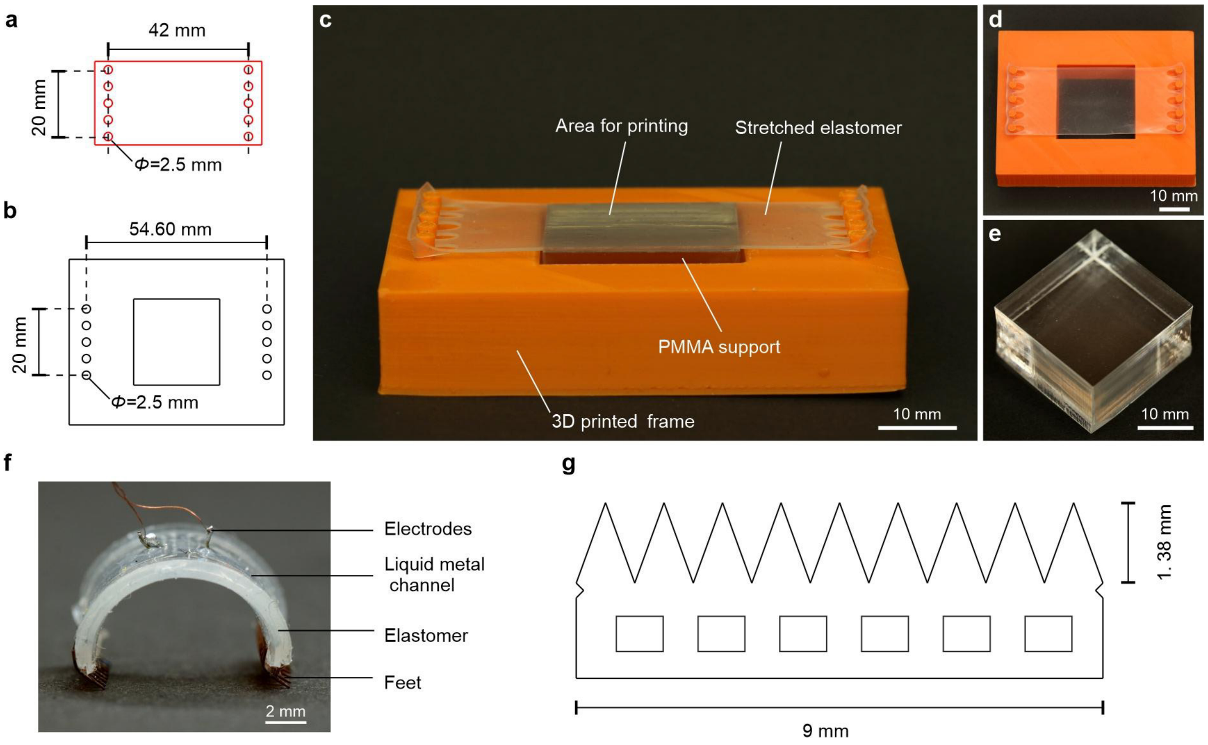

Supplementary Fig. 13. Fabrication of the curved SEMR TST.

a, Geometry of the unstretched elastomer film. **b**, Geometry of the 3D printed frame used to stretch the film. **c**, Setup of the printing stage with a stretched elastomer film and PMMA support. **d**, Top view of the film stretched and attached to the frame. **e**, PMMA support. The scale bar is 10 mm. **f**, Side view of the robot. **g,** Geometry of the sawtooth-shaped feet of the robot. The front foot and rear foot of the robot are identical.

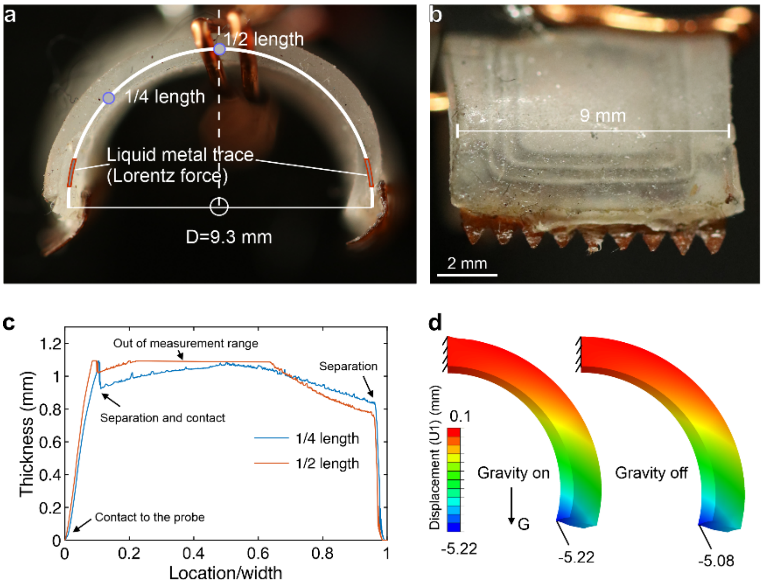

Supplementary Fig. 14. Geometry of the SEMR TST.

a, Side view of the SEMR. The positions of the horizontal LM channels located inside are indicated. Also, the positions at 1/4 and 1/2 body length of the SEMR are indicated. **b**, Front view of the SEMR. **c,** The measured thickness profile of the SEMR at the positions of 1/4 and 1/2 body length. **d**, Unloaded SEMR, simulated with and without gravity. The gravity direction is indicated by the arrow marked 'G', and the horizontal displacement is referred to the flat film before applying the prestretch (Supplementary Fig. 10b).

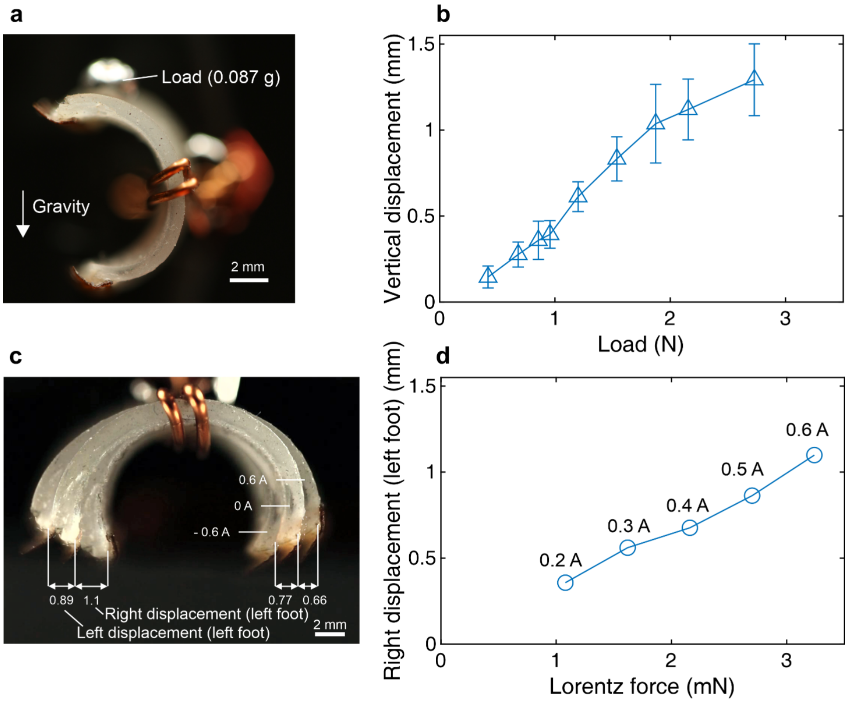

Supplementary Fig. 15. Static loading test of the SEMR TST.

a, Static load using metal beads of different mass. The beads are placed upright on the LM channels. **b**, Vertical displacement at the location of the bead versus the load (weight force). Error bars represent the standard deviation of three measurements. **c**, Deformed SEMR with respect to the undeformed one driven by different amplitudes of Lorentz forces corresponding to the driving currents -0.6 A and 0.6 A. **d**, Displacement of the left foot to the right versus the Lorentz force. The driving currents, corresponding to the Lorentz force, are indicated above the data points.

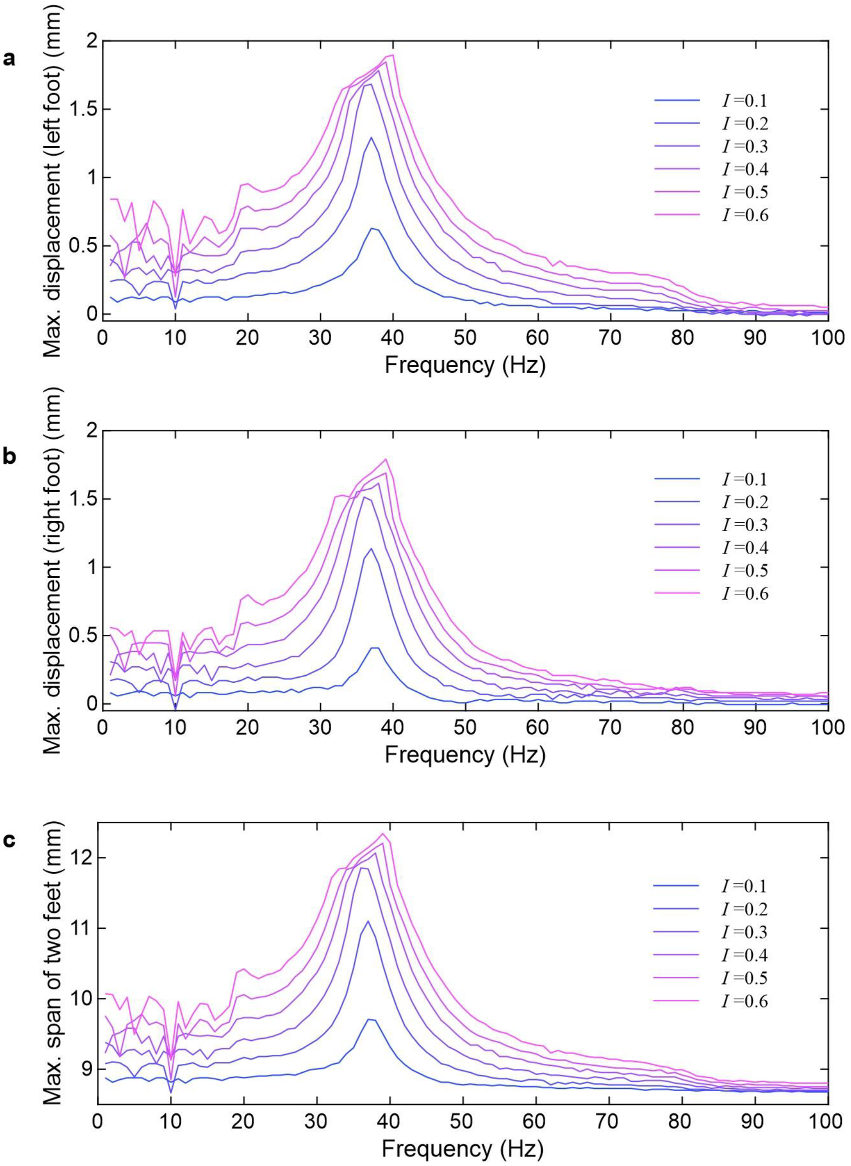

Supplementary Fig. 16 Vibration test of the SEMR TST with sinusoidal currents.

a, Maximum displacement of the left foot to the left versus the applied frequency for different currents. **b**, Maximum displacement of the right foot to the right versus the applied frequency for different currents. **c**, Maximum span between the two feet versus the applied frequency for different currents.

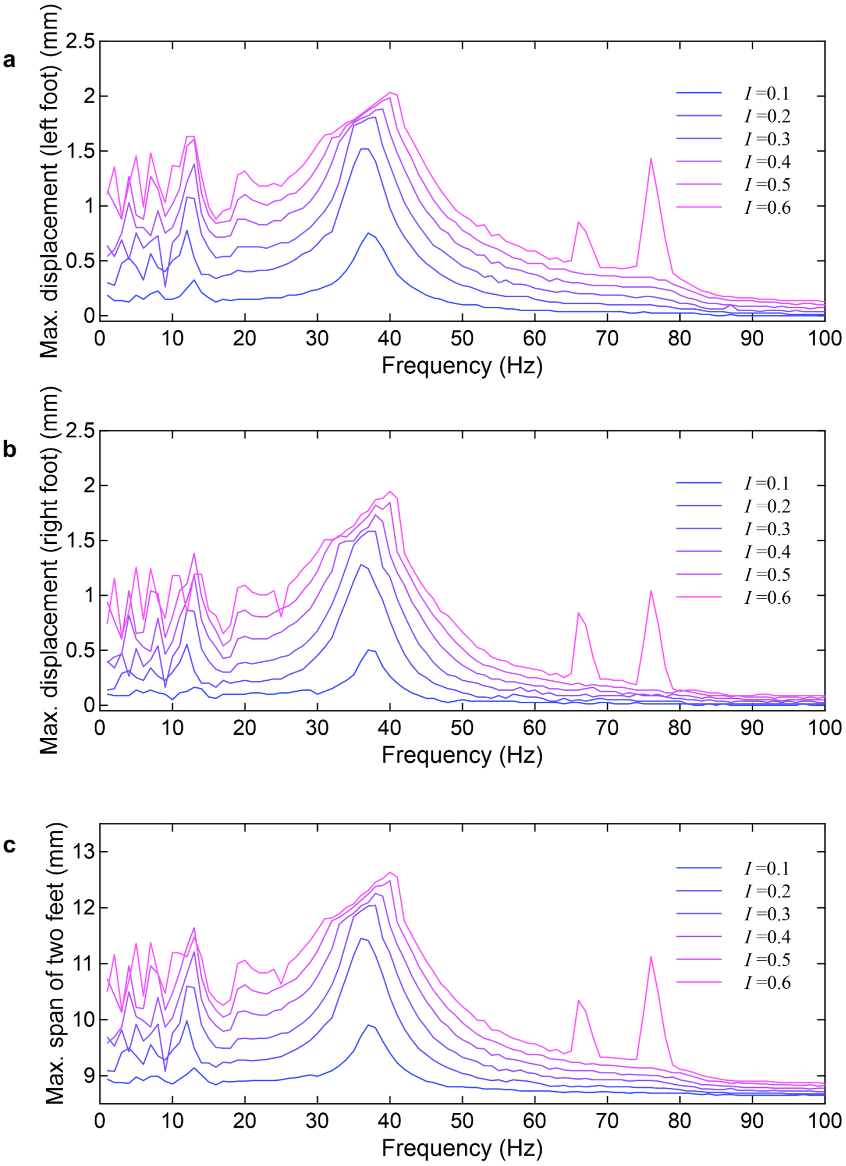

Supplementary Fig. 17. Vibration test of the SEMR TST with square-wave currents.

a, Maximum displacement of the left foot to the left versus the applied frequency for different currents. **b**, Maximum displacement of the right foot to the right versus the applied frequency for different currents. **c**, Maximum span of the two feet versus the applied frequency for different currents.

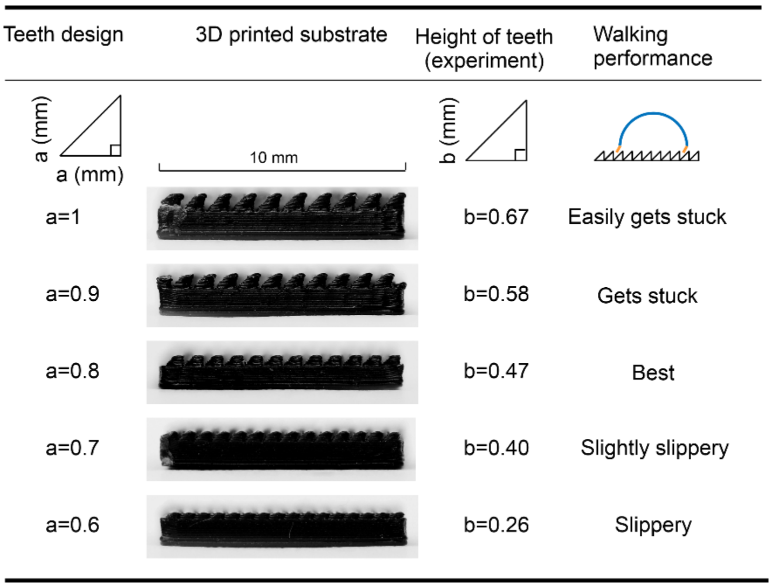

Supplementary Fig. 18. Performance of different 3D-printed substrates.

The dimensions of the designed teeth templates are listed in the left column, and the experimentally measured height of the teeth in the column to the right of the sawtooth image (cross-section of the substrate). The last column describes the subjective performance of a SEMR on various substrates.

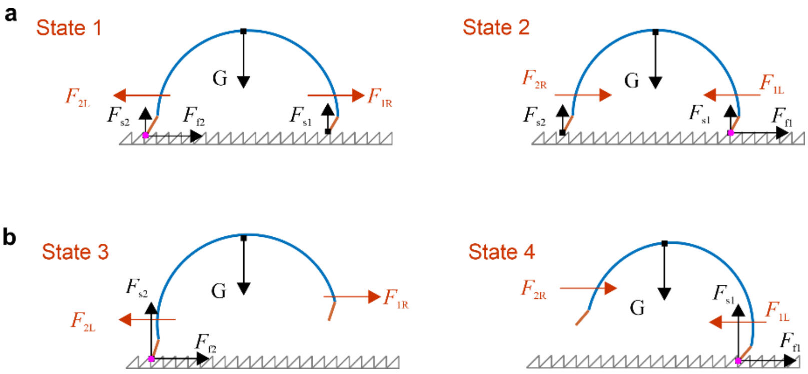

Supplementary Fig. 19 Free body diagram analysis of the robot on the sawtooth-shaped substrate.

The purple dot indicates the mechanical lock point in all panels. The subscripts 1 and 2 refer to the right and left leg or coil, R and L indicate the direction of the Lorentz force, and "s" and "f" denote the normal reaction support and friction forces, respectively, see Fig. 3b in the main text. **a**, A SEMR walks, keeping both feet on the ground. In state 1, the left foot is blocked by the substrate tooth (frictional force $F_{f2} \approx F_{2L}$), while the right foot is unhindered (horizontal reaction force $F_{f1} \approx 0$) and moves forward (rightwards) due to Lorentz force F_{1R}. In state 2, the directions of Lorentz forces are reversed and the right foot is mechanically locked, while the left foot is pulled up to the right by its Lorentz force. **b**, SEMR "hovers over" the ground and oscillates, from time to time pushing and bouncing off the ground with one foot only. In state 3, the left foot is blocked by the substrate tooth (frictional force $F_{f2} \approx F_{2L}$), while the right foot is freely moved forward (rightwards) by the Lorentz force. The left foot's normal reaction support force $F_{s2} \geq G$ is larger than the full gravity force for most of the contact time, providing the acceleration upwards, so that the robot is spring-boarded upwards. In state 4, the directions of Lorentz forces are reversed and the right foot is mechanically locked, while the left foot is pulled up to the right by the Lorentz force. In cases with the opposite directions of the Lorentz forces in either of these pictures, the contact with the ground does not result in a constructive push and is almost inconsequential for the movement of the robot as a whole.

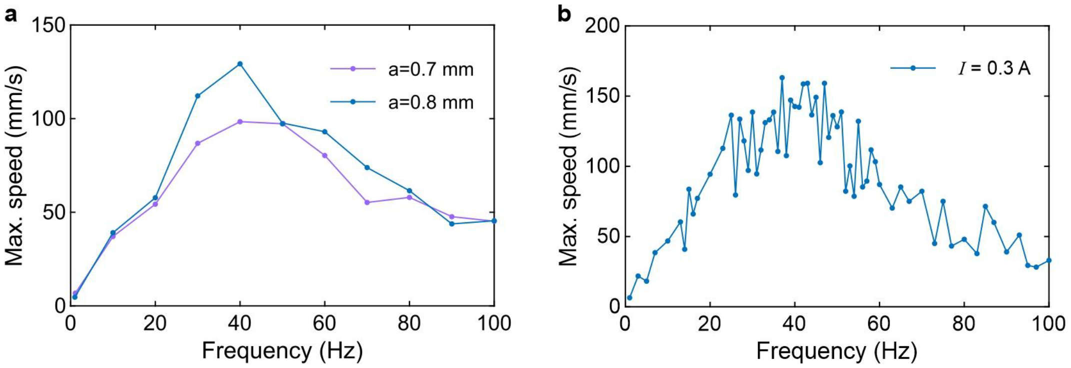

Supplementary Fig. 20. Maximum speed of the SEMR TST.

a, Maximum speed of the SEMR vs. frequency of the driving square-wave current (0.3 A) for two different substrates (teeth heights are *a* =0.7 mm and 0.8 mm). **b**, Maximum speed vs. frequency at a finer frequency resolution for the same applied current of 0.3 A and *a*= 0.8 mm.

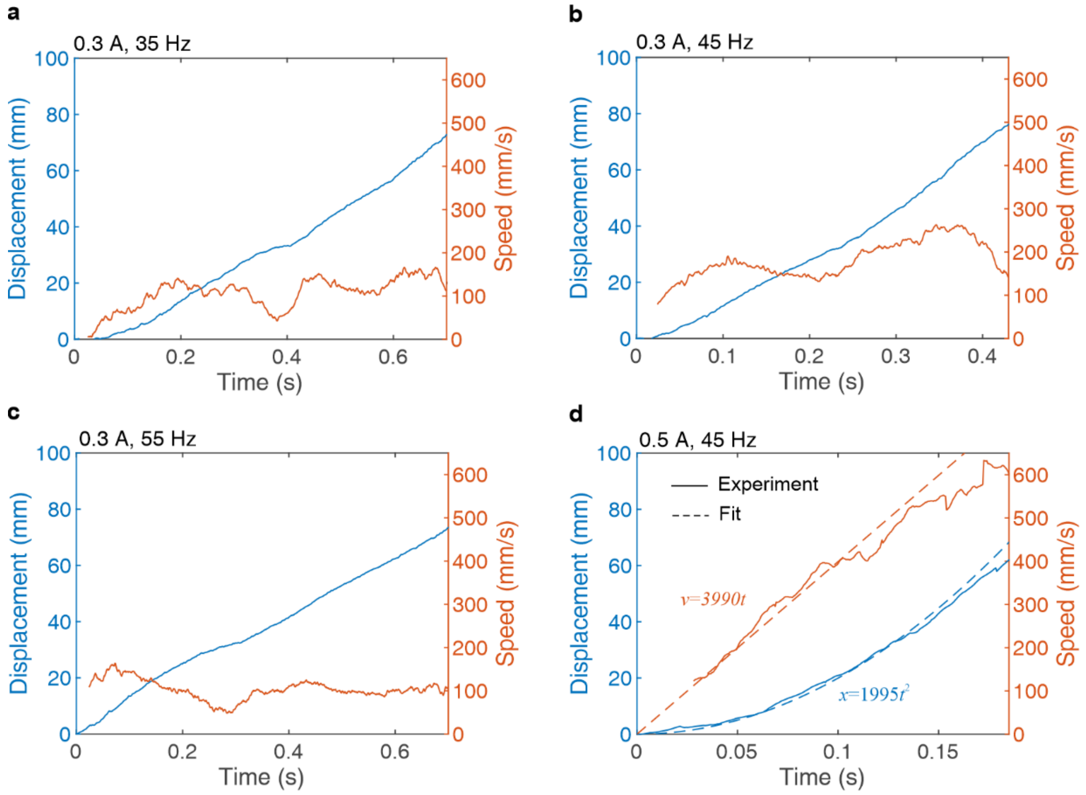

Supplementary Fig. 21. Representative curves of displacements and velocities vs. time of SEMR TST.

Panels **a**, **b**, **c**, and **d**, have different driving currents:(0.3 A, 35 Hz), (0.3 A, 35 Hz), (0.3 A, 35 Hz) and (0.5 A, 45 Hz), respectively. The dashed lines in panel d are obtained by fitting the corresponding data (displacement). The position vs. time curve fits the relation $x = 1995t^2$, which implies constant acceleration with a linear speed increase v=3990t during the entire run. The fitting is conducted using the least-squares method.

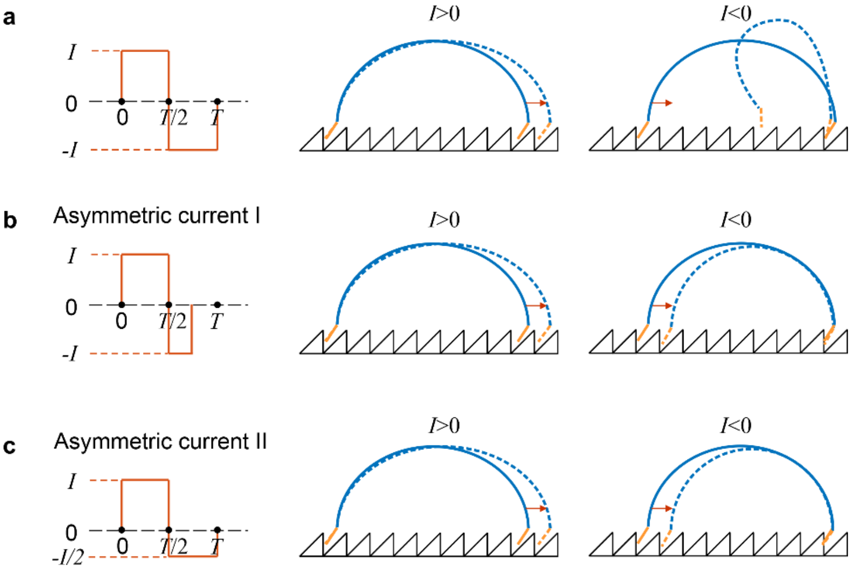

Supplementary Fig. 22. Strategies for stable locomotion at high currents.

a, Schematic deformation of the robot responding to high square-wave current (50% duty cycle, no bias). The dashed contours show the deformed states at the end of the stage with the current direction indicated above. **b**, Schematic deformation of the robot responding to the square-wave current (75% duty cycle, no bias). **c**, Schematic deformation of the robot responding to the square-wave current (50% duty cycle, positive bias).

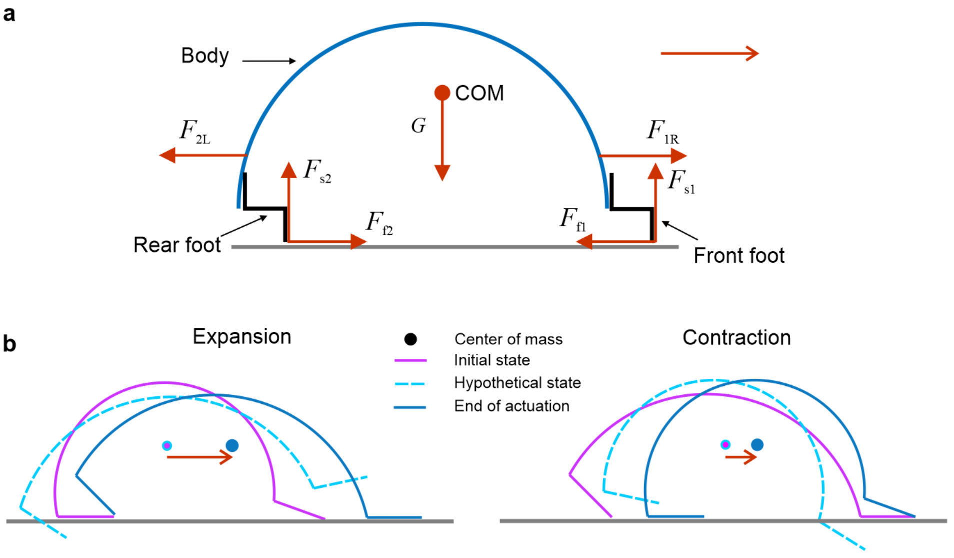

Supplementary Fig. 23. Motion mechanism of the SEMR with L-shaped feet.

a, Free body diagram of the SEMR TL. The center of mass (COM) and various forces are indicated in the plot. **b**, The principle of the walking/running SEMR TL. The key states of SEMR during the expansion and contraction stages (imposed by the periodically alternating directions of both Lorentz forces F_{2L} and F_{1R} shown in the panel a) are indicated with different colors, and the corresponding COM circles have the same color coding. The dashed cyan curves show the expected hypothetical state of the SEMR "without gravity and substrate reaction", the cyan center of mass remains at the initial (magenta) position. However, because the robot feet cannot immerse into the substrate, in the expansion stage (left) the rear foot is pressed towards the ground, while the front one is lifted into the air. As a result, the normal reaction force F_{s2}, and the associated friction force F_{f2} (see panel a) both become much larger for the rear foot, and the robot is propelled forward (to the right), whereas the rear foot contact with the ground serves as a pivot point. The robot position at the end of the expansion actuation phase is shown by the deep blue solid curve. For the contraction stage (right), the sequence is similar, but the roles of the rear and front feet are interchanged (see text for more details). The robot moves forward as a result of cyclic alternation of the expansion and contraction phases.

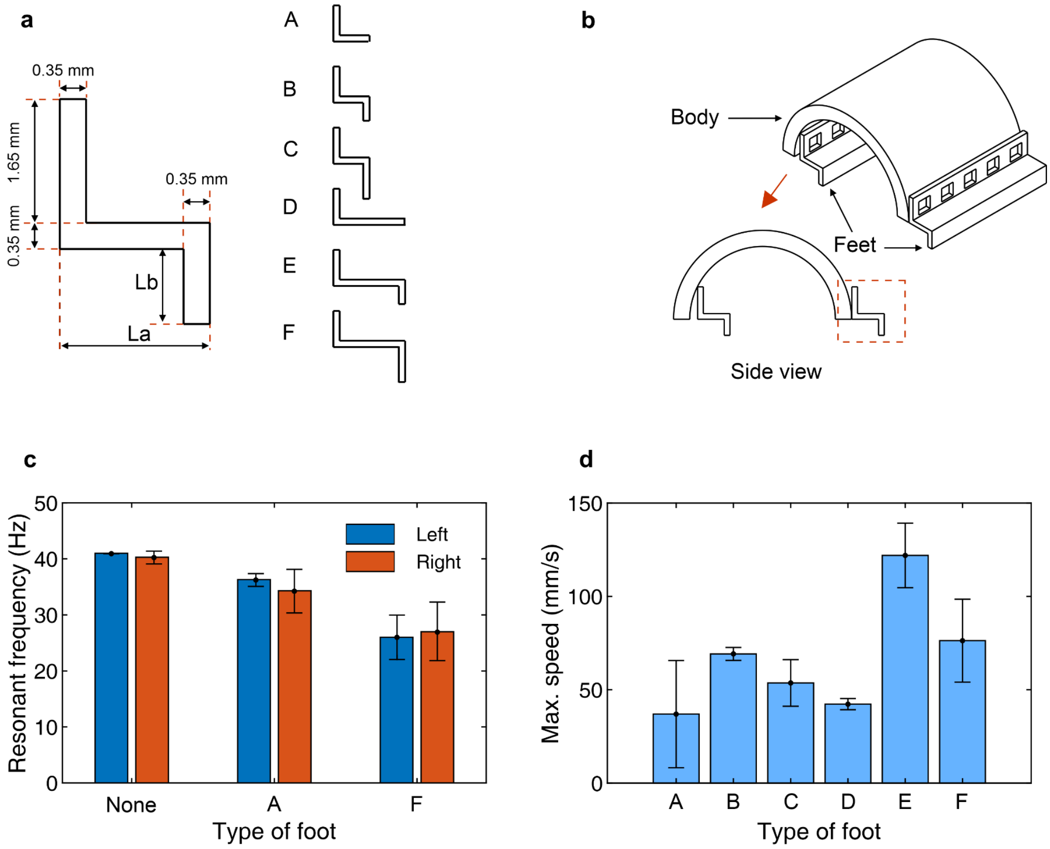

Supplementary Fig. 24. Design and performance of L-shaped feet.

a, Various tested geometries of L-shaped feet (side view). **b**, The assembly of the SEMR with L-shaped feet. **c**, The resonant frequencies of the SEMR clamped in the middle without feet, and with L-shaped feet A and F. The left and right correspond to the position of feet in the side view of panel b. **d**, The maximum running speed of the SEMR with L-shaped feet from A to F subjected to a square-wave current (0.2 A, 20 Hz).

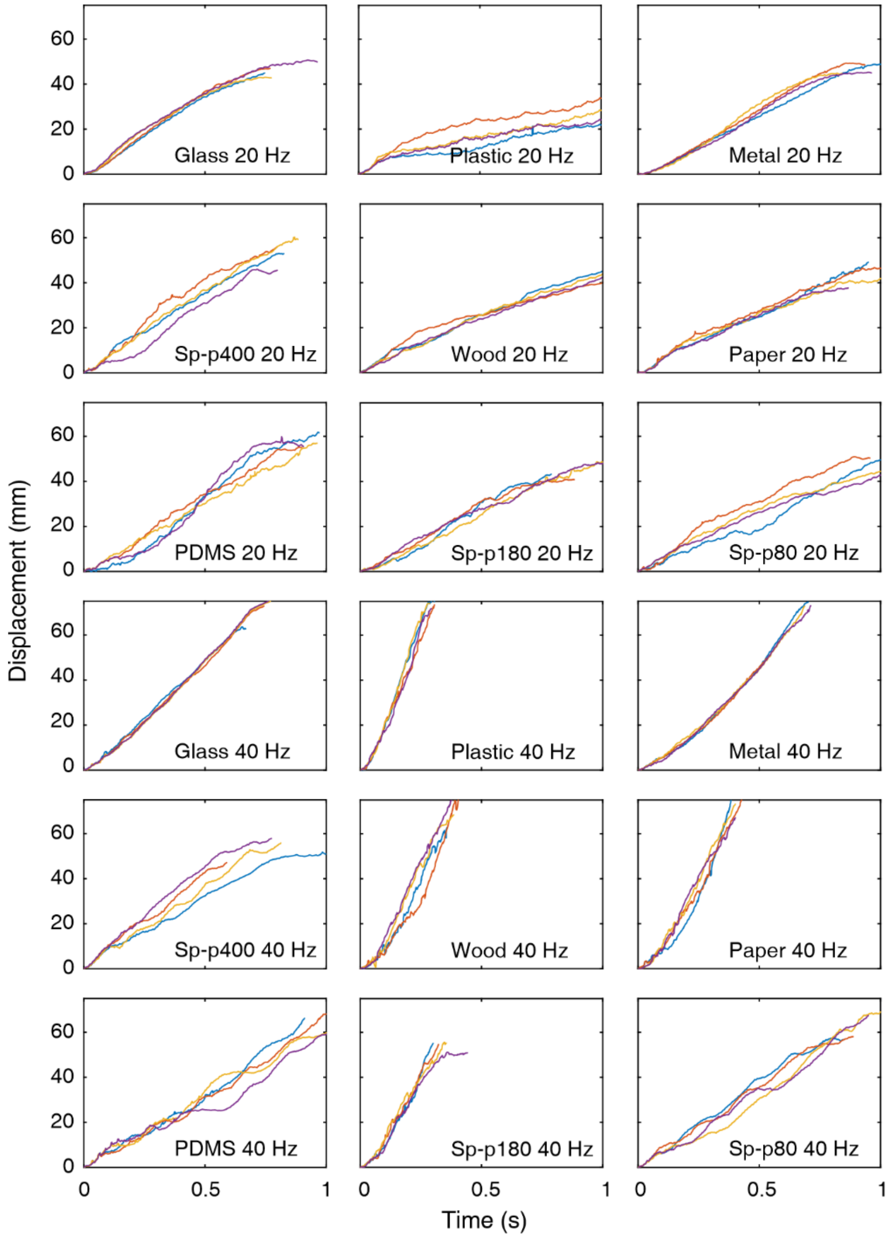

Supplementary Fig. 25. Performance of SEMR TL on various substrates.

Displacement vs. time on different substrates. The materials and frequencies are indicated in the plots. The driven current is 0.3 A for all the cases. Four tests are conducted for each material.

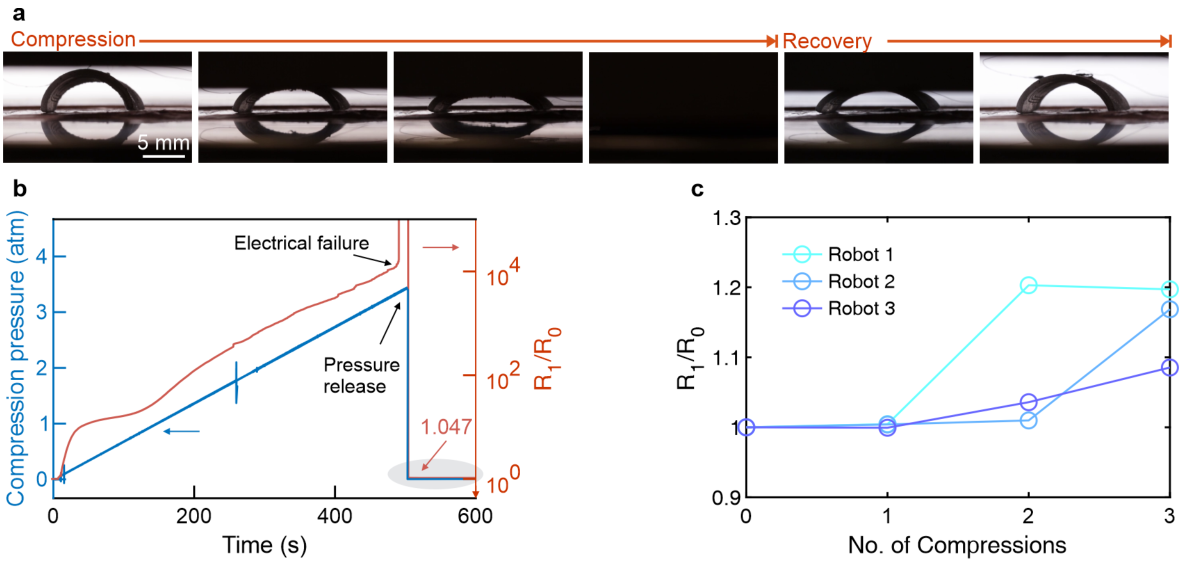

Supplementary Fig. 26. Compression test of the body of SEMR TL.

a, The snapshots of the robot body during compression and recovery (Supplementary Movie 5). **b**, The variation of compression pressure and relative change in robot resistance vs. time. The resistance increases with the increase of compression pressure and recovers to the virtually original value (ratio 1.047) after the release of pressure. **c**, The relative resistance after three compression cycles for three robots. The compression loads are around 50 N.

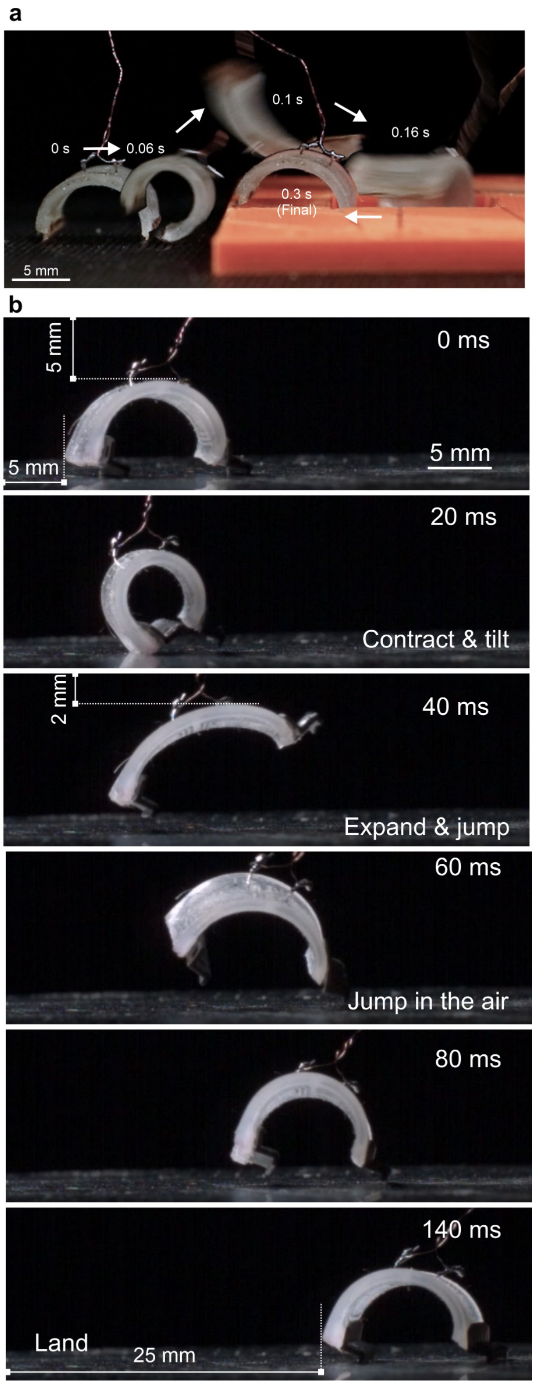

Supplementary Fig. 27. Jumps of SEMR TST and TL.

a, The snapshots of the SEMR TST jumping onto a high stage (3 mm). **b**, The snapshots of the SEMR TL jumping into the air. The maximum jump height and distance of the SEMR TL are 3 mm and 20 mm, respectively. All the above snapshots are from Supplementary Movie 6.

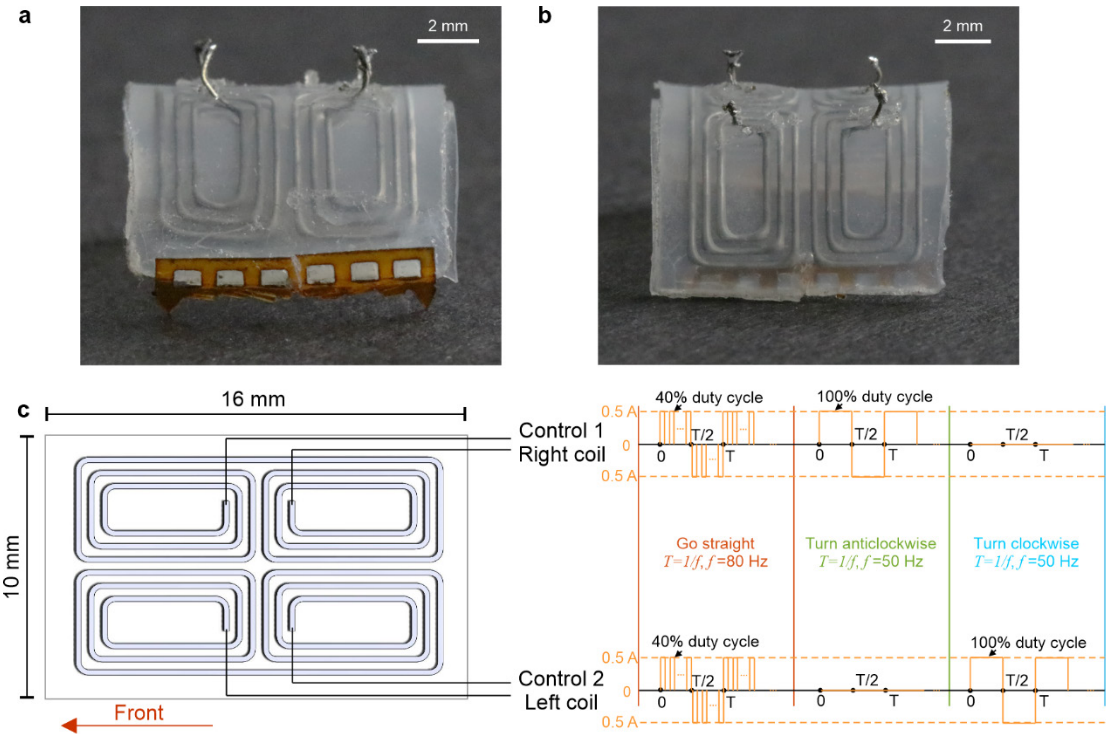

Supplementary Fig. 28. Photo and control schematic of the two-module SEMR TSTS.

a, Front view. **b**, Back view. **c**, Schematic of the two-module SEMR TSTS, and the sequence of the control signals for straight movement, counterclockwise turn, and clockwise turn.

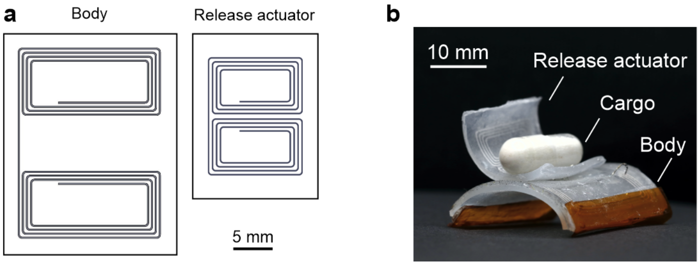

Supplementary Fig. 29. Design and function of transport SEMR TRC.

a, Geometries of the flattened body and release actuator of SEMR TRC. **b**, Photo of SEMR TRC with a cargo (a capsule).

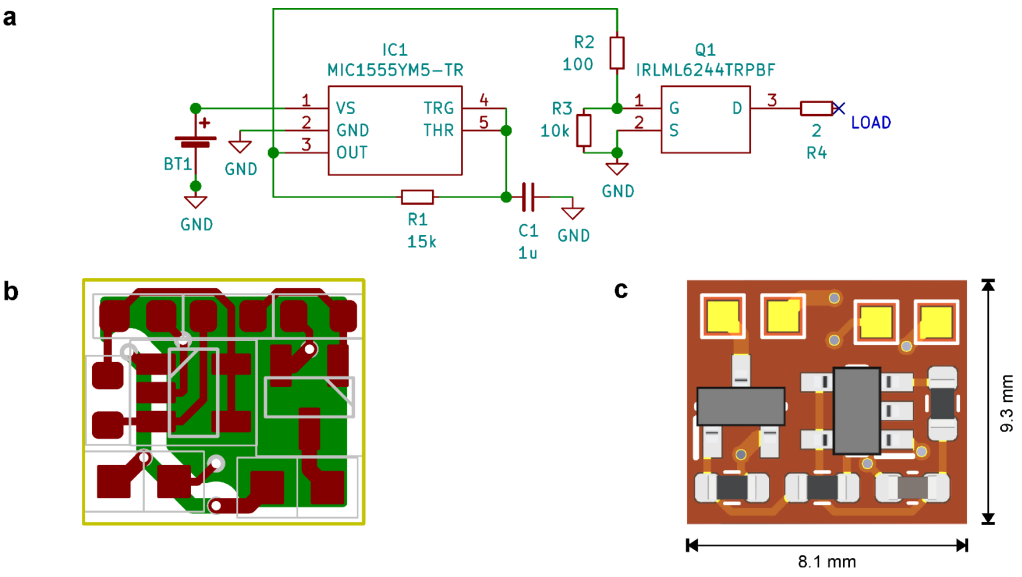

Supplementary Fig. 30. Small PCB used for driving the SEMR UL.

a, Schematic diagram showing all components. **b**, Board layout with indicated traces and footprints. **c,** CAD visualization of the assembled board. These figures have been drawn and generated by the KiCad EDA software (https://www.kicad.org/). The schematic, as well as board symbols, are distributed with the bundled parts library under the license "Creative Commons Attribution-ShareAlike 4.0 International" (https://www.kicad.org/libraries/license/).

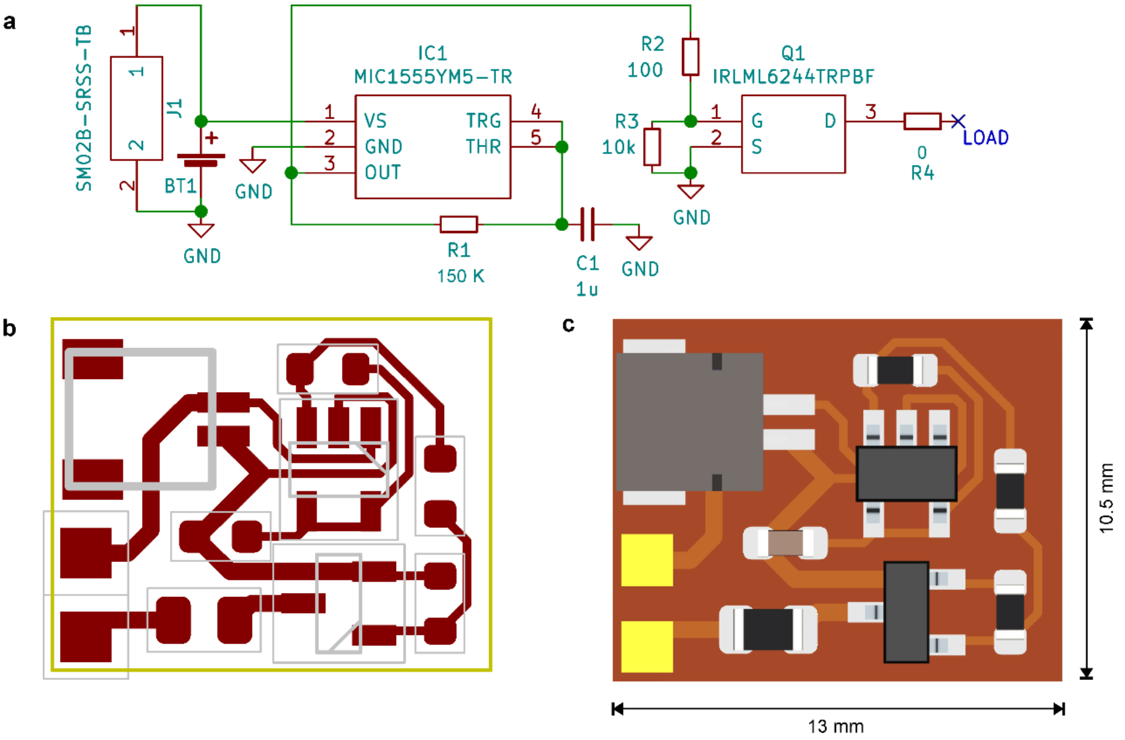

Supplementary Fig. 31. Medium PCB used for driving the SEMR UR1.

a, Schematic diagram showing all components. **b**, Board layout with indicated traces and footprints. **c**, CAD visualization of the assembled board. These figures have been drawn and generated by the KiCad EDA software (https://www.kicad.org/). The schematic, as well as board symbols, are distributed with the bundled parts library under the license "Creative Commons Attribution-ShareAlike 4.0 International" (https://www.kicad.org/libraries/license/).

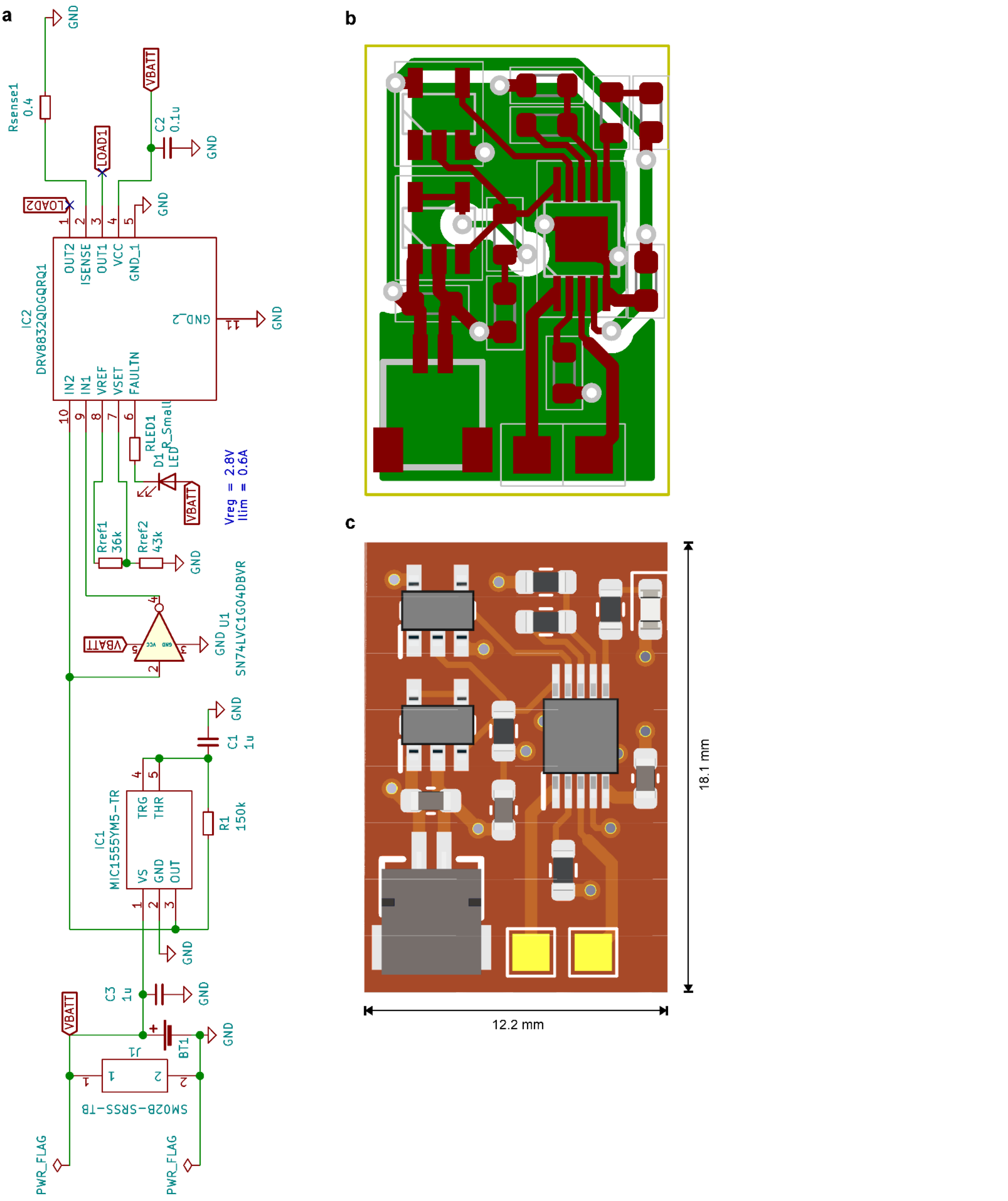

Supplementary Fig. 32. Large PCB used for driving the SEMR UR2.

a, Schematic diagram showing all components. **b**, Board layout with indicated traces and footprints. **c**, CAD visualization of the assembled board. These figures have been drawn and generated by the KiCad EDA software (https://www.kicad.org/). The schematic, as well as board symbols, are distributed with the bundled parts library under the license "Creative Commons Attribution-ShareAlike 4.0 International" (https://www.kicad.org/libraries/license/).

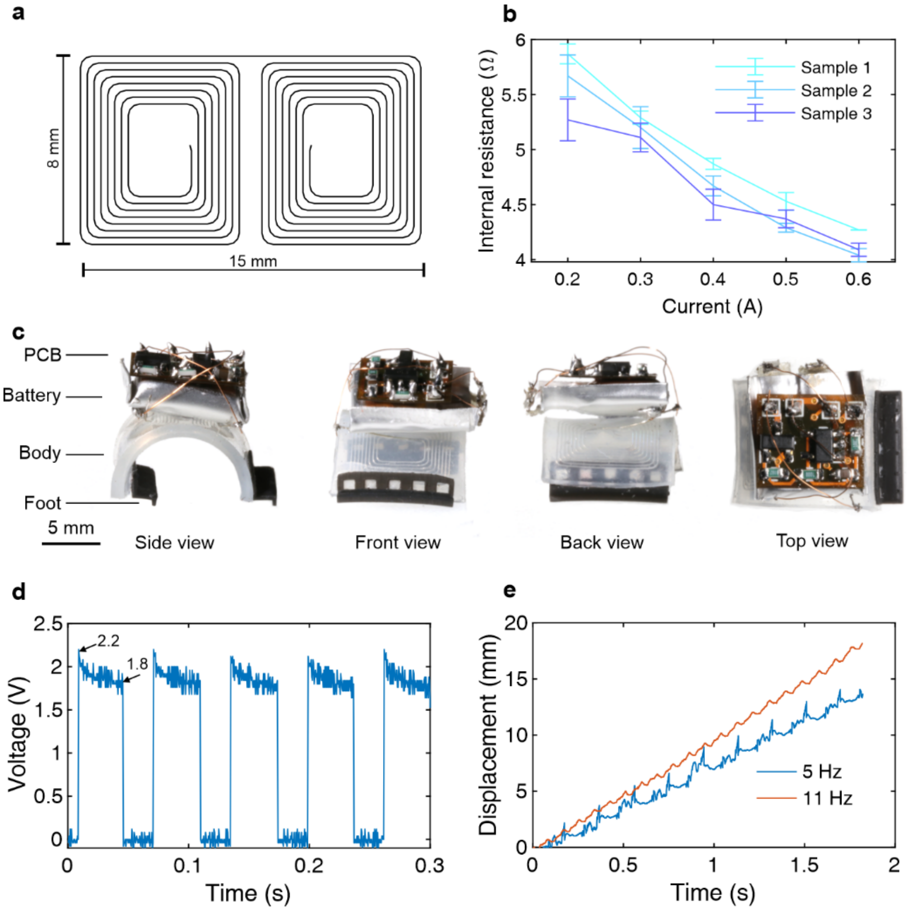

Supplementary Fig. 33. Design and characterization of SEMR UL.

a, Geometries of the LM coil of the SEMR UL. **b**, Internal resistance of the battery as a function of pulse current. The mean (solid curves) and standard deviation (error bars) include three measurements for each data point. **c**, Multiple views of the assembled untethered walking SEMR UL. **d**, Voltage output of the controller powered by the battery. In experiments, this positive signal induced the contraction of the SEMR. **e**, Curves of displacement vs. time of the robot running at a frequency of 5 Hz and 11 Hz.

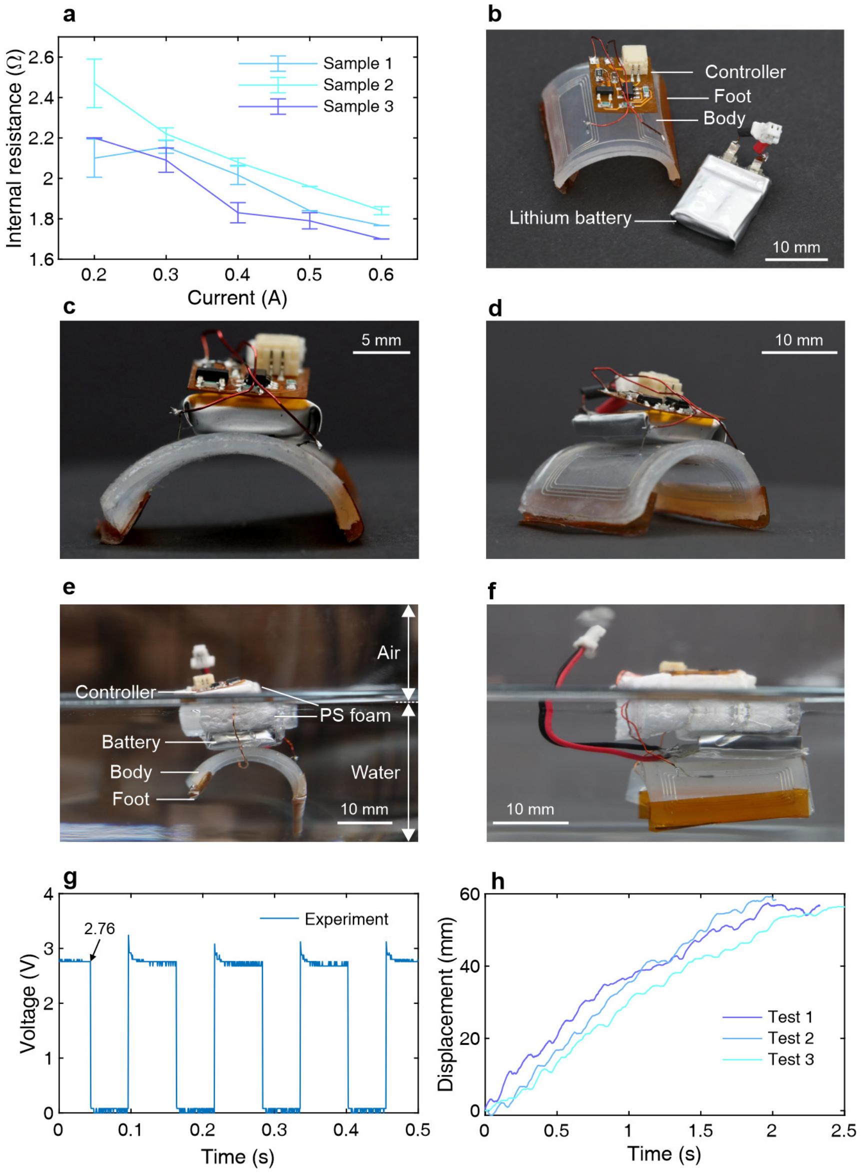

Supplementary Fig. 34. Design and characterization of SEMR UR1.

a, Internal resistance of the battery as a function of pulse current. The mean (solid curves) and standard deviation (error bars) include three measurements for each data point. **b**, Untethered walking SEMR with detached battery and labeled components. **c**, Side view of the assembled, untethered walking SEMR. **d**, Oblique view of the assembled, untethered SEMR. **e**, Untethered swimming SEMR assembled with the labeled components. **f**, Front view of the untethered swimming SEMR floating in the water. **g**, Voltage across the robot coil during the actuation; the front edge peaks exceed the 2.76 V voltage plateau. In experiments, this positive signal induced the contraction of the SEMR. **h**, Displacement vs. time curves of the untethered running SEMR in three different tests.

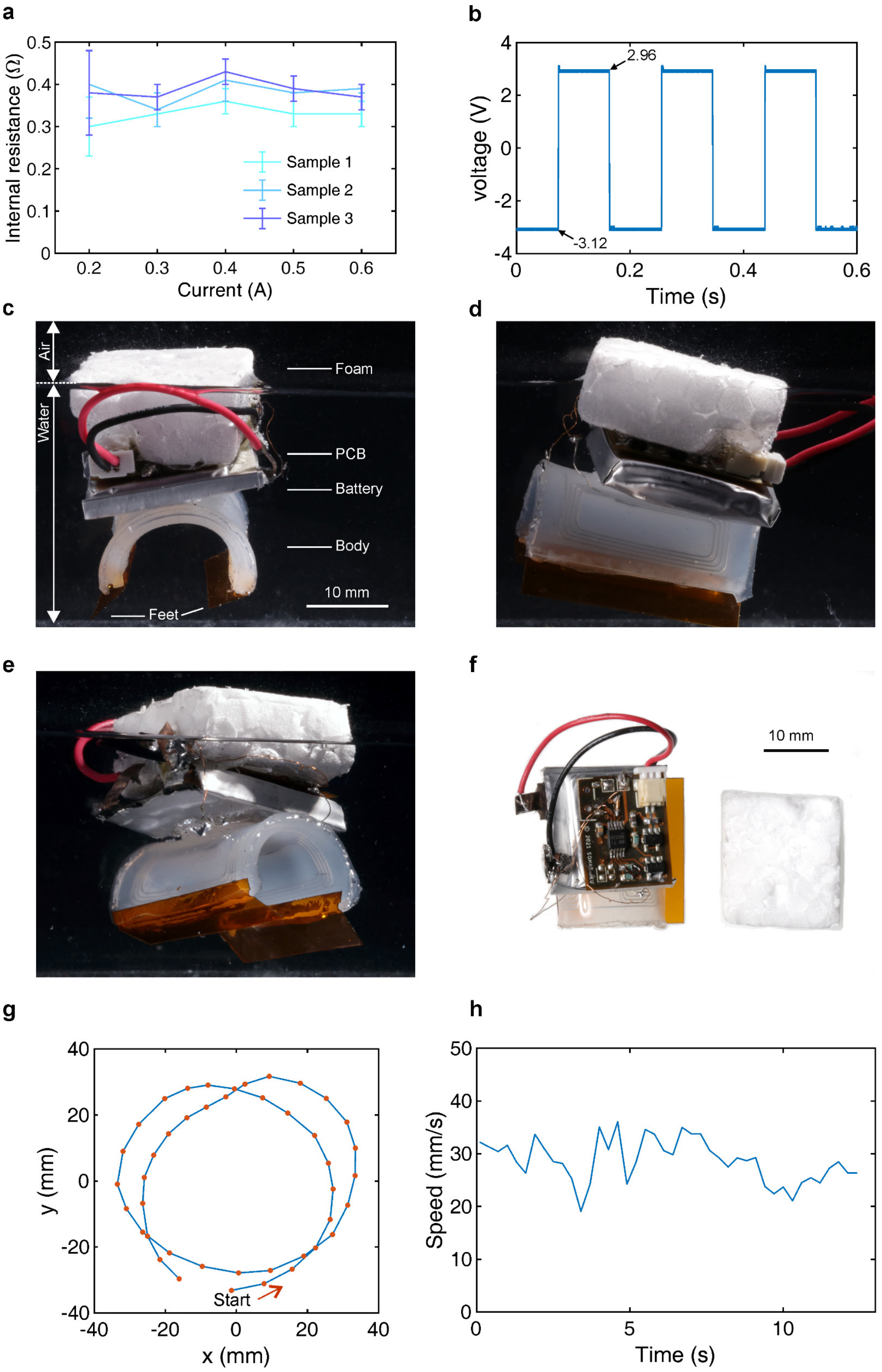

Supplementary Fig. 35 Design and characterization of SEMR UR2.

a, Internal resistance of the battery as a function of pulse current. The mean (solid curves) and standard deviation (error bars) include five measurements for each data point. **b**, Voltage across the robot coil during the actuation. **c-f**, Different views of SEMR UR2. In panels c-e, the robot is floating in the water. **g**, A trace of the untethered swimming SEMR UR2. The time interval between the red dots is 0.34 s. **h**, Speed vs. time curve of the swimming SEMR UR2.

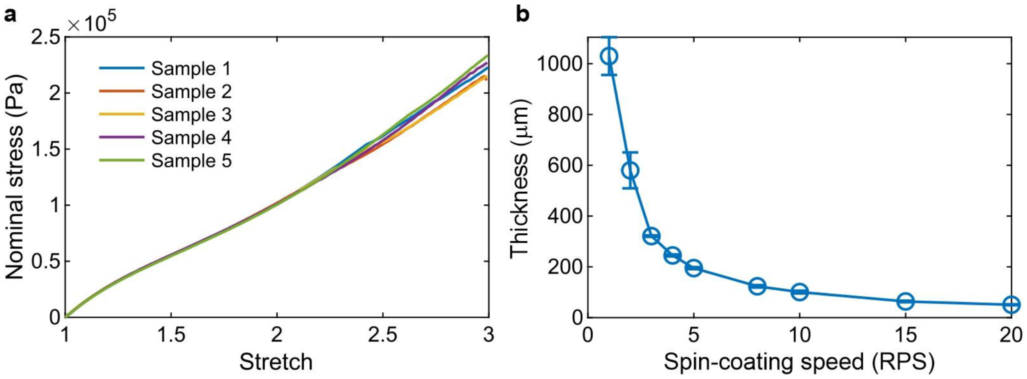

Supplementary Fig. 36. Properties of the elastomer.

a, Uniaxial stretch test of the elastomer. **b**, Thickness of the elastomer film as a function of spin-coating speed. Error bars represent the standard deviation of 3 measurements.

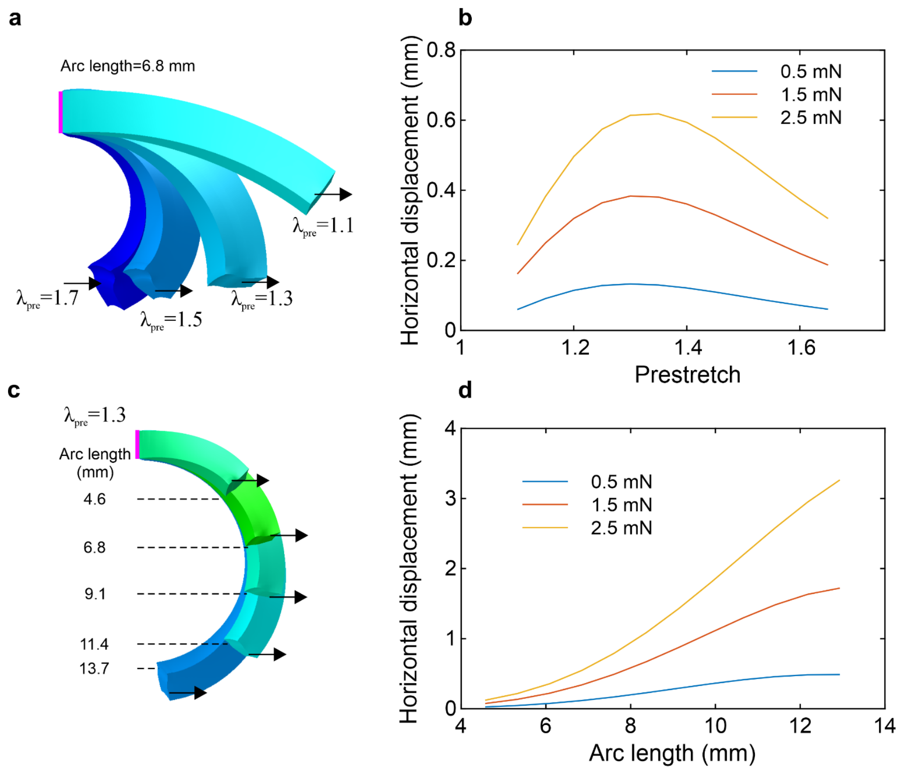

Supplementary Fig. 37. Deformation of curved bilayer films of different designs.

a, Shapes of bilayer films with the same arc length but different prestretches. All bilayer films are clamped at their upper boundary (marked by a thick magenta line). **b**, Horizontal displacement versus different prestretches of the films in panel a subjected to horizontal forces of 0.5 mN, 1.5 mN, and 2.5 mN at their endpoints. **c**, Shapes of bilayer films with the same prestretch $\lambda = 1.3$, but different arc lengths. **d**, Horizontal displacement versus arc length of the bilayer films in panel c subjected to three horizontal endpoint forces, 0.5 mN, 1.5 mN, and 2.5 mN.

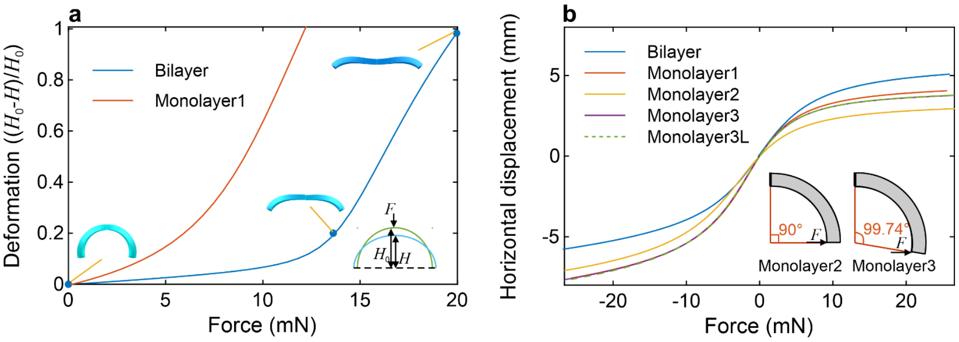

Supplementary Fig. 38. Comparison of deformation of a bilayer and a monolayer film with the same thickness.

a, Relative deflection of two films subjected to vertical force in the middle, as shown in the inset. The contacts between the films and the substrate are frictionless. The thickness of both films is the same (1 mm). The "monolayer 1" film has exactly the same 3D initial geometry as the bilayer film. **b**, Horizontal displacement of the films versus horizontal force, applied at the tip of the film, as shown in the inset. The thicknesses of all films are the same (1 mm). The radius of the film is 4.8 mm. The bilayer film includes initially 350 µm thick layer with a prestretch of 1.3 (details in the text). The "monolayer 2" film is similar to a "monolayer1", with the arc angle exactly equal to a quarter circle. The "monolayer 3" is also "planar", but longer than the "monolayer 2" (shown in the inset). The "monolayer 3L" has the same geometry as "monolayer 3", but uses a linear material model with the same shear modulus.

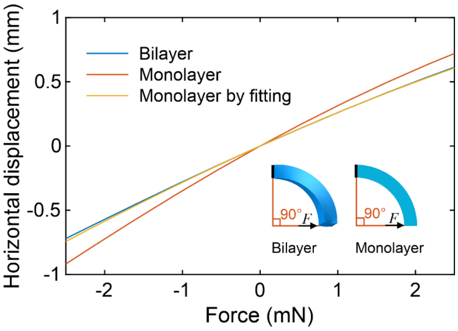

Supplementary Fig. 39. Effective modulus of the equivalent curved monolayer film.

The geometries of the unloaded bilayer and monolayer films are the same and the arc angle of both films is 90°, as shown in the inset. When the shear moduli of the bilayer and monolayer films are the same, the numerically simulated monolayer curve (red) is softer (has larger displacement) than a bilayer (blue). Monolayer and bilayer curves coincide in the linear range, if the fitted monolayer film (yellow) has a modulus 1.23 times larger than the bilayer.

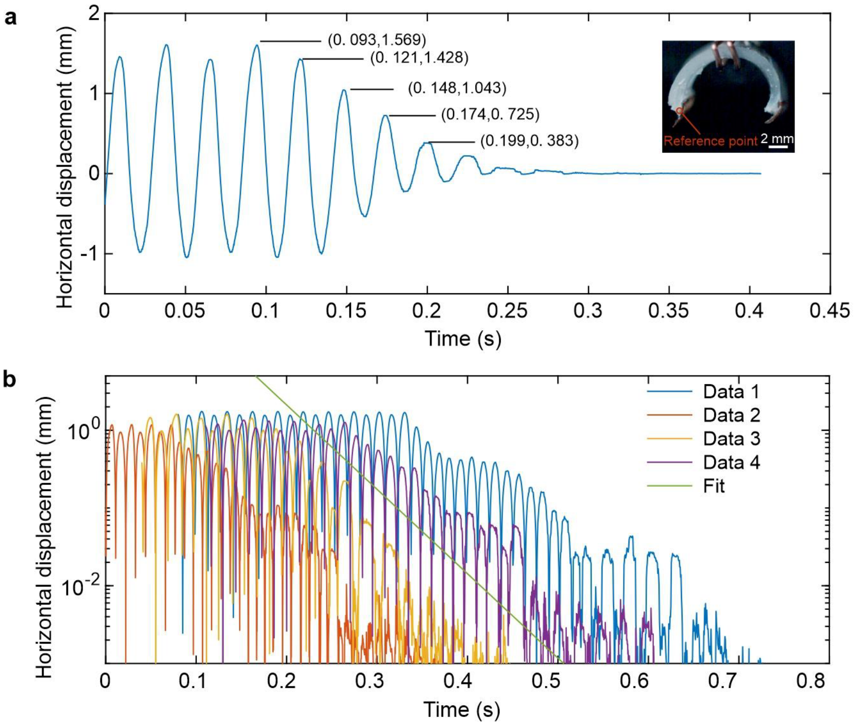

Supplementary Fig. 40. Free oscillation decay test for the SEMR.

a, The experimental setup is the same as in Supplementary Fig. 15c. The SEMR is driven by a square-wave current (0.2 A, 37 Hz) until a stationary vibration amplitude is reached; then the power is turned off at the time $t = 0.093\,\mathrm{s}$. The displacement of the left foot of the SEMR is determined from the video analysis, which tracks the position of the reference point (shown in the inset). The time and displacement are indicated near five maxima of the displacement curve. **b**, Log-plot of the absolute value of the deflection $|x|$ for several free decay runs, similar to the one shown in panel a. The decay starts at different moments, shifted by about 0.1 s. The straight line shows an exponential amplitude decay $\sim e^{-\gamma t/2}$ with $\gamma = 50\,\mathrm{s}^{-1}$, which roughly corresponds to the average decay rate for all 4 curves.

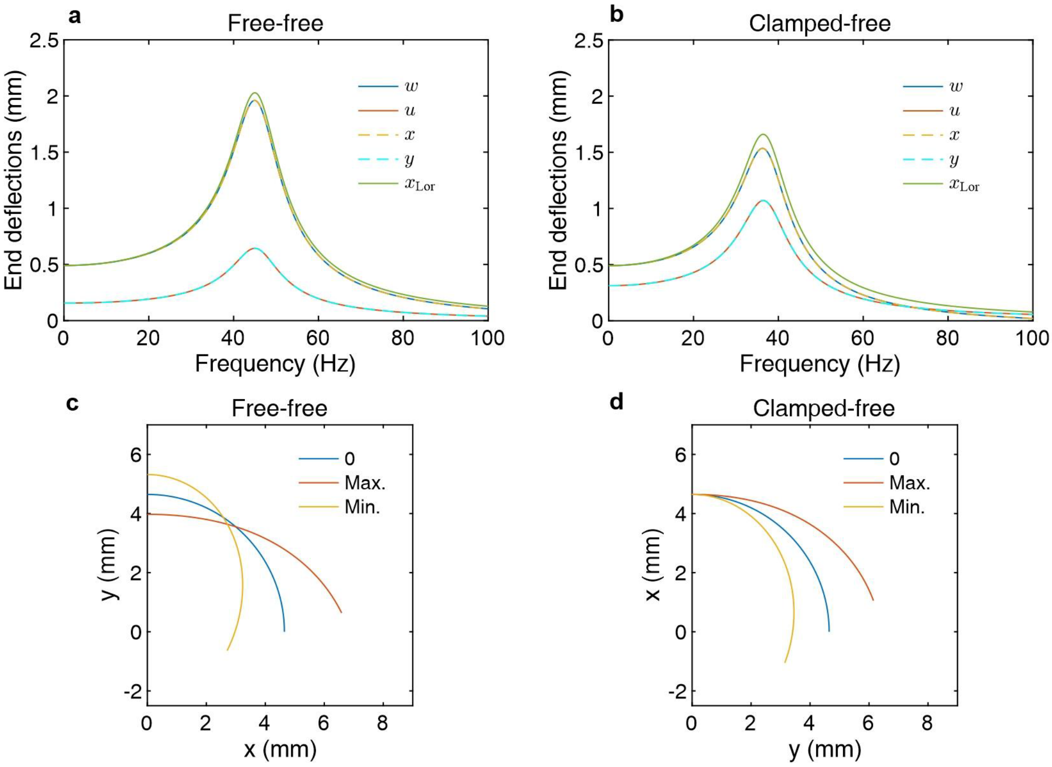

Supplementary Fig. 41. Resonance curves for the curved-beam robot with free-free and clamped-free boundary conditions.

a, Frequency-dependent amplitudes of one-leg radial *w*, tangential *u*, and *x*- and *y*- end deflections. For the half-circular robot, with $\psi = \pi / 2$, for *end* displacements $w \equiv x$, and $u \equiv y$. The olive x_{Lor} curve shows the Lorentzian approximation for *x*-deflection, as described in the text. **b**, The same as in panel a, but for the robot clamped in the middle, as was used in the resonance experiments. Resonant frequency and deflection are smaller for the clamped case, although the static deflection is the same for $\psi = \pi / 2$. The ratio of resonant frequencies is $f_{\mathrm{ff}} / f_{\mathrm{cf}} = 1.226$, which is very close to the value $f_{\mathrm{ff}} / f_{\mathrm{cf}} = 45/37 = 1.216$ observed in the experiments. **c**, Maximum deflections of a free robot at resonance. Blue 0 - neutral robot shape, red Max. - outward deflection, yellow Min. - inward deflection. **d**, The same as in panel c, but for the clamped-free robot.

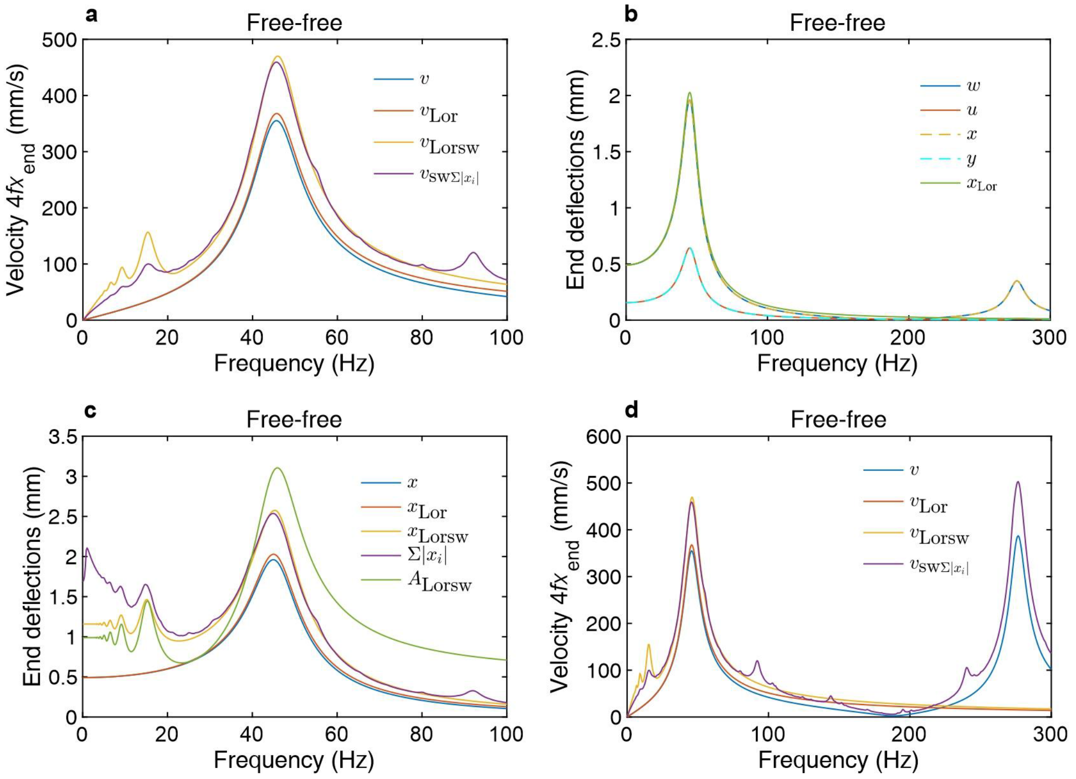

Supplementary Fig. 42. Square-wave response, higher vibration modes, and running speed of the robot.

a, Velocity in x-direction as a function of frequency. The curve v is calculated as $4fx(f)$ with $x(f)$ from Supplementary Fig. 41a, and the curve v_{Lor} in the same way, using the Lorentzian approximation there. The curves v_{Lorsw} and the curve $v_{\mathrm{sw}\sum|x_i|}$ refer to the SW excitation of the same *amplitude*. For the same *power,* the SW response is reduced by a $\sqrt{2}$ and lies slightly below the harmonic curve. The curve v_{Lorsw} is calculated from the Lorentzian SW formula (48), while the curve $v_{\mathrm{sw}\sum|x_i|}$ is an upper limit from the exact formulas. The minor maxima at low frequencies $f = f_0/(2n+1)$ are due to the resonance of the higher harmonics of the SW with the main frequency f_0. Small maxima at higher frequencies $f = f_1/(2n+1)$ are due to similar resonance with the second (symmetric) mode f_1, which can be seen in panel b. They are not present in the Lorentzian approximation, which may also exceed the exact theoretical limit, because it overestimates the spectral response *between* the major resonances (see panel d). **b**, The same as in Supplementary Fig. 41a, but for a larger frequency range. *w-, u-, x-* and *y-* curves show the next (symmetric) mode, which is absent in the Lorentzian, based on the lowest mode. **c,** Different x-amplitudes. Blue x – true amplitude, red x_{Lor} – Lorentzian approximation, yellow x_{Lorsw} – exact Lorentzian SW expression (43), purple $\sum|x_i|$ – exact theoretical upper limit (sum of harmonic amplitudes, details in the text), green A_{Lorsw} – Lorentzian amplitude of free oscillations in SW excitations (Eq. (37)). SW yields much larger values at lower frequencies, as it contains high harmonics. **d**, The same as in panel a, but for a larger frequency range. The velocity for the second mode is about the same as for the main one, because it has a much higher frequency. This high speed is not realized in practice (for the used forces and amplitudes), because finite groove depth and size require sufficient amplitudes to start the movement.

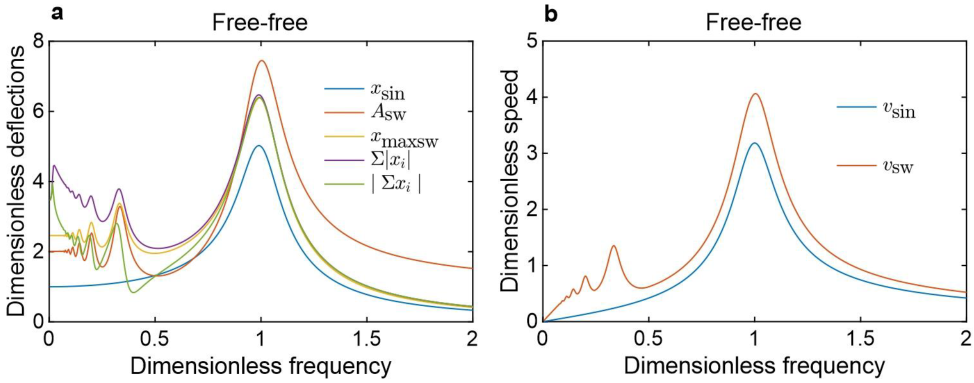

Supplementary Fig. 43. Square-wave and sinusoidal responses of a Lorentzian harmonic oscillator.

a, Dimensionless deflection $xm\omega_0^2 / F$, vs. dimensionless frequency ω / ω_0, similar to Supplementary Fig. 42c for $\gamma = 0.2\omega_0$. Blue $x_{\rm sin}$ – sinusoidal response amplitude, red $A_{\rm Lor}$ – Lorentzian amplitude of free oscillations in SW excitations (Eq. (37)), yellow $x_{\rm maxsw}$ – exact Lorentzian SW expression (43) for the maximum deviations, purple $\sum|x_i|$ – theoretical upper limit (sum of Lorentzian harmonic amplitudes), olive $\left|\sum x_i\right|$ – amplitude of harmonic amplitudes with phases (details for all curves are given in the text). SW yields much larger values at lower frequencies, as it contains high harmonics. Low-frequency resonances are due to in-phase switches between two equilibria, which can be seen in Supplementary Fig. 44. **b**, Dimensionless velocity $vm\omega_0 / F$, similar to Supplementary Fig. 42a, d. Blue $v_{\rm sin}$ – sinusoidal case, calculated as $4fx_{\rm sin}(\omega)$ from panel a, red $v_{\rm sw}$ – SW case using exact Lorentzian expression (48). At low frequencies, both curves show linear increases at low frequencies, and the SW curve shows secondary resonances there.

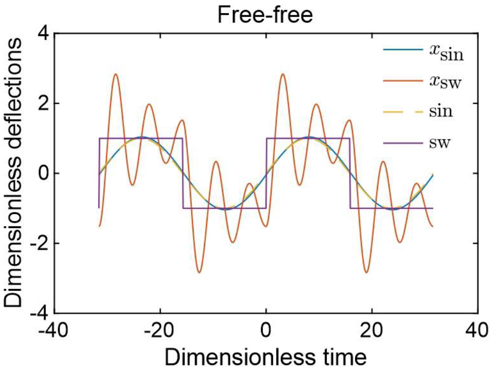

Supplementary Fig. 44. Comparison of temporal responses to a sinusoidal and SW excitation for $\omega = \omega_1 / 5$, where one of the low-frequency resonances lies.

Yellow dash sin – sinusoidal excitation, blue x_{sin} – system response to it, purple sw – SW excitation, red x_{sw} - system response to it, deflections are normalized as in Supplementary Fig. 43a. Sinusoidal response at low frequencies in dimensionless units is almost equal to the excitation itself (the blue x_{sin} curve in Supplementary Fig. 43a $x_{\mathrm{sin}}(\omega / \omega_0 \ll 1) \approx 1$ in this region.) For the SW excitation, the system oscillates around two alternating equilibria, as described in the text. As the frequency of the free oscillations $\omega_1 = 5\omega$, and the damping is moderate, the system shows appreciable resonant deflection (about 3 in dimensionless units) after each sharp SW step-front.

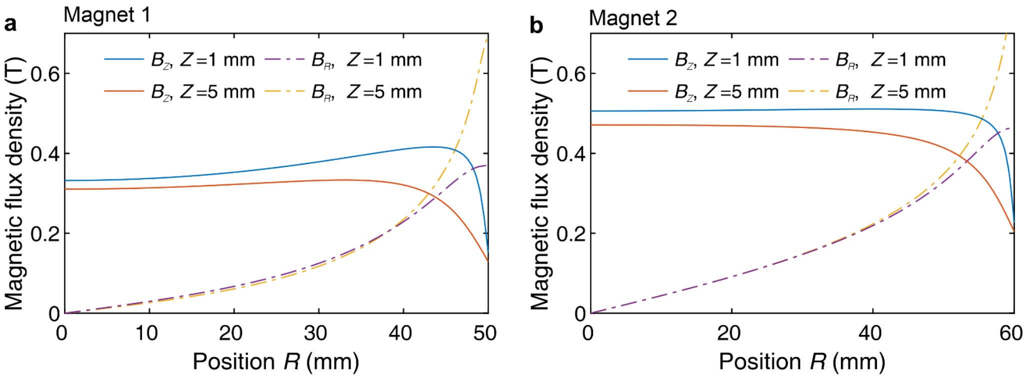

Supplementary Fig. 45. Magnetic fields near the surfaces of two magnets.

The position R refers to the central axis of symmetry of the plate magnets. B_Z and B_R are two components of magnetic field which are perpendicular to the central axis of symmetry of the plate magnets, respectively. **a**, and **b**, are for magnet 1 and magnet 2, respectively.

3. Supplementary Tables

Supplementary Table 1. Comparison of the maximum running speeds of insects, mammals, rigid/flexible robots and soft robots.

Category$^{\alpha}$	Max. relative speed (BL/s)	Body length (mm)	Max. speed (mm/s)	Weight (mg)	Feet	Power$^{\beta}$	Ref.
**Centipede (*Scolopendra heros*)**	1.5	1.52E+02	2.3E+02	1.16E+04	/	/	[11]
**Ant (*Cataglyphis fortis*)**	33.3	1.50E+01	5.0E+02	8.36E+00	/	/	[12]
**American cockroach (*Periplaneta Americana*)**	50.0	3.00E+01	1.5E+03	8.30E+02	/	/	[13]
**Spider (*Schizocosa ocreata*)**	69.8	8.89E+00	6.2E+02	5.00E+01	/	/	[14,15]
**Mite (*Paratarsotomus macropalpis*)**	323.0	1.00E+00	3.2E+02	1.07E-01	/	/	[16]
**Elephant**	1.1	6.00E+03	6.8E+03	4.00E+09	/	/	[17,18]
**Human**	5.1	2.00E+03	1.0E+04	7.00E+07	/	/	[19]
**Horse**	9.8	2.00E+03	2.0E+04	6.80E+08	/	/	[19]
**Cheetah**	24.1	1.30E+03	3.1E+04	5.00E+07	/	/	[19]
**Dog**	28.7	7.00E+02	2.0E+04	3.00E+07	/	/	[20]
**Mouse**	35.7	1.00E+02	3.6E+03	4.00E+04	/	/	[21]
**Cat**	53.6	2.50E+02	1.3E+04	4.00E+06	/	/	[22]
**Piezo 1**	8.7	3.82E+01	3.3E+02	2.17E+03	Elastic fin film	T	[23]
**Piezo 2**	10.1	4.36E+01	4.4E+02	1.27E+03	Rigid plastic tip	T	[24]
**Magnetic polymer (MP)/magnetic field**	14.9	2.50E+00	3.7E+01	1.00E+00	Rotational leg/polymer	U	[25]
**DC motor (DCM) 1**	15.0	1.00E+02	1.5E+03	1.62E+04	Rotational leg/composite polymer	U	[26]
**Polyvinylidene difluoride (PVDF)$^{\gamma}$**	20.0	1.00E+01	2.0E+02	2.40E+01	Rigid plate	T	[27]
**DCM 2**	27.0	1.00E+02	2.7E+03	3.00E+04	Composite polymer	U	[28]
**DCM 3**	47.1	1.04E+02	4.9E+03	5.40E+04	Composite polymer	U	[29]
**Dielectric elastomer (DE) 1**	1.0	2.20E+01	2.3E+01	2.00E+02	Hook	T	[30]
**SEMR UL**	**1.2**	**9.00E+00**	**1.1E+1**	**7.06E+02**	**3D printed plastic film**	**U**	**This work**
**SEMR UR1**	**2.1**	**2.00E+01**	**4.2E+01**	**2.18E+03**	**Rectangular plastic film**	**U**	**This work**
**Soft magnetic elastomer (SME) 1**	2.6	1.75E+00	4.6E+00	5.00E+00	Elastomer	U	[31]
**Pneumatic**	2.7	7.00E+01	1.9E+02	4.50E+04	Hybrid (plastic and rubber)	T	[32]
**DE 2**	4.0	4.00E+01	1.6E+02	4.90E+03	Hook	T	[33]
**SEMR TL**	**35.0**	**9.00E+00**	**3.2E+01**	**1.91E+02**	**3D printed plastic film**	**T**	**This work**
**SEMR TST**	**70.0**	**9.00E+00**	**6.3E+01**	**1.72E+02**	**Sawtooth-shaped plastic film**	**T**	**This work**

$^{\alpha}$Some of the body dimensions for insects (purple) and mammals (green), are not listed in the references, and are taken from the internet (e.g., wikipedia.org). In some references, the body length of the animal, etc., is listed as a range of values. In such cases the average value is taken for comparison. Hard and soft robots are indicated with colors, black and blue, respectively. We use engineering notations. This means that 'M E N' denotes '$M\times10^{N}$'.
$^{\beta}$'T' and 'U' refer to 'Tethered' and 'Untethered', respectively.
$^{\gamma}$We consider this to be a flexible robot. It is made of rigid material, but has a flexible structure.

Supplementary Table 2. Comparison of the maximum swimming speeds of animals and robots.

Category		Max. relative speed (BL/s)	Body length (mm)	Max. speed (mm/s)	Weight (mg)	Ref.
**Nature creatures**	Blue Whale (*Balaenoptera musculus*)	0.4	2.60E+04[III]	1.0E+04	1.0E+11[*]	34
	Crocodile (*Crocodylus porosus*)	0.5	1.00E+03	5.4E+02	1.0E+09[*]	35
	Human (*Homo sapiens*)	1.0	2.0E+03	2.0E+03	6.0E+07[*]	36
	Killer whale (*Orcinus orca*)	1.7	4.74E+03	7.9E+03	6.0E+09[*]	35
	Bottlenose Dolphin (*Tursiops truncatus*)	2.3	2.61E+03	6.0E+03	3.0E+08[*]	35
	Salmon (*Salmo salar*)	6.4	7.50E+02	4.8E+03	4.0E+06[*]	37
	Pike (*Esox lucius*)	12.7	1.65E+02	2.1E+03	2.0E+07[*]	38
	Swordfish (*Xiphias gladius*)	17.8	2.03E+03	3.6E+04	6.0E+08[*]	39
	Tadpole (*Xenopus laevis*)	17.9	2.80E+01	5.0E+02	9.10E+00	40
	Water strider (*Hydrometra*)	136.4	1.10E+01	1.5E+03	1.00E+01	41
**Robots[+]**	Ionic polymer–metal composites (IPMCs)	0.2	9.60E+01	2.4E+01	1.62E+04	42
	Piezo 3[†]	0.3	1.00E+02	3.0E+01	1.00E+03	43
	SME 2[I]	0.5	1.00E+01	5.0E+00	2.00E+00	44
	DE 3[II]	0.7	9.28E+01	6.4E+01	9.03E+04	45
	Piezo 4	0.8	6.00E+01	4.5E+01	1.50E+04	46
	Shape memory alloy (SMA)[II]	0.8	1.46E+02	1.1E+02	3.00E+04	47
	Pneumatic	0.8	1.50E+02	1.2E+02	5.10E+04	32
	DCM 4	1.0	2.66E+02	2.6E+02	3.80E+05	48
	DCM 5 [II]	1.2	4.40E+02	5.3E+02	1.30E+06	49
	**SEMR UR1[II]**	**1.25**	**2.0E+01**	**2.5E+01**	**2.20E+03**	**This work**
	**SEMR UR2[II]**	**1.80**	**2.0E+01**	**3.6E+01**	**4.73E+03**	**This work**
	DCM 6 [II]	4.0	2.55E+02	1.0E+03	3.06E+05	50
	**SEMR TST[†]**	**4.8**	**9.00E+00**	**4.3E+01**	**1.80E+02**	**This work**
	SME	17.0	5.90E+00	1.0E+02	2.40E+00[*]	51

*: The weight is not provided directly in the literature and is taken from the internet (mainly from wikipedia.org). Some average number is chosen, or the weight is estimated from the reference.
+: Robots driven by propellers are not included.
†: The body of the robot is mainly on the water surface.
I: Untethered robot powered by an external magnetic field.
II: Untethered robot powered by an internal battery.
III: We use engineering notations, which means that ‘M E N’ indicates ‘$M\times10^{N}$’.

Supplementary Table 3. Parameters of 3D printed feet

Type / Item*	A	B	C	D	E	F
La (mm)	1	1	1	2	2	2
Lb (mm)	0	0.5	1	0	0.5	1
Weight (mg)	10.3±0.1	12.0±0.3	14.3±0.1	16.4±0.4	18.7±0.1	21.0±0.3

*Six feet are measured for each type. La and Lb are designed values.

Supplementary Table 4. Parameters of the non-magnetic battery

Item* / Battery	Product number	Capacity (mAh)	Dimension (mm)	Weight (g)	Internal resistance (Ω)	Max. discharge current (mA)
Small	GMB 300910	12	10×9×3	0.34	4-5.8	120
Medium	GMB 361215	40	15×12×3.6	0.92	1.7-2.6	100
Large	GMB 402020	150	20×20×4.1	2.80	0.3-0.43	150

*Data are obtained from the manufacturer, except for the weight and internal resistance, which are measured from four different samples for each battery. The internal resistance depends on the current and details can be found in Supplementary Figs. 33-35. The maximum discharge current is recommended by the product manuals.

Supplementary Table 5. Summary of the SEMRs

Item / Name+	Body length (mm)	Weight (g)	Shape of feet	Internal resistance[a] (Ω)	PCB type	Battery	Endurance[b] (s)	Portrait	Tested feature
SEMR TST	9	0.17	Sawtooth-shaped	1	/	/		Fig. 1a, S13f, S14a-b	Walk, run, jump, swim
SEMR TL	9	0.19	L-shaped	1	/	/		Fig. 1a	Walk, run, jump
SEMR TSTS	9	0.19	Sawtooth-shaped	0.7	/	/		Fig. 4f	Walk, run, steer
SEMR TRC	20	1.42	Rectangular	3.6/4.6	/	/		Fig. 4g, S29b	Cargo transport and release
SEMR UL	9	0.71	L-shaped	7.6	Small	Small	270	Fig. 4h, S33c	Walk, run
SEMR UR1	20	2.2	Rectangular	4.6	Medium	Medium	400	Fig. 4h, S34b-f	Walk, run, swim
SEMR UR2	20	4.73	Rectangular	4.6	Large	Large	817	Fig. 4h, S35c-f	Swim

+ The names of the SEMRs include information on the method of supplying power ("T" for tethered or "U" untethered), the shape of feet ("ST" for saw tooth-shaped, "L" for L-shaped, "R" for rectangular), the tested feature ("S" for steering, "C" for cargo) and version number ("1" for No. 1, "2" for No. 2).

[a]SEMR TSTS has two individual coils, and each one has a resistance of about 0.7 Ω. SEMR TRC consists of two parts. The actuator has a resistance of 3.6 Ω and the body has a resistance of 4.6 Ω.

[b]The endurance is the operating time of the untethered SEMR, which is calculated from the power consumption of the robot and the battery capacity. Details can be found in the Supplementary text, sections 1.8.6-1.8.8.

Supplementary Table 6. Parameters of spin-coating process.

Revolutions per second (RPS)	Spinning time (s)	Thickness of the film (µm)
**25**	120	30
**20**	60	50
**15**	60	60-70
**10**	60	90-100
**8**	60	100-120
**5**<br>**8**	30<br>30	150
**3**<br>**8**	30<br>30	160
**3**<br>**5**	30<br>30	240
**4**<br>**5**	30<br>30	200
**5**	60	200
**4**	60	220-250
**3**<br>**4**	30<br>30	250-300
**3**	60	300-350
**2**	60	500-600
**1**	60	1000-1100

Note: the thickness could vary depending on the size of the substrate. The ramp time is 2 s for all cases.

Supplementary Table 7. Lorentzian oscillator parameters for representative curved beam geometries.

**Full half-beam arc angle ψ**	**$\psi \to 0$**		**$\pi/4$**		**$\pi/2$**		**$3\pi/4$**	
**Boundary conditions**	free-free	clamped-free	free-free	clamped-free	free-free	clamped-free	free-free	clamped-free
**Frequency pre-factor Ω_ψ**	5.593	3.516	5.176	3.560	4.533	3.697	4.219	3.935
**Effective mass ratio m_x**	$0.240/\psi^2$	$0.607/\psi^2$	0.511	1.078	0.240	0.361	0.237	0.272
**Effective mass ratio m_y**	$0.240/\psi$	$0.388/\psi$	0.456	0.593	0.754	0.567	2.455	3.822
**Amplitude pre-factor C_x**	0.959	0.899	0.960	0.904	0.965	0.921	0.976	0.950
**Amplitude pre-factor C_y**	0.959	0.945	0.964	0.958	0.996	1.013	0.801	1.69

Note: These dimensionless coefficients relate the key parameters of the vibrational frequency response to the materials and geometry of the curved robot, as described by the Eqs. (21), (25)-(27).

4. Supplementary References

1 Lukežič, A., Vojíř, T., Čehovin Zajc, L., Matas, J. & Kristan, M. Discriminative Correlation Filter Tracker with Channel and Spatial Reliability. *International Journal of Computer Vision* **126**, 671-688, doi:10.1007/s11263-017-1061-3 (2018).

2 Lai, W. M., Rubin, D. H., Rubin, D. & Krempl, E. *Introduction to continuum mechanics*. (Butterworth-Heinemann, 2009).

3 Zhao, X. & Suo, Z. Method to analyze programmable deformation of dielectric elastomer layers. *Applied Physics Letters* **93**, 251902 (2008).

4 Mao, G. *et al.* Soft electromagnetic actuators. *Science Advances* **6**, eabc0251, doi:10.1126/sciadv.abc0251 (2020).

5 Love, A. E. H. *A treatise on the mathematical theory of elasticity*. (Cambridge university press, 2013).

6 Lacarbonara, W. *Nonlinear structural mechanics: theory, dynamical phenomena and modeling*. (Springer Science & Business Media, 2013).

7 Vlajic, N., Fitzgerald, T., Nguyen, V. & Balachandran, B. Geometrically exact planar beams with initial pre-stress and large curvature: Static configurations, natural frequencies, and mode shapes. *International Journal of Solids and Structures* **51**, 3361-3371 (2014).

8 Yang, F., Sedaghati, R. & Esmailzadeh, E. Free in-plane vibration of curved beam structures: a tutorial and the state of the art. *Journal of Vibration and Control* **24**, 2400-2417 (2018).

9 Landau, L. D. & Lifshits, E. M. *Course of Theorical Physics: Theory of Elasticity*. (Pergamon press, 1986).

10 Korn, G. A. & Korn, T. M. *Mathematical handbook for scientists and engineers: definitions, theorems, and formulas for reference and review*. (Courier Corporation, 2000).

11 Anderson, B., Shultz, J. & Jayne, B. Axial kinematics and muscle activity during terrestrial locomotion of the centipede Scolopendra heros. *The Journal of Experimental Biology* **198**, 1185-1195 (1995).

12 Wittlinger, M., Wehner, R. & Wolf, H. The desert ant odometer: a stride integrator that accounts for stride length and walking speed. *Journal of Experimental Biology* **210**, 198, doi:10.1242/jeb.02657 (2007).

13 Full, R. J. & Tu, M. S. Mechanics of a rapid running insect: two-, four- and six-legged locomotion. *Journal of Experimental Biology* **156**, 215-231 (1991).

14 C. Christopher, A., Paul D, K. & Daniel R. Formanowicz, J. R. The Effects of Leg Autotomy on Running Speed and Foraging Ability in Two Species of Wolf Spider, (Lycosidae). *The American Midland Naturalist* **145**, 201-205, doi:10.1674/0003-0031(2001)145[0201:TEOLAO]2.0.CO;2 (2001).

15 Resources, I. D. o. N. *brushlegged wolf spider*, <https://www2.illinois.gov/dnr/education/CDIndex/BrushleggedWolfSpider.pdf> (2020).

16 Rubin, S., Young, M. H.-Y., Wright, J. C., Whitaker, D. L. & Ahn, A. N. Exceptional running and turning performance in a mite. *The Journal of Experimental Biology* **219**, 676-685, doi:10.1242/jeb.128652 (2016).

17 Hutchinson, J. R., Famini, D., Lair, R. & Kram, R. Are fast-moving elephants really running? *Nature* **422**, 493-494, doi:10.1038/422493a (2003).

18 Shoshani, J. & Eisenberg, J. F. Elephas maximus. *Mammalian species* (1982).

19 Hildebrand, M. Motions of the Running Cheetah and Horse. *Journal of Mammalogy* **40**, 481-495, doi:10.2307/1376265 (1959).

20 Benedict, M. What Is The Fastest Dog Breed – 20 Fastest Dog Breeds. (2020). <https://dogopedia.net/dog-facts/what-is-the-fastest-dog-breed-20-fastest-dog-breeds/>.

21 Anonymous. 5 Facts You May Not Know About Mice. (2017). <https://www.moxieservices.com/blog/5-facts-you-may-not-know-about-mice/#:~:text=In%20a%20world%20where%20we,running%20up%20to%20160%20mph.>.

22 O'Malley, C. I. How Fast Can A Cat Run? (2017).

23 Aoshima, S.-i., Tsujimura, T. & Yabuta, T. A Miniature Mobile Robot Using Piezo Vibration for Mobility in a Thin Tube. *Journal of Dynamic Systems, Measurement, and Control* **115**, 270-278, doi:10.1115/1.2899031 (1993).

24 Baisch, A. T., Ozcan, O., Goldberg, B., Ithier, D. & Wood, R. J. High speed locomotion for a quadrupedal microrobot. *The International Journal of Robotics Research* **33**, 1063-1082, doi:10.1177/0278364914521473 (2014).

25 Pierre, R. S., Gosrich, W. & Bergbreiter, S. in *Solid-State Sensors, Actuators, and Microsystems Workshop, Hilton Head, SC.*

26 Birkmeyer, P., Peterson, K. & Fearing, R. S. in *2009 IEEE/RSJ International Conference on Intelligent Robots and Systems.* 2683-2689.

27 Wu, Y. *et al.* Insect-scale fast moving and ultrarobust soft robot. *Science Robotics* **4**, eaax1594, doi:10.1126/scirobotics.aax1594 (2019).

28 Haldane, D. W., Peterson, K. C., Bermudez, F. L. G. & Fearing, R. S. in *2013 IEEE International Conference on Robotics and Automation.* 3279-3286.

29 Haldane, D. W. & Fearing, R. S. in *2015 IEEE International Conference on Robotics and Automation (ICRA).* 4539-4546.

30 Duduta, M., Clarke, D. R. & Wood, R. J. in *2017 IEEE International Conference on Robotics and Automation (ICRA).* 4346-4351.

31 Hu, W., Lum, G. Z., Mastrangeli, M. & Sitti, M. Small-scale soft-bodied robot with multimodal locomotion. *Nature* **554**, 81-85, doi:10.1038/nature25443 (2018).

32 Tang, Y. *et al.* Leveraging elastic instabilities for amplified performance: Spine-inspired high-speed and high-force soft robots. *Science Advances* **6**, eaaz6912, doi:10.1126/sciadv.aaz6912 (2020).

33 Li, T. *et al.* Agile and Resilient Insect-Scale Robot. *Soft Robotics* **6**, 133-141, doi:10.1089/soro.2018.0053 (2018).

34 KERMACK, K. A. The Propulsive Powers of Blue and Fin Whales. *Journal of Experimental Biology* **25**, 237-240 (1948).

35 Elsworth, P. G., Seebacher, F. & Franklin, C. E. Sustained swimming performance in crocodiles (Crocodylus porosus): effects of body size and temperature. *Journal of Herpetology*, 363-368 (2003).

36 Truijens, M. & Toussaint, H. Biomechanical aspects of peak performance in human swimming. *Animal Biology* **55**, 17-40 (2005).

37 Denil, G. in *Annales des travaux publics de Belgique.* 412-423.

38 Gray, J. The locomotion of fishes. *Essays in marine biology*, 1-16 (1953).

39 Lee, H.-J., Jong, Y.-J., Chang, L.-M. & Wu, W.-L. Propulsion strategy analysis of high-speed swordfish. *Transactions of the Japan Society for Aeronautical and Space Sciences* **52**, 11-20 (2009).

40 Wilson, R., James, R. & Johnston, I. Thermal acclimation of locomotor performance in tadpoles and adults of the aquatic frog Xenopus laevis. *Journal of Comparative Physiology B* **170**, 117-124 (2000).

41 Andersen, N. M. A Comparative Study of Locomotion on the Water Surface in Semiaquatic Bugs (Insecta, Hemiptera, Gerromorpha). (1976).

42 Kim, B., Kim, D.-H., Jung, J. & Park, J.-O. A biomimetic undulatory tadpole robot using ionic polymer–metal composite actuators. *Smart Materials and Structures* **14**, 1579-1585, doi:10.1088/0964-1726/14/6/051 (2005).

43 Song, Y. S. & Sitti, M. Surface-Tension-Driven Biologically Inspired Water Strider Robots: Theory and Experiments. *IEEE Transactions on Robotics* **23**, 578-589, doi:10.1109/TRO.2007.895075 (2007).

44 Wang, X. *et al.* Untethered and ultrafast soft-bodied robots. *Communications Materials* **1**, 67, doi:10.1038/s43246-020-00067-1 (2020).

45 Li, T. *et al.* Fast-moving soft electronic fish. *Science Advances* **3**, e1602045, doi:10.1126/sciadv.1602045 (2017).

46 Zhao, Q. *et al.* Fast-moving piezoelectric micro-robotic fish with double caudal fins. *Robotics and Autonomous Systems* **140**, 103733, doi:https://doi.org/10.1016/j.robot.2021.103733 (2021).

47 Wang, Z., Hang, G., Li, J., Wang, Y. & Xiao, K. A micro-robot fish with embedded SMA wire actuated flexible biomimetic fin. *Sensors and Actuators A: Physical* **144**, 354-360 (2008).

48 Bujard, T., Giorgio-Serchi, F. & Weymouth, G. D. A resonant squid-inspired robot unlocks biological propulsive efficiency. *Science Robotics* **6**, eabd2971, doi:10.1126/scirobotics.abd2971 (2021).

49 Zhang, S., Qian, Y., Liao, P., Qin, F. & Yang, J. Design and Control of an Agile Robotic Fish With Integrative Biomimetic Mechanisms. *IEEE/ASME Transactions on Mechatronics* **21**, 1846-1857, doi:10.1109/TMECH.2016.2555703 (2016).

50 Zhu, J. *et al.* Tuna robotics: A high-frequency experimental platform exploring the performance space of swimming fishes. *Science Robotics* **4**, eaax4615, doi:10.1126/scirobotics.aax4615 (2019).

51 Diller, E., Zhuang, J., Lum, G. Z., Edwards, M. R. & Sitti, M. Continuously distributed magnetization profile for millimeter-scale elastomeric undulatory swimming. *Applied Physics Letters* **104**, 174101, doi:10.1063/1.4874306 (2014).
