# Supplementary material for: Ultrafast small-scale soft electromagnetic robots
Source: Nat Commun. 2022 Aug 9;13:4456. doi: 10.1038/s41467-022-32123-4 (PMC9363453; doi:10.1038/s41467-022-32123-4)
Supplement: Supplementary file 3 — Description of Additional Supplementary Files [file 41467_2022_32123_MOESM3_ESM.pdf]

Description of Additional Supplementary Files

File Name: Supplementary Movie 1
Description: **Dynamic bending of straight SEMAs.** The SEMA is positioned upright on the magnet. SEMA 1 is a solid, flat elastomer film with an embedded LM channel, while SEMA 2 has a rectangular hole in the middle of the film to make it lighter and nimbler. All the SEMAs are driven by square-wave currents, with the frequencies and amplitudes indicated in the video.

File Name: Supplementary Movie 2
Description: **Vibration testing.** The square-wave current sweeps the range from 1 to 100 Hz at 1 Hz increments, whereas each frequency is applied for 1 s. The SEMR TST is clamped in the middle by copper wires to a plastic frame. The suspension point is about 11 mm above the surface of the magnet. The second part of the video is high-speed camera footage played back at 100x slow motion to show the vibration of the robot in response to two square-wave currents (0.2 A, 37 Hz) and (0.5 A, 37 Hz).

File Name: Supplementary Movie 3
Description: **Walking and ultrafast running.** The surfaces for the SEMR TST and TL are 3D printed substrate and glass, respectively. The walking SEMR TST is driven by a square-wave current (0.3 A, 1 Hz). The running SEMR TST is driven by square-wave currents, (0.3 A, 55 Hz) and (0.5 A, 45 Hz). The walking SEMR TL is driven by a square-wave current (0.2 A, 1 Hz). The running SEMR TL is driven by square-wave currents, (0.3 A, 40 Hz) and (0.4 A, 30 Hz). Various substrates are tested at the end of the video. We also slowed the playback down ten times to track the running robot better.

File Name: Supplementary Movie 4
Description: **Locomotion with high currents.** The SEMR TST is used in this video. At the beginning of this video, the SEMR fails to run, when actuated by a high-amplitude square-wave current (0.6 A, 30 Hz, Fig. S22a). Next, the SEMR is driven with the asymmetric current I (Fig. S22b) and asymmetric current II (Fig. S22c), resulting in reproducible SEMR locomotion. The asymmetric current I has an amplitude of 0.6 A, but within one period of 25 ms the duration of positive current is 16.7 s and that of negative current is 8.3 ms. The asymmetric current II has a frequency of 30 Hz, but the amplitude of the positive current is 0.6 A, while that of the negative current is 0.3 A. Finally, the SEMR is driven by a high-frequency square-wave current (0.6 A, 100 Hz), exhibiting slow locomotion.

File Name: Supplementary Movie 5
Description: **Robustness test.** In the first part of the video, the SEMR TL recovers its operational moving abilities, after being manually depressed and flattened by a plastic bar. The driving square-wave current is 0.2 A at 1 Hz. In the second part, the body of SEMR TL is compressed by a tensile test machine up to a maximum force of 10 N and recovers after the test.

File Name: Supplementary Movie 6
Description: **Jump test.** The SEMR TST jumps onto a platform upon application of a pulsed current, consisting of a 100 ms long, negative 1 A current, followed by a 50 ms long, positive 1 A current. In the first example, the platform surface is 3 mm above the ground, and the SEMR rolls over and starts to jump. In the second example, the platform is 4 mm high, and the SEMR first turns upside-down and then starts to jump.

The running SEMR TL is driven by a square-wave current, 0.3A at 40 Hz. A 20 ms long, negative 1 A current followed by a 30 ms long, positive 1 A current, is applied to the SEMR TL to let it jump over the obstacle or jump onto the stage. The obstacle and the stage are both 2.5 mm high, about half of the height of the SEMR TL.

File Name: Supplementary Movie 7
Description: **Swim test.** The SEMR is put on a water surface inside the Petri dish. The diameter of the Petri dish is 8.5 cm and the water depth is about 1 cm. The Petri dish is placed on top of the magnet. The SEMR TST is actuated with a square-wave current (0.5 A, 20 Hz). Two digital cameras recorded the videos from the top and the side separately.

File Name: Supplementary Movie 8
Description: **A two-module steering SEMR.** First, a square-wave signal (0.5 A, 80 Hz) is applied to both coils simultaneously, causing the robot to walk straight. Then the power is turned off. A different square-wave signal (0.5 A, 50 Hz) is applied only to the left coil. The SEMR TSTS turns clockwise. Then the power is turned off again. The same square-wave signal (0.5 A, 50 Hz) is applied only to the right coil. The SEMR TSTS turns anticlockwise. Each signal lasts 0.5 seconds. The tick interval along the straight line is 1 cm.

File Name: Supplementary Movie 9
Description: **Cargo transportation and release.** The SEMR TRC with a release actuator transports cargo over a distance and ejects it at the destination point. The actuation (on/off) is controlled by a custom program with a square-wave current (0.5 A, 8 Hz).

File Name: Supplementary Movie 10
Description: **Running and swimming untethered SEMRs.** The SEMR UL and UR1 run at maximum speeds of 1.2 BL/s and 2.1 BL/s, respectively, in real time playback and 5x slow motion. The actuation frequencies of SEMR UL are 5 Hz and 11 Hz, as indicated in the video. The SEMR UR2 swims at a speed of about 1.8 BL/s in real-time playback and 5x slow motion.
